# Supplementary material for: Ecophysiological Features Shape the Distribution of Prophages and CRISPR in Sulfate Reducing Prokaryotes
Source: Microorganisms. 2021 Apr 27;9(5):931. doi: 10.3390/microorganisms9050931 (PMC8146710; doi:10.3390/microorganisms9050931)
Supplement: Supplementary file 1 [file microorganisms-09-00931-s001.zip › microorganisms-1162261-supplementary.pdf]

Supplementary Materials

Ecophysiological Features Shape the Distribution of Prophages and CRISPR in Sulfate Reducing Prokaryotes

Roberto Orellana[1], Alejandra Arancibia[1], Leonardo Badilla[1], Jonathan Acosta[3], Gabriela Arancibia[2], Rodrigo Escar[2], Gustavo Ferrada[2], Michael Seeger[2].

[1]Laboratorio de Biología Celular y Ecofisiología Microbiana. Facultad de Ciencias Naturales y Exactas. Universidad de Playa Ancha, Leopoldo Carvallo 270, Valparaíso, Chile. [2]Laboratorio de Microbiología Molecular y Biotecnología Ambiental, Departamento de Química & Centro de Biotecnología Daniel Alkalay-Lowitt, Universidad Técnica Federico Santa María, Avenida España 1680, Valparaíso, Chile. [3] Departamento de Estadística, Pontificia Universidad Católica de Chile, Chile.

Supplementary Materials: The following are available online at www.mdpi.com/xxx/s1.

Figures S1, S1.1 – S1.83, Phylogenetic trees of 16S rRNA gene sequences of SBR strains and microbial strains belonging to the training set of PhiSpy.

Figure S2: Effect of Mitomycin C on the growth of (a) *D. vulgaris* str. Hildenborough, (b) *D. africanus* 2603 and (c) *D. acrylicus* DSM 10141. White circles indicate cultures grown in the absence of Mitomycin C; orange circles, cultures grown in the presence of 1 µg ml^{-1} of Mitomycin C; red circles, cultures grown in the presence of 3.5 µg ml^{-1} of Mitomycin C. In (a), black triangles indicate cultures grown in the presence of 10 µg ml^{-1} of Mitomycin C. Data represent the mean±sd from three independent replicate cultures. Bars designate one standard deviation of the mean. The table in the upper right section indicate presence (+) or absence (-) of prophages and CRISPR. (d) TEM micrographs of viral-like particles with typical morphotype of *Podoviridae* were obtained from *D. vulgaris* str. Hildenborough batch cultures after induction with Mitomycin C. (e-f) TEM micrographs presents structures that resembles outer membrane vesicles previously observed in *D. alaskensis* DSM16109. Most of the micrographs obtained in this study contained low-resolution viral particles and structures, cell debris, and a noisy background as a result of the interactions of the compounds of the growth medium of SRP.

Figure S3A: Effect of filtrates from mitomycin induced SRB cultures added at early exponential phase on the growth of *Desulfovibrio acrylicus* DSM 10141 (A-C), *Desulfovibrio vulgaris* str. Hildenborough (D-F), and *Desulfovibrio africanus* DSM 2603 (G-I).

Figure S3B: Effect of filtrates from mitomycin induced SRB cultures added at early stationary phase on the growth of *Desulfovibrio acrylicus* DSM 10141 (A-C), *Desulfovibrio vulgaris* str. Hildenborough (D-F), and *Desulfovibrio africanus* DSM 2603 (G-I).

Figure S4: Frequencies of integrated prophages (light red) and CRISPR arrays (light blue) in the genomes of SRP strains.

Figure S5: Box-plots of (A) DNA prophage per cell (volume) and (B) prophage density of lysogens belonging to five families of SRP; (C) red bars indicate the median of the length of prophages and (D) represents the median cellular volume and median genome size found in each family. Box-plots of (E) CRISPR DNA per cell and (F) CRISPR density of CRISPR-carrying strains belonging to five families of SRP; (G) light blue bars indicate the median of number of spacers belonging to strains of each family.

Figure S6: Distribution of prophages (red) and CRISPR (blue) according to different ecophysiological traits. In the left top (A), it is shown the distribution of prophages and CRISPR arrays according to the type of growth (doubling time $\leq$ 12 hours or $>$ 12 hours, as fast-growing or slow-growing strains, respectively). In the left bottom (B), it is shown the distribution of prophages and CRISPR arrays according to the presence of flagellum, classified as strains with flagellum (Y), strains for which flagellum has not been observed (N) and strains with no information (nd). In the right (C), it is shown the distribution of prophages and CRISPR arrays according to type of environment in which strains were isolated or the source of material that allowed the reconstruction of the genomes.

Figure S7: Comparison of genome annotations among SRP prophages. Pie charts indicate the annotation of prophages from *Desulfocurvibacter africanus* 2603, *Desulfovibrio vulgaris* str. Miyazaki, *Desulfovibrio vulgaris* str. Hildenborough and *Desulfovibrio vulgaris* DP4. The inner circle indicates functional classification (see color bar) and outer circle indicates the classification in the following categories; genes with bacterial origin (light green), genes with viral origin (light orange) and genes with unknown function (light gray).

Figure S8: (A) Network shows the degree of homology among SRP prophages. Circles indicate each SRP, colored by the taxonomy of the host. Edges show the presence of homologous genes among prophages. (B) Network shows the degree of homology among SRP prophages that share more than 10 homologs. The width of edges indicates the number of homologous genes between two prophages.

Figure S9: Whole-genome dot plot comparison of prophage nucleotides sequences of *Desulfovibrio* strains, prophages 1R1, 1R5 and 1R6 of *D. vulgaris* Hildenborough, 2R3 of *Desulfovibrio africanus* DSM 260, 5R2 of *Desulfovibrio aespoeensis* Aspo-2, 14R1 of *Desulfovibrio vulgaris* str. Miyazaki, 18R1 of *Desulfovibrio fructosivorans* JJ, 28R1 of *Desulfovibrio* sp. X2, 44R1 of *Desulfovibrio alaskensis* G20, and 90R2 and 90R3 of *Desulfovibrio vulgaris* DP4. Lack of grouping in dot plots are both reflection of the overall high degree of diversity and low level of synteny of the SRP prophages. The symbols indicate the classification of each prophage according to VIRFAM. The numbers in the right and bottom indicate the length of each prophage (in bp).

Table S1: CRISPR and putative CRISPR arrays identified in plasmids of SRP analysed in this study.

Table S2. List of prophages predicted by Phaster and PhiSpy. Columns indicate the following; total number of predicted prophages per host, prophage ID, prophage sequence ID, the start and end of the prophage in the genome, the length of the prophage (bp), the number of ORF, total prophage length (bp), average of the length, genome size of the host, prophage density, total prophage density, and taxonomic classification according to Virfam. Prophage density represents the portion of the host genome that contain each prophage (bp prophage * bp^{-1}host*100).

Table S3: Ecophysiological traits of SRP. The columns show the different SRP in this study, pH range, temperature range, classification with respect to temperature, cell wall type (gram), motility (denoted with Y for those for which the flagellum has been observed and N for which it has not been observed), habitat type, growth type under optimal conditions, oxidation (complete or incomplete), size (um^2), cell volume (μm^3) and cell surface area.

Table S4: List of CRISPR elements found by CRISPRCas-Finder. The columns indicate the following: The number assigned to each strain in this study, strain name, CRISPR sequence ID, CRISPR ID (named by CRISPRCas-Finder), beginning and end of each CRISPR array, consensus repeat, repeat length, number of spacers, level of evidence and the total size of each CRISPR found.

Table S5: List of CRISPR elements found inside prophages. The columns indicate the strain name, the prophage sequence ID, the beginning and end of the prophage in the genome, CRISPR sequence ID and the beginning and end of CRISPR in the nucleotide sequence.

Table S6: Classification of prophages according to Virfam. Columns indicate the following: Prophage ID, classification assigned by Virfam using head-neck-tail module genes, the length of the prophage (bp) and the name of the host.

Table S7: Pairwise amino acid comparison of all predicted open reading frames (ORFs) from the SRP prophages. The abbreviation used is the following: pident, percentage of identical matches, length, alignment length, mismatch, number of mismatches, gaopen, number of gap openings, qstart, start of alignment in query, qend, end of alignment in query, sstart, start of alignment in subject, send, end of alignment in subject, and evalue, expected value.

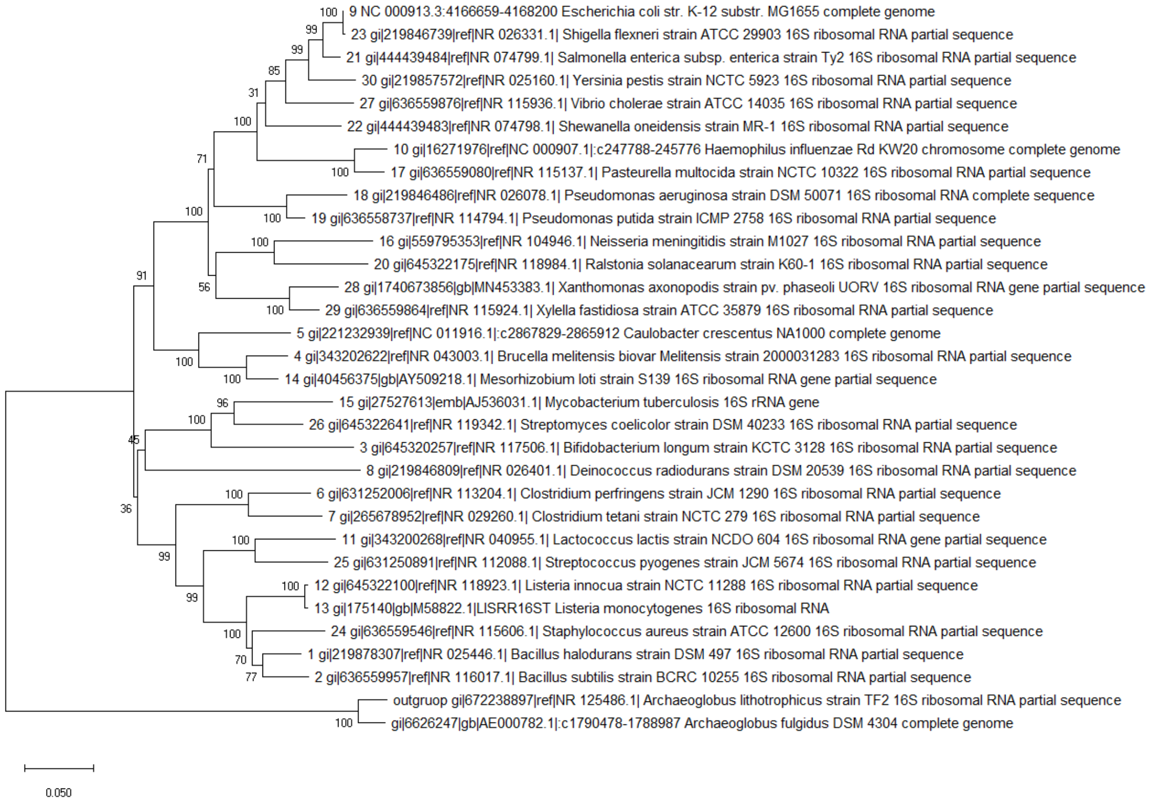

Figure S1.1: Phylogenetic tree of 16S rRNA gene sequences of *Archaeoglobus fulgidus* DSM 4304 and microbial strains belonging to the training set of PhiSpy.

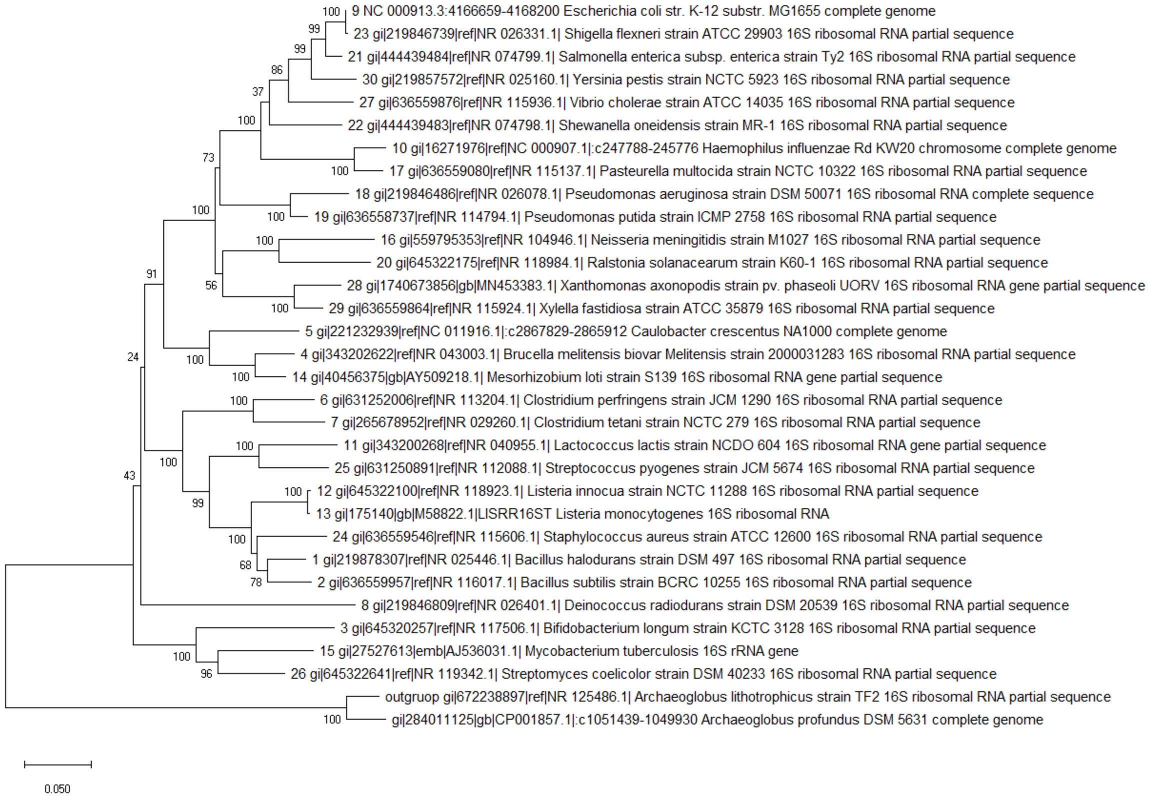

Figure S1.2: Phylogenetic tree of 16S rRNA gene sequences of *Archaeoglobus profundus* DSM 5631 and microbial strains belonging to the training set of PhiSpy.

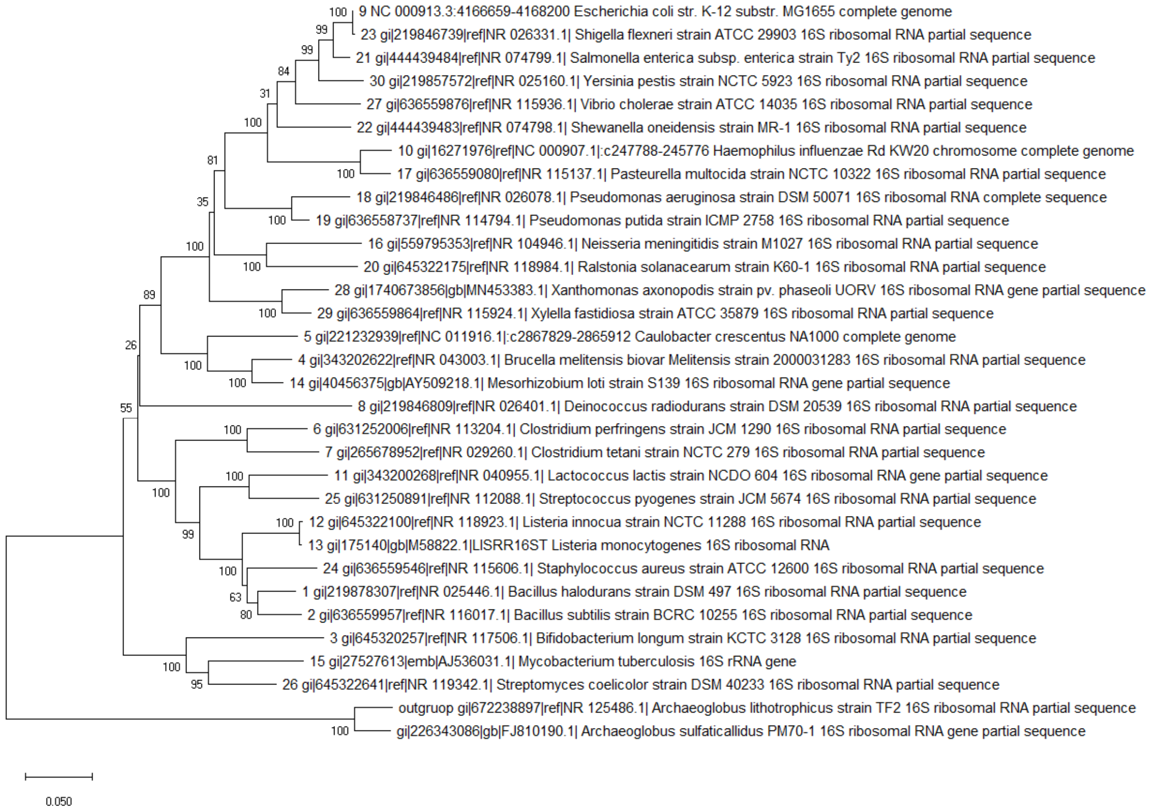

Figure S1.3: Phylogenetic tree of 16S rRNA gene sequences of *Archaeoglobus sulfaticallidus* PM70-1 and microbial strains belonging to the training set of PhiSpy.

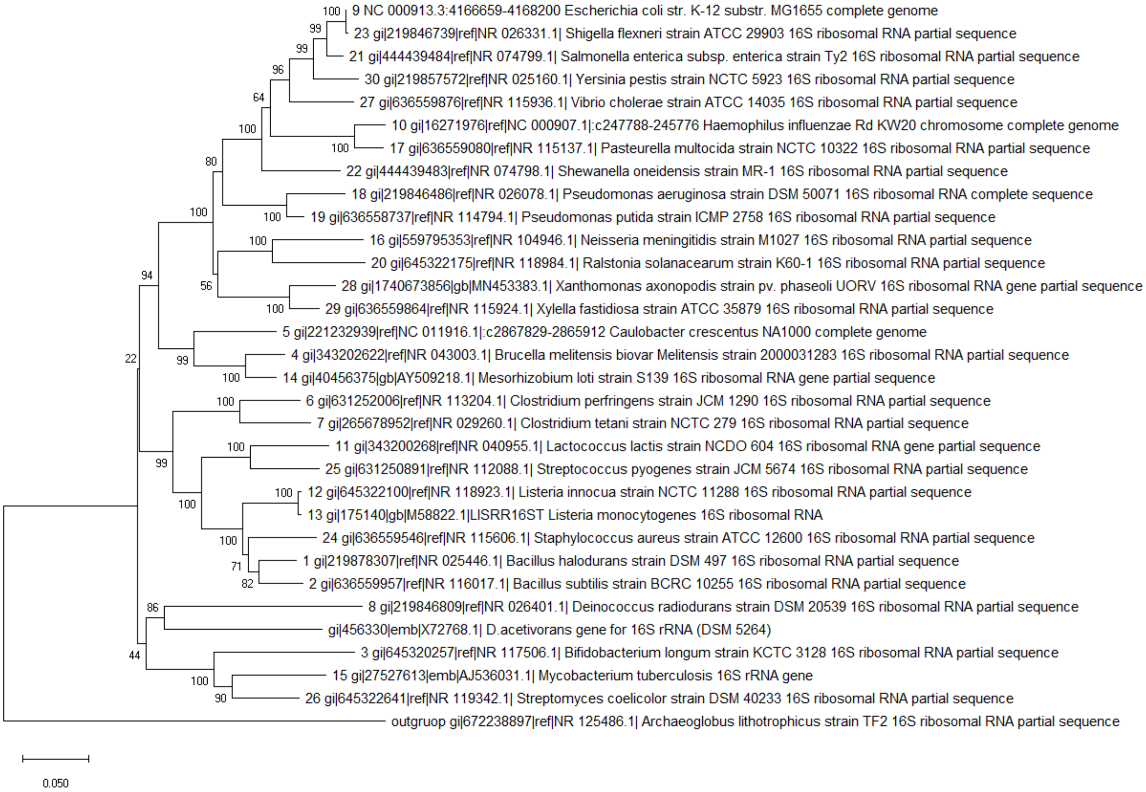

Figure S1.4: Phylogenetic tree of 16S rRNA gene sequences of *D.acetivorans* and microbial strains belonging to the training set of PhiSpy.

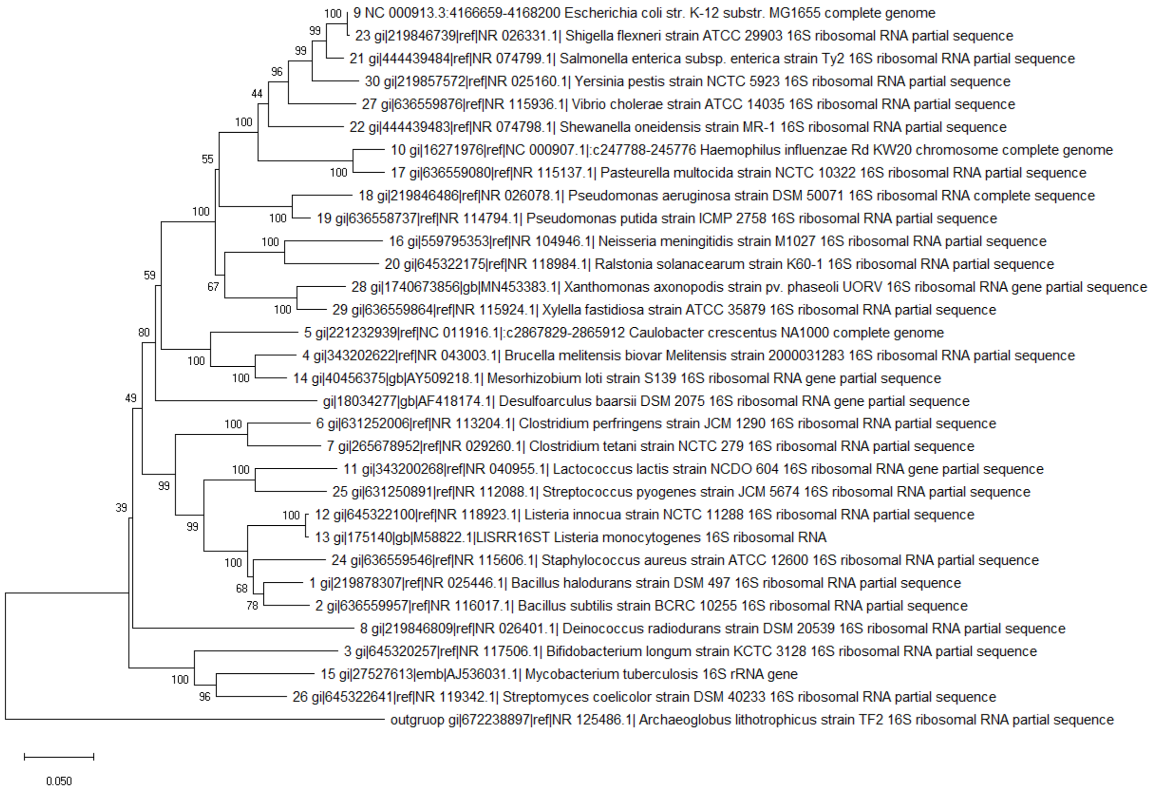

Figure S1.5: Phylogenetic tree of 16S rRNA gene sequences of *Desulfarculus baarsii* DSM 2075 and microbial strains belonging to the training set of PhiSpy.

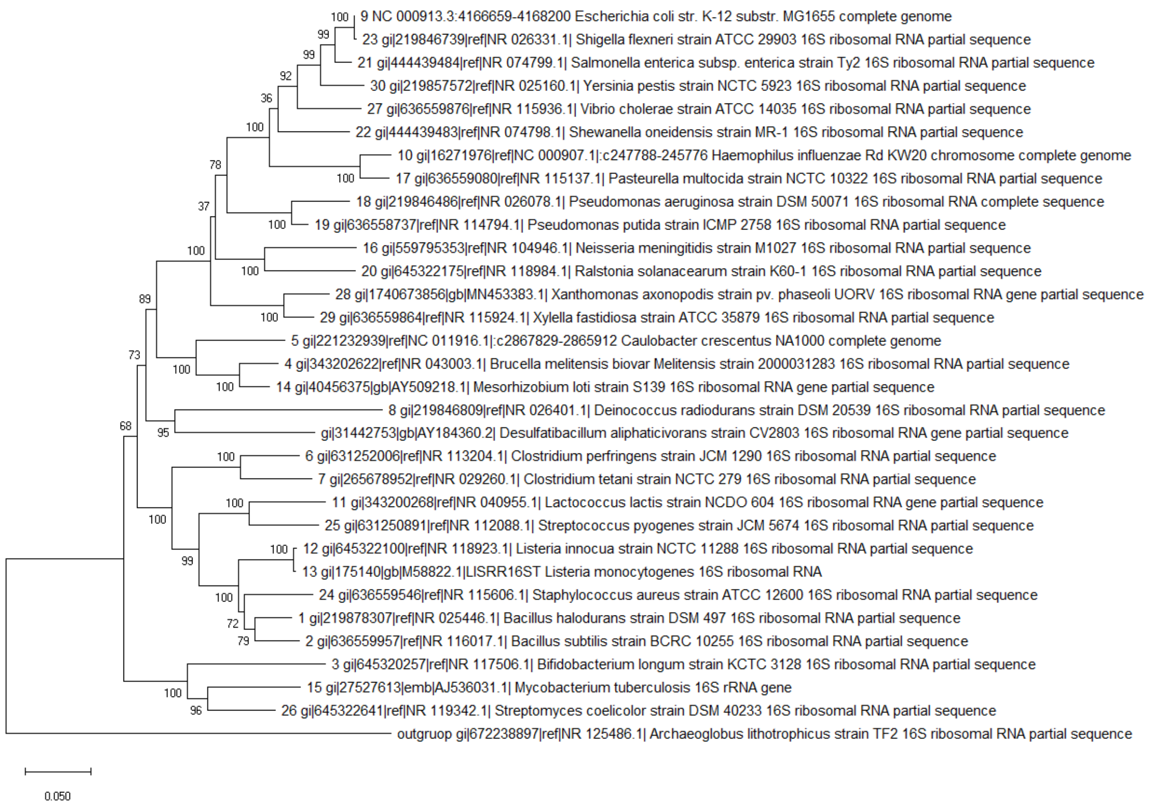

Figure S1.6: Phylogenetic tree of 16S rRNA gene sequences of *Desulfatibacillum aliphaticivorans* DSM 15576 and microbial strains belonging to the training set of PhiSpy.

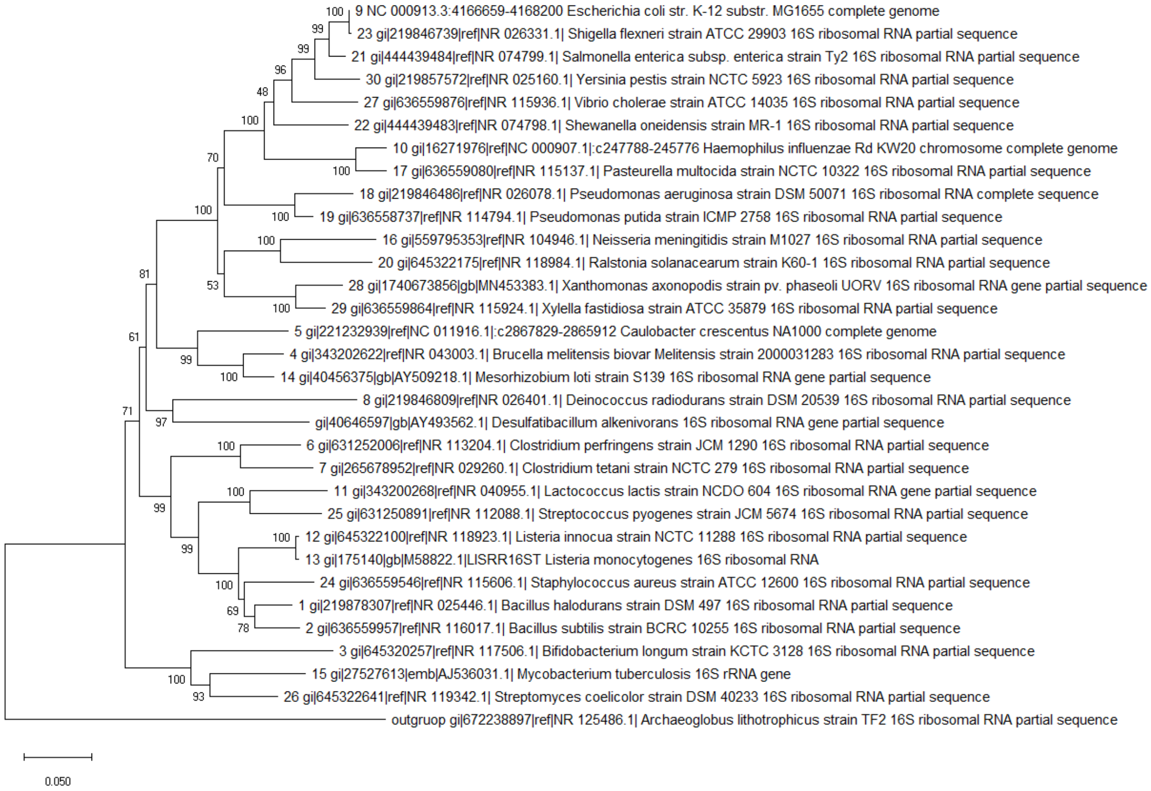

Figure S1.7: Phylogenetic tree of 16S rRNA gene sequences of *Desulfatibacillum alkenivorans* and microbial strains belonging to the training set of PhiSpy.

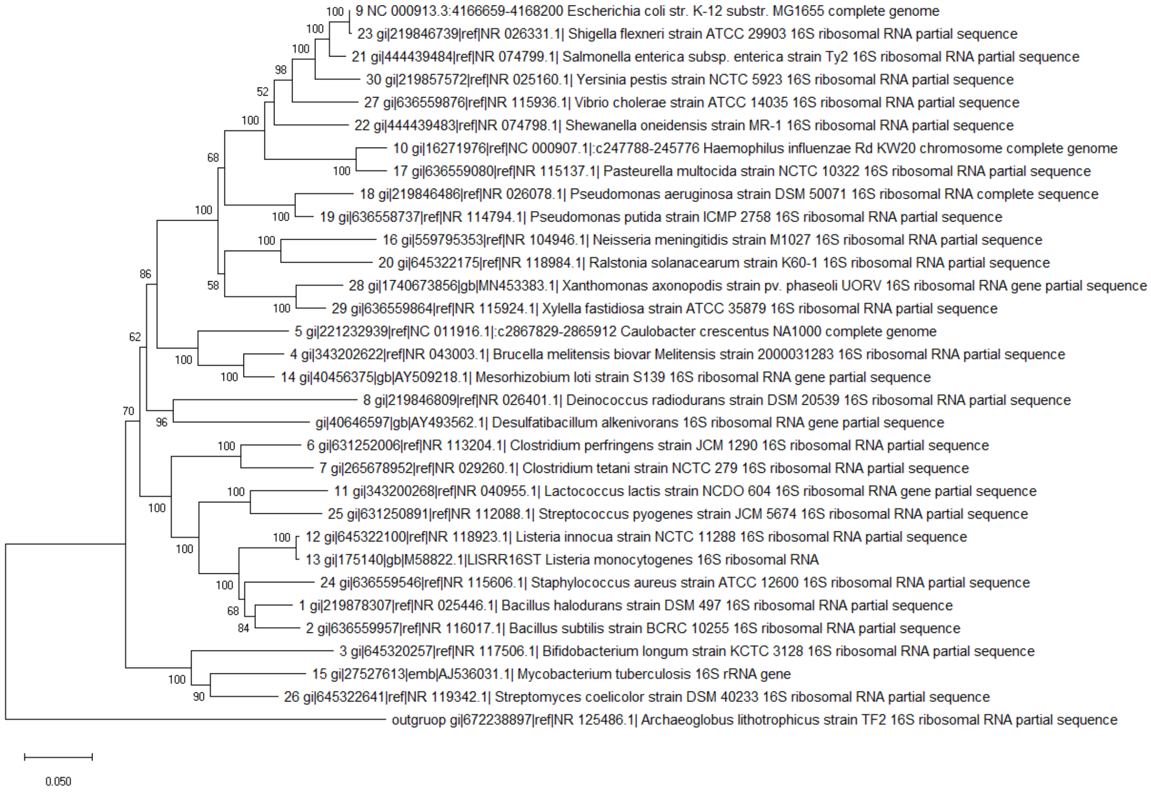

Figure S1.8: Phylogenetic tree of 16S rRNA gene sequences of *Desulfatibacillum alkenivorans* DSM 16219 and microbial strains belonging to the training set of PhiSpy.

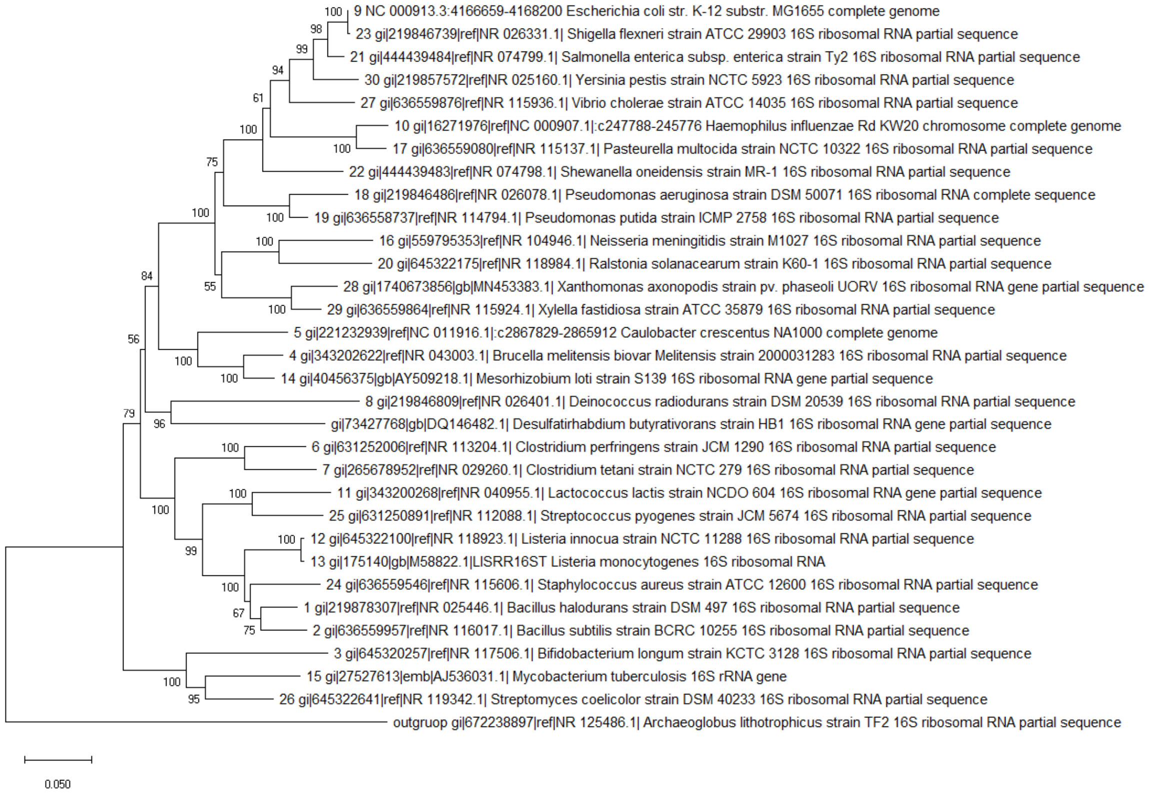

Figure S1.9: Phylogenetic tree of 16S rRNA gene sequences of *Desulfatirhabdium butyrativorans* DSM 18734 and microbial strains belonging to the training set of PhiSpy.

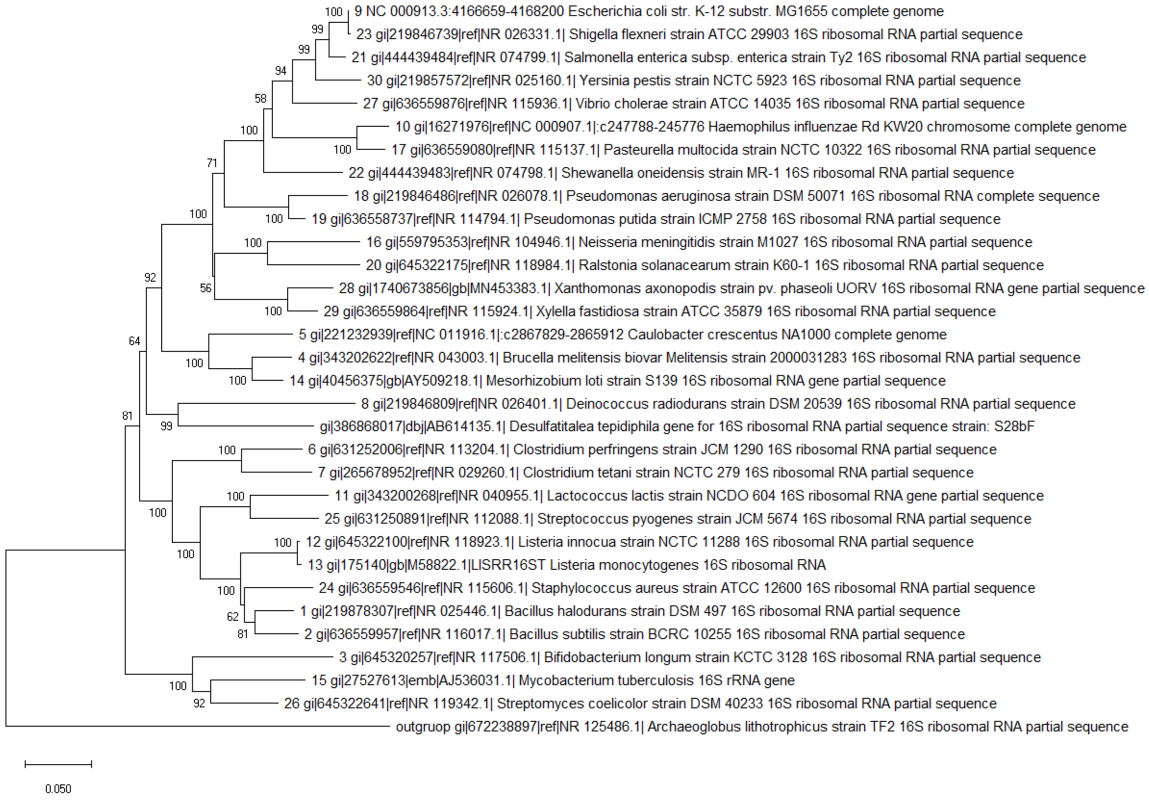

Figure S1.10: Phylogenetic tree of 16S rRNA gene sequences of *Desulfatitalea sp.* BRH_c12 and microbial strains belonging to the training set of PhiSpy.

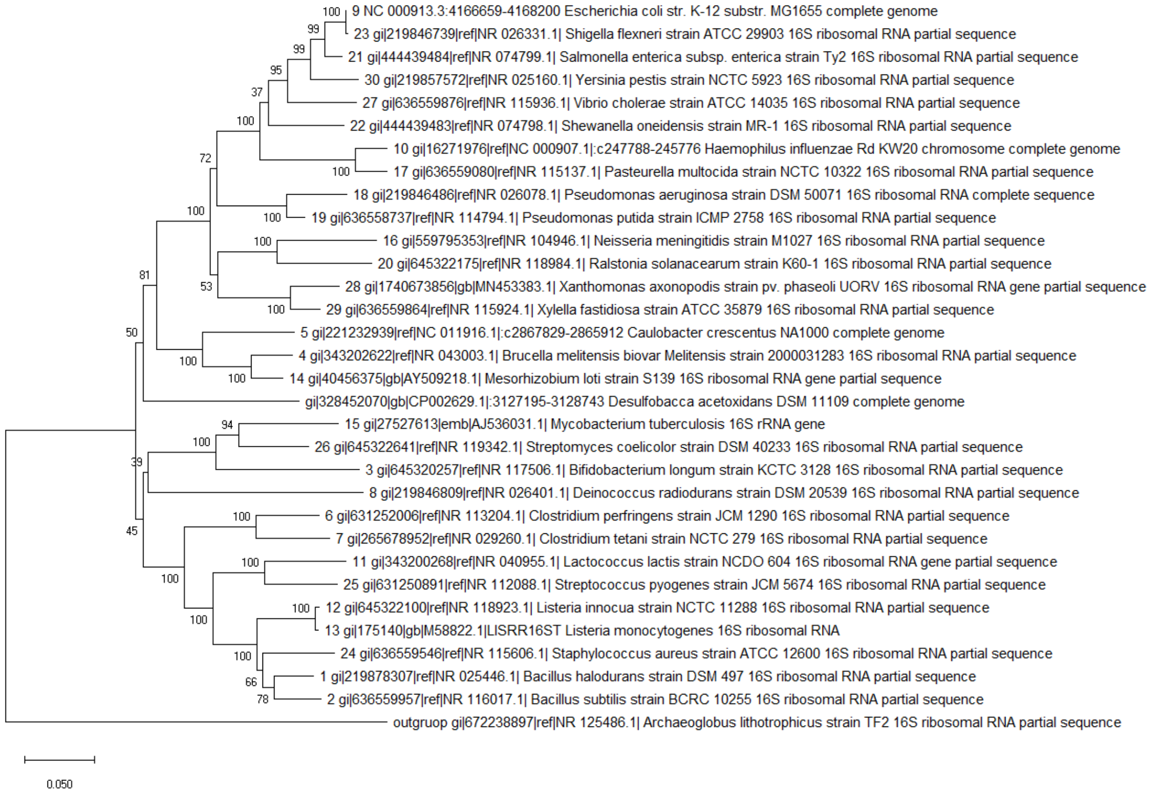

Figure S1.11: Phylogenetic tree of 16S rRNA gene sequences of *Desulfobacca acetoxidans* DSM 11109 and microbial strains belonging to the training set of PhiSpy.

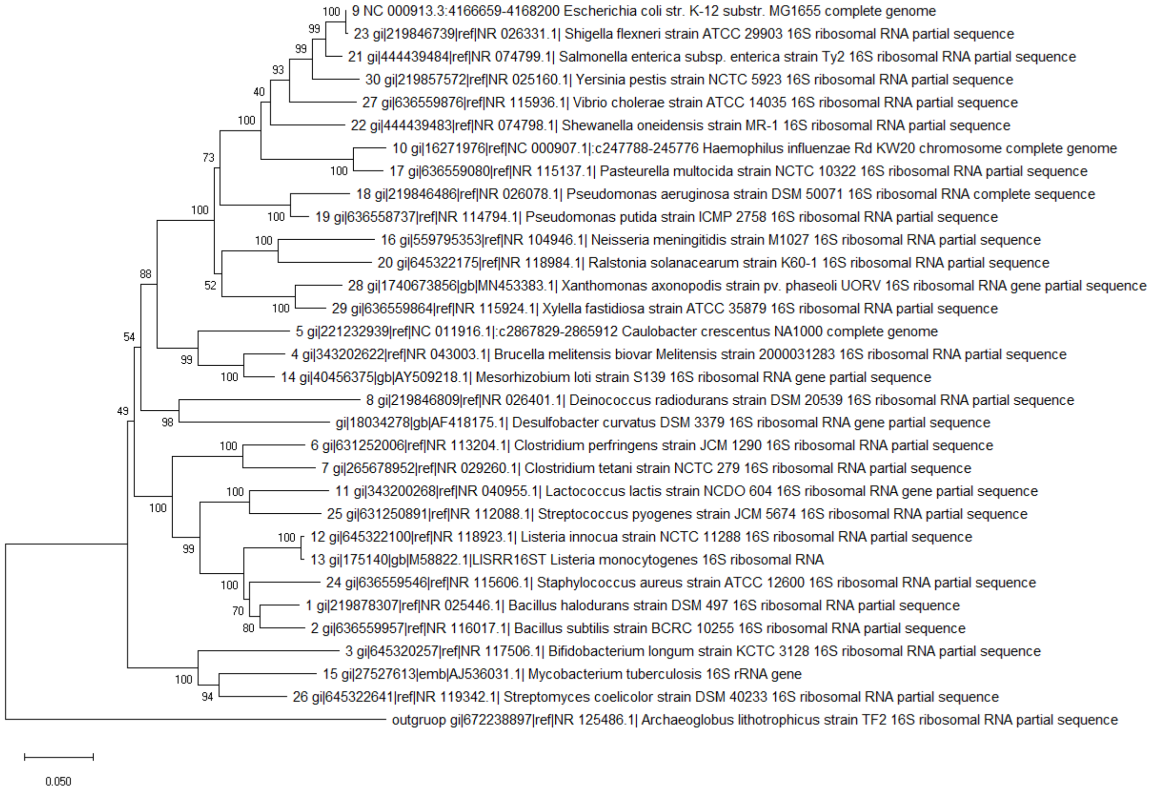

Figure S1.12: Phylogenetic tree of 16S rRNA gene sequences of *Desulfobacter curvatus* DSM 3379 and microbial strains belonging to the training set of PhiSpy.

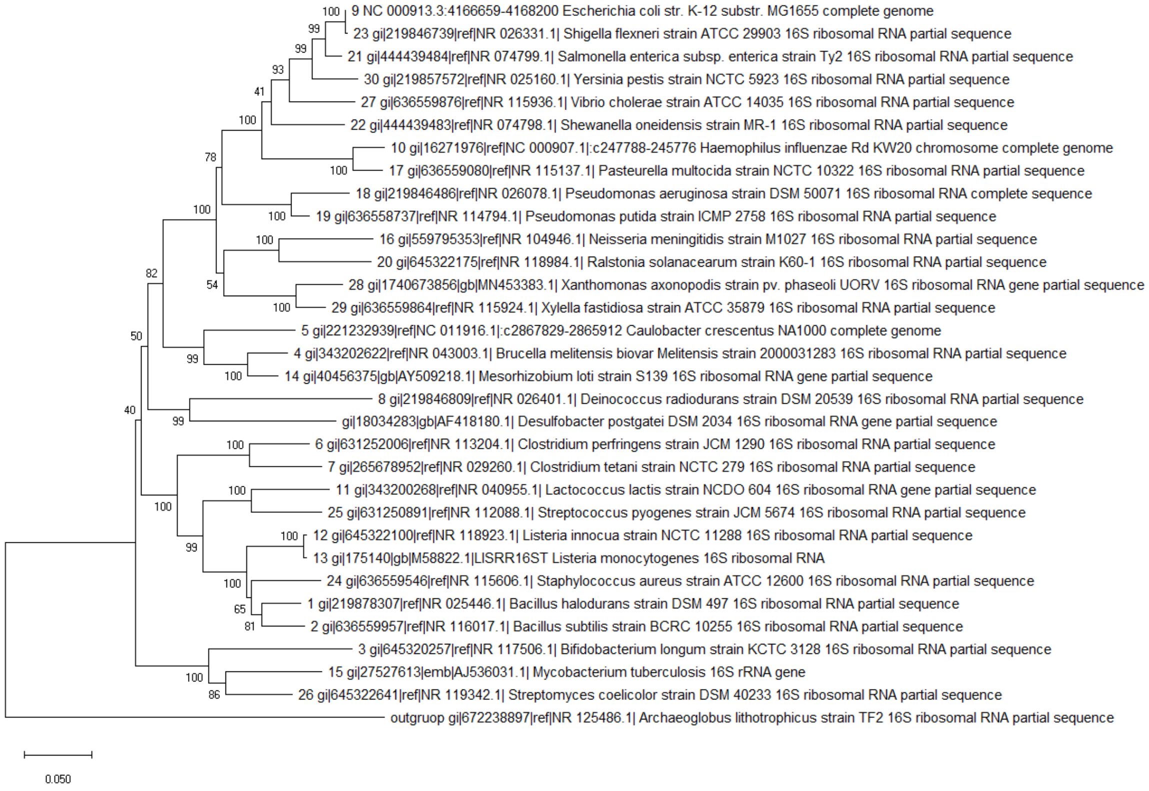

Figure S1.13: Phylogenetic tree of 16S rRNA gene sequences of *Desulfobacter postgatei* DSM 2034 and microbial strains belonging to the training set of PhiSpy.

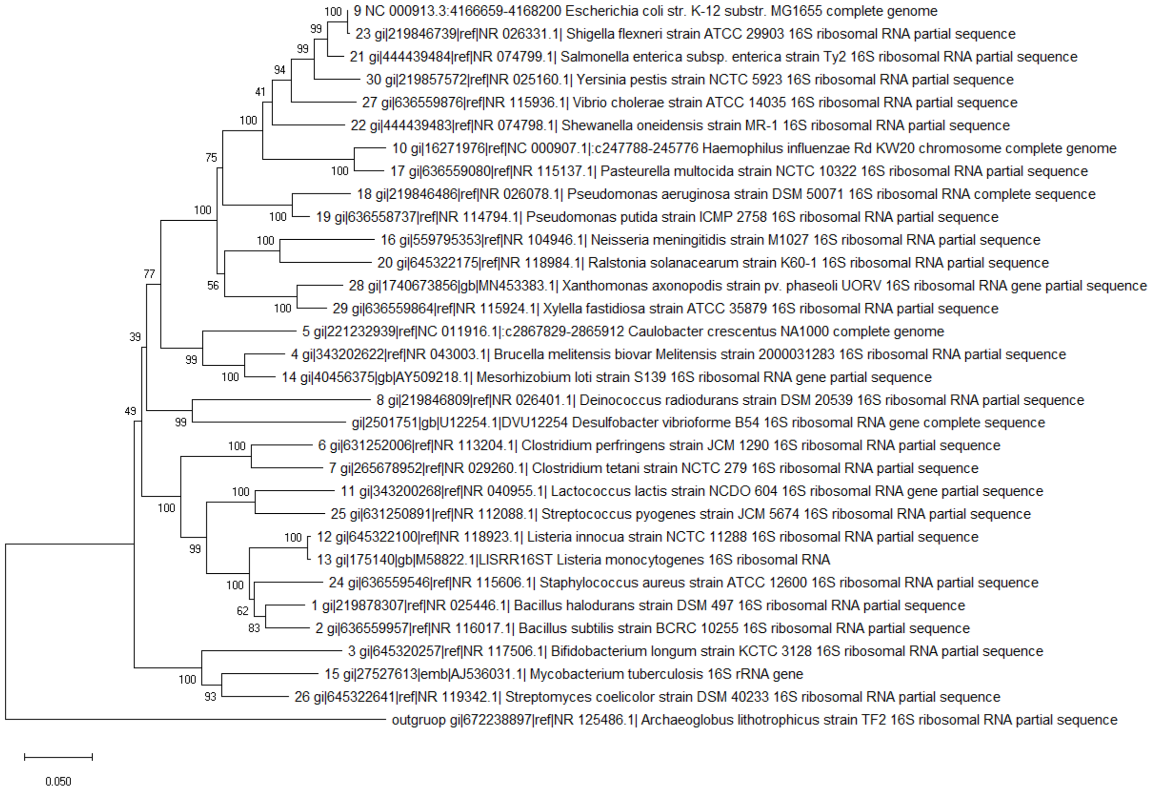

Figure S1.14: Phylogenetic tree of 16S rRNA gene sequences of *Desulfobacter vibrioforme* B54 and microbial strains belonging to the training set of PhiSpy.

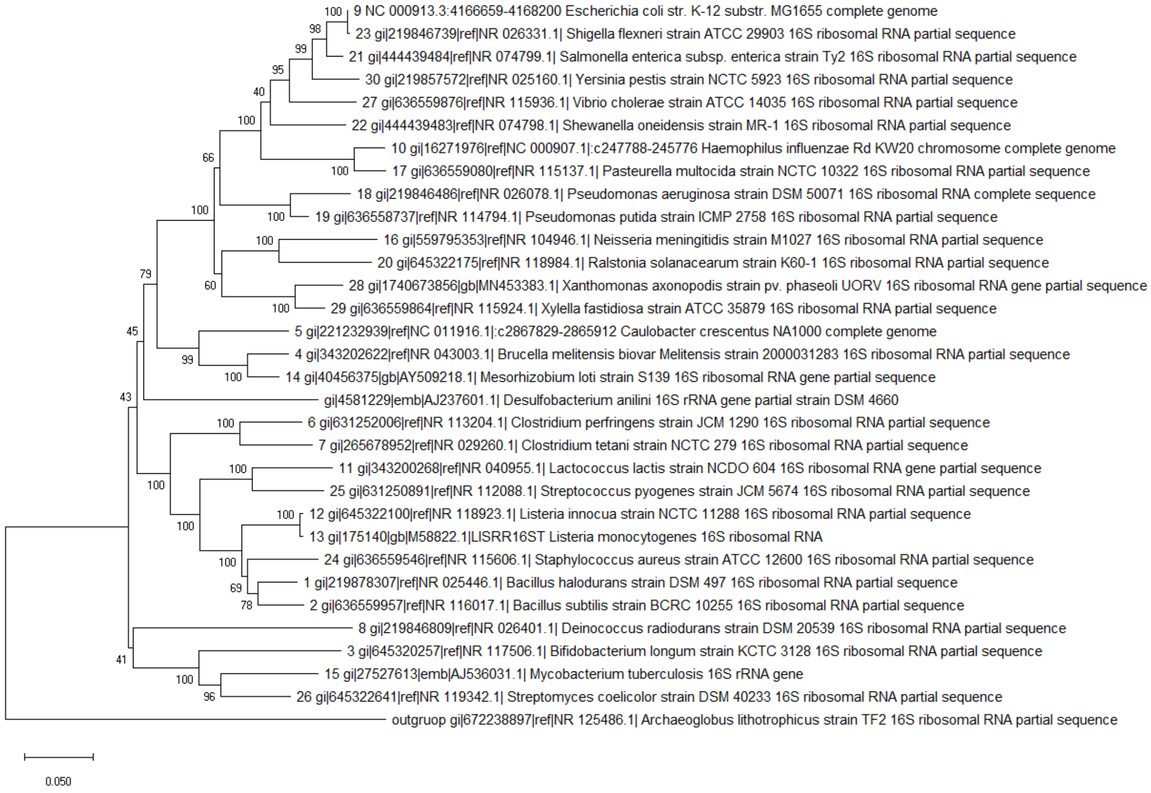

Figure S1.15: Phylogenetic tree of 16S rRNA gene sequences of *Desulfobacterium anilini* and microbial strains belonging to the training set of PhiSpy.

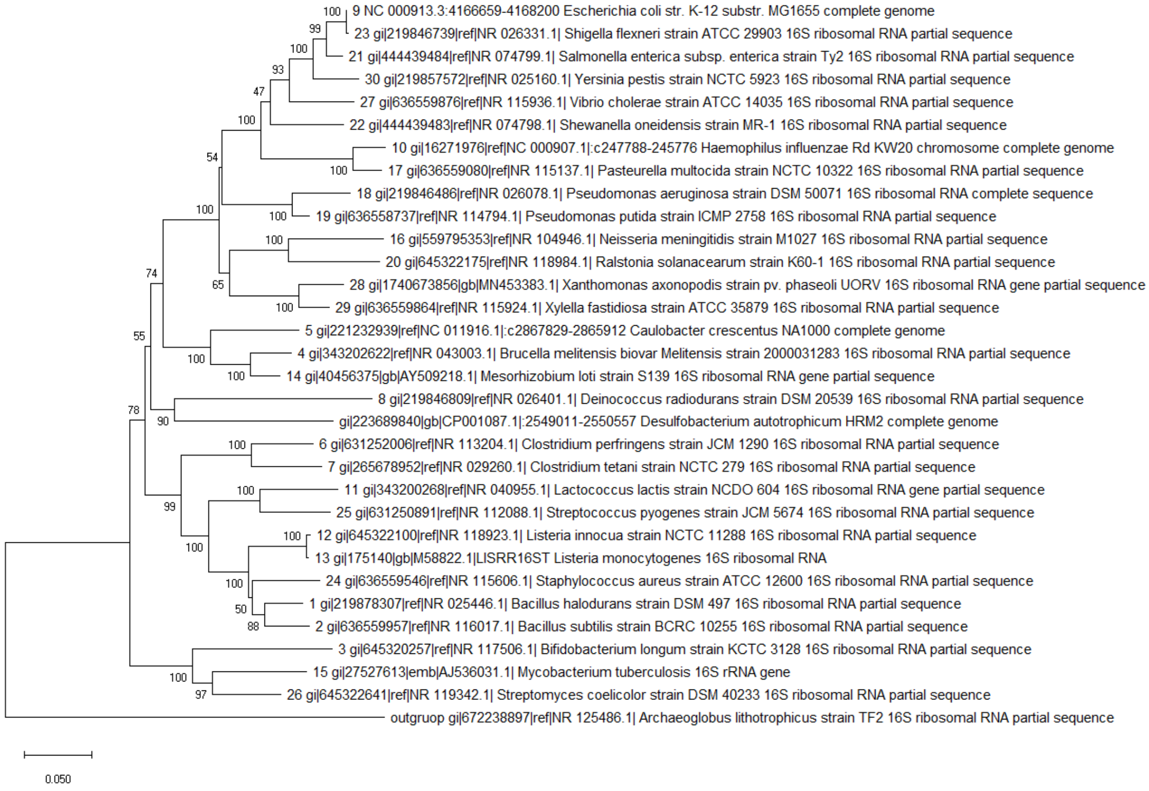

Figure S1.16: Phylogenetic tree of 16S rRNA gene sequences of *Desulfobacterium autotrophicum* HRM2 and microbial strains belonging to the training set of PhiSpy.

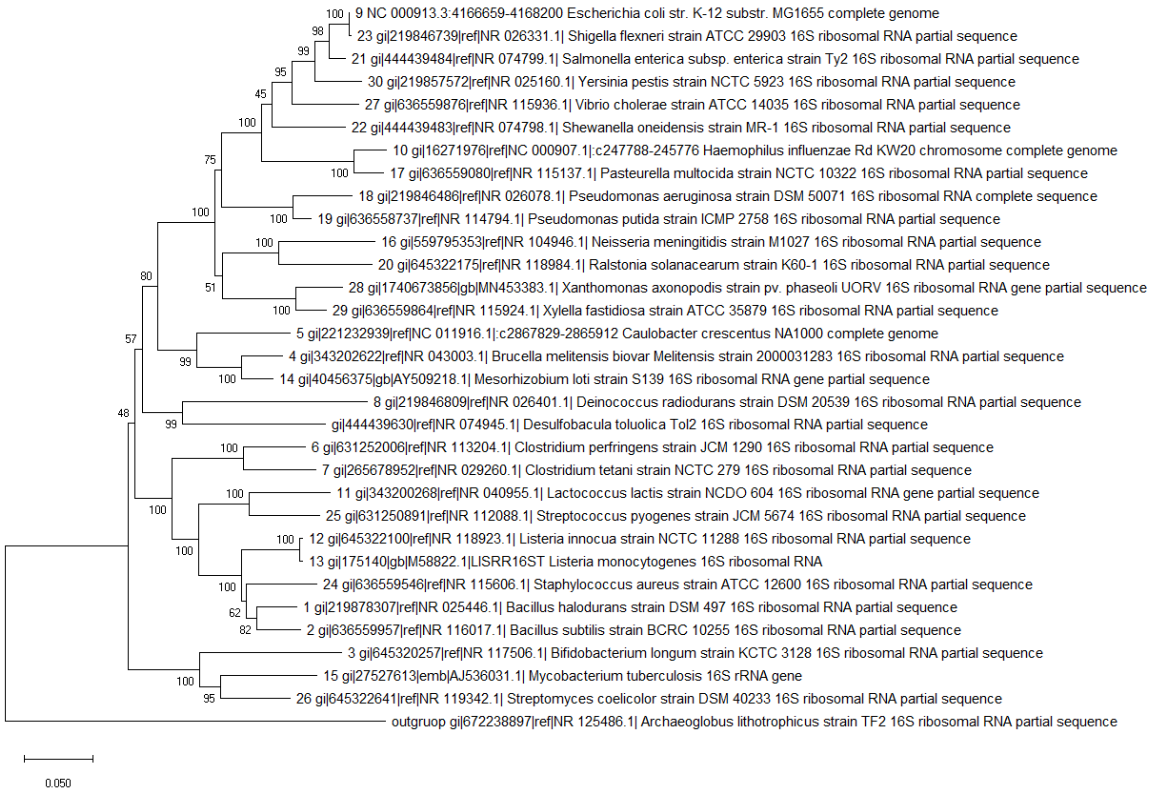

Figure S1.17: Phylogenetic tree of 16S rRNA gene sequences of *Desulfobacula toluolica* Tol2 and microbial strains belonging to the training set of PhiSpy.

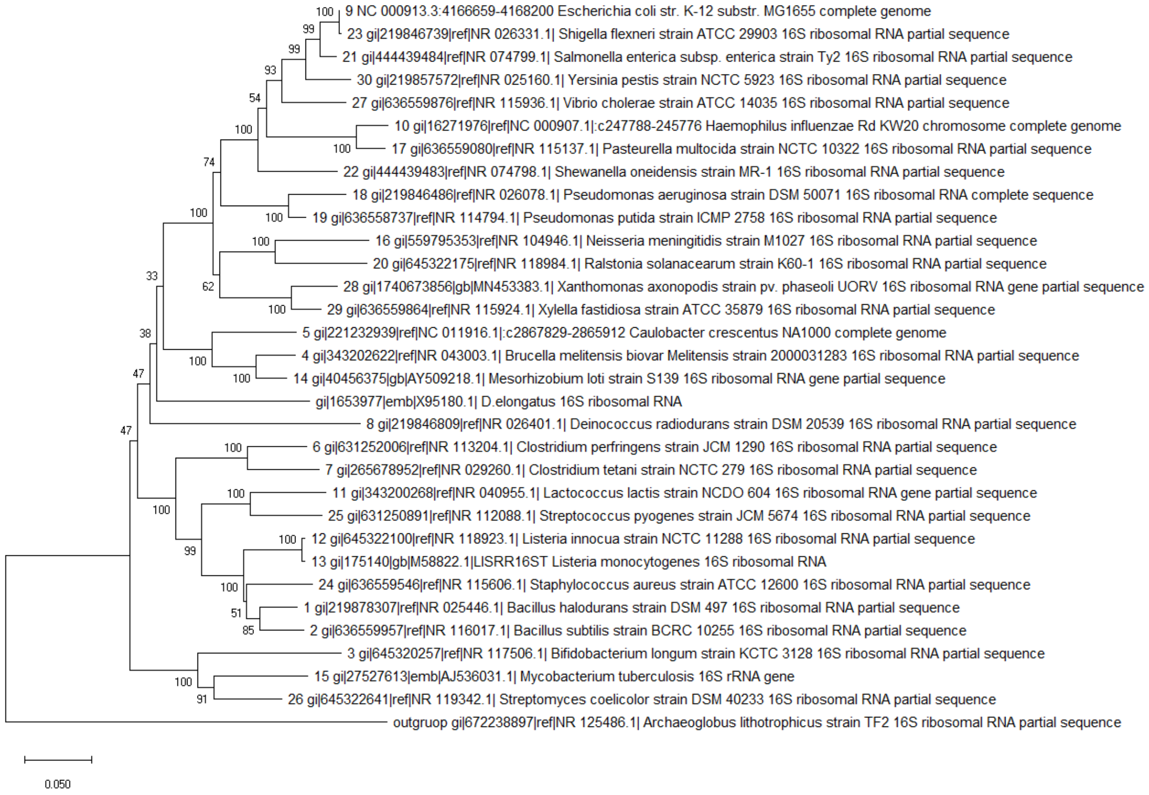

Figure S1.18: Phylogenetic tree of 16S rRNA gene sequences of *Desulfobulbus elongatus* and microbial strains belonging to the training set of PhiSpy.

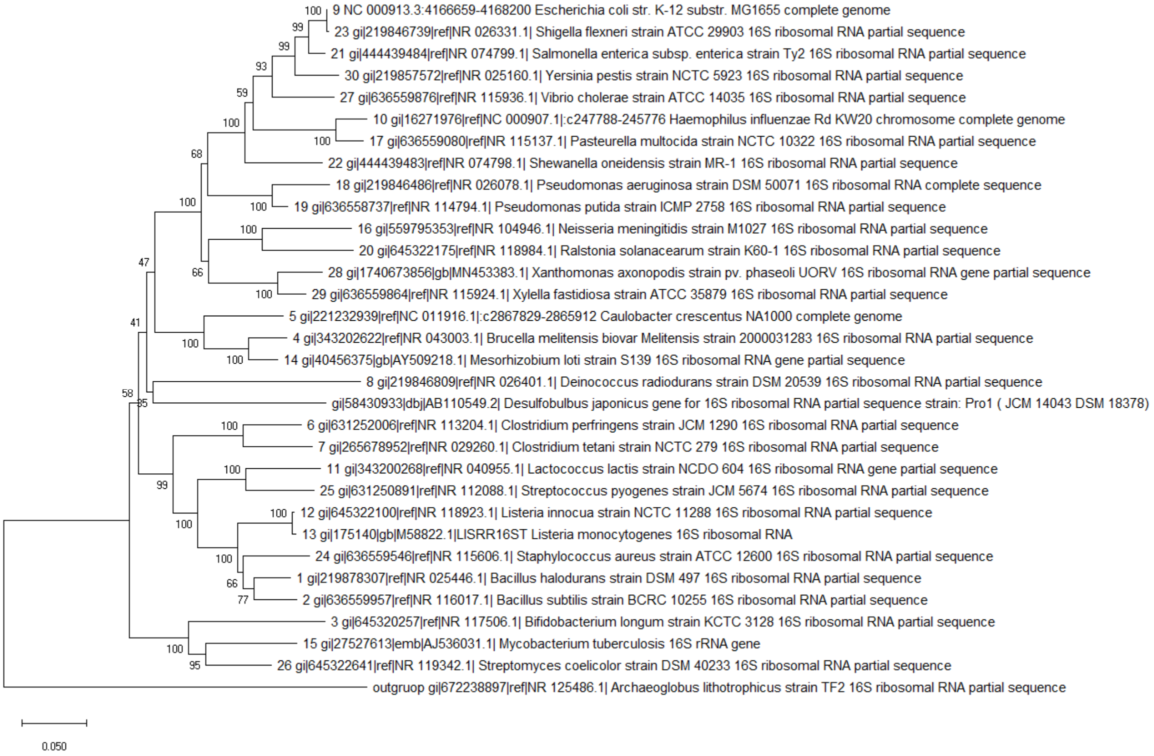

Figure S1.19: Phylogenetic tree of 16S rRNA gene sequences of *Desulfobulbus japonicus* DSM 18378 and microbial strains belonging to the training set of PhiSpy.

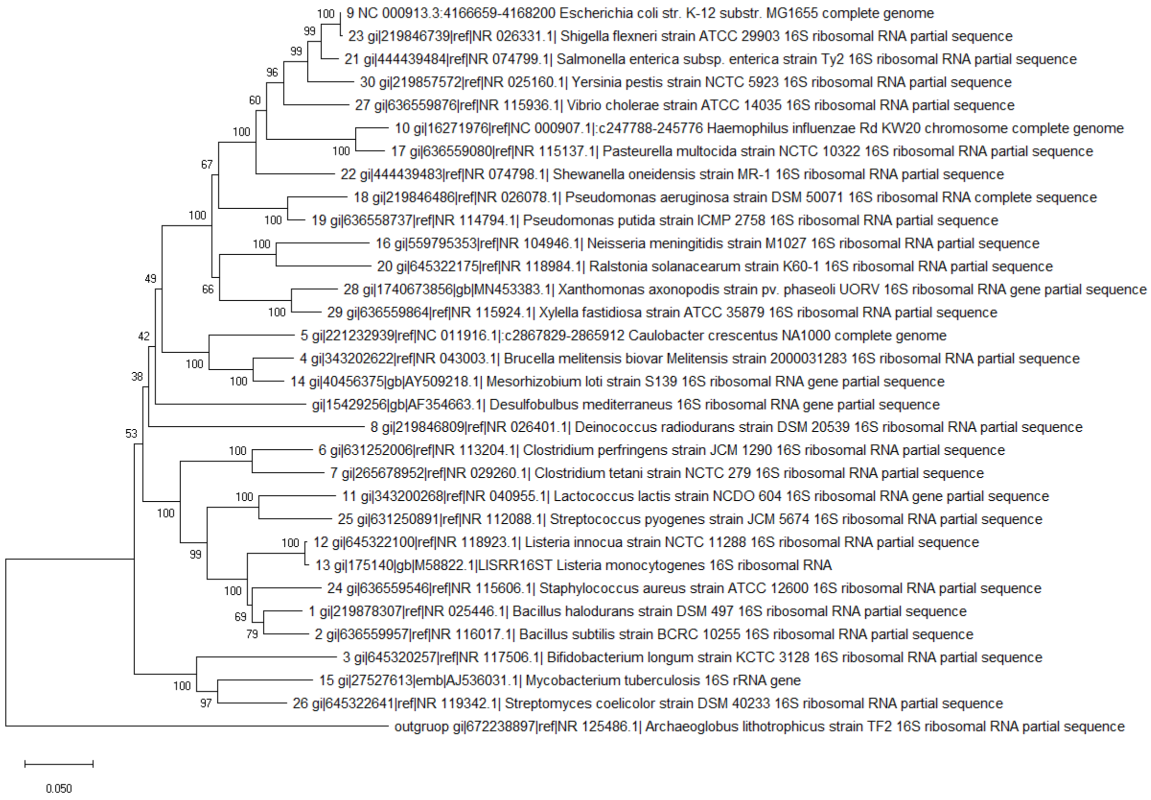

Figure S1.20: Phylogenetic tree of 16S rRNA gene sequences of *Desulfobulbus mediterraneus* and microbial strains belonging to the training set of PhiSpy.

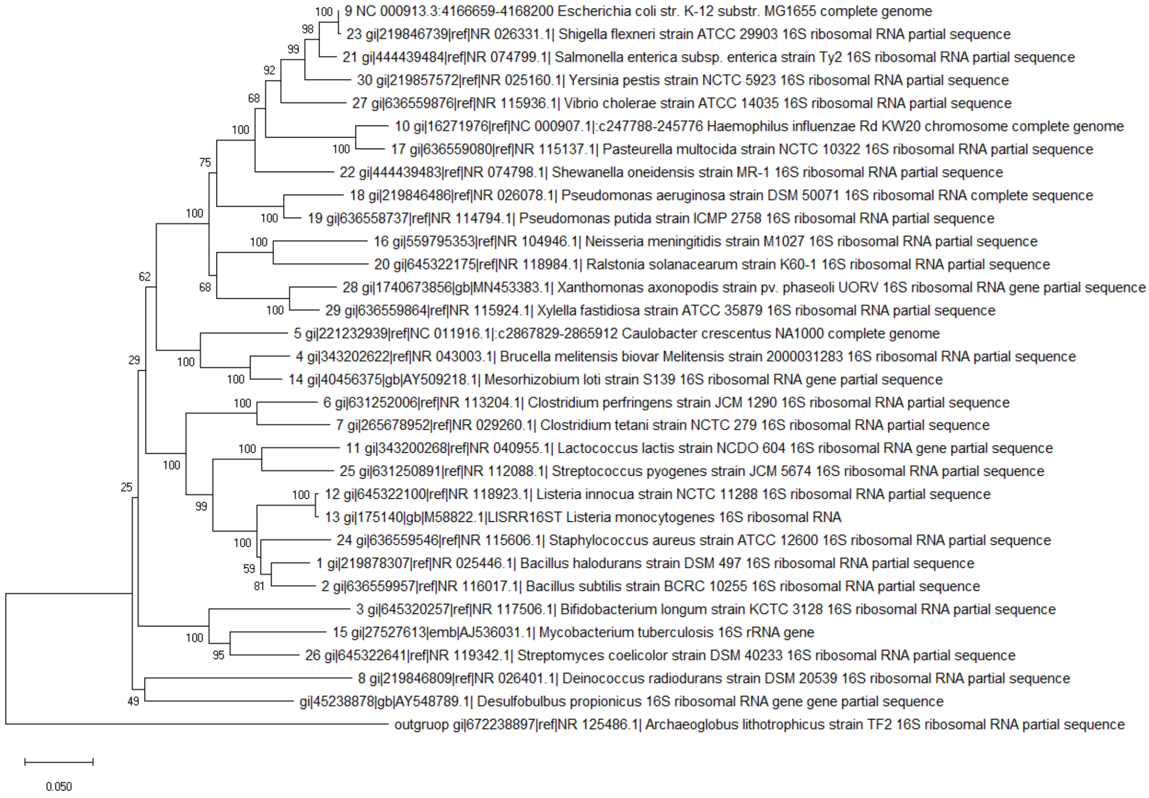

Figure S1.21: Phylogenetic tree of 16S rRNA gene sequences of *Desulfobulbus propionicus* type strain 1pr3 and microbial strains belonging to the training set of PhiSpy.

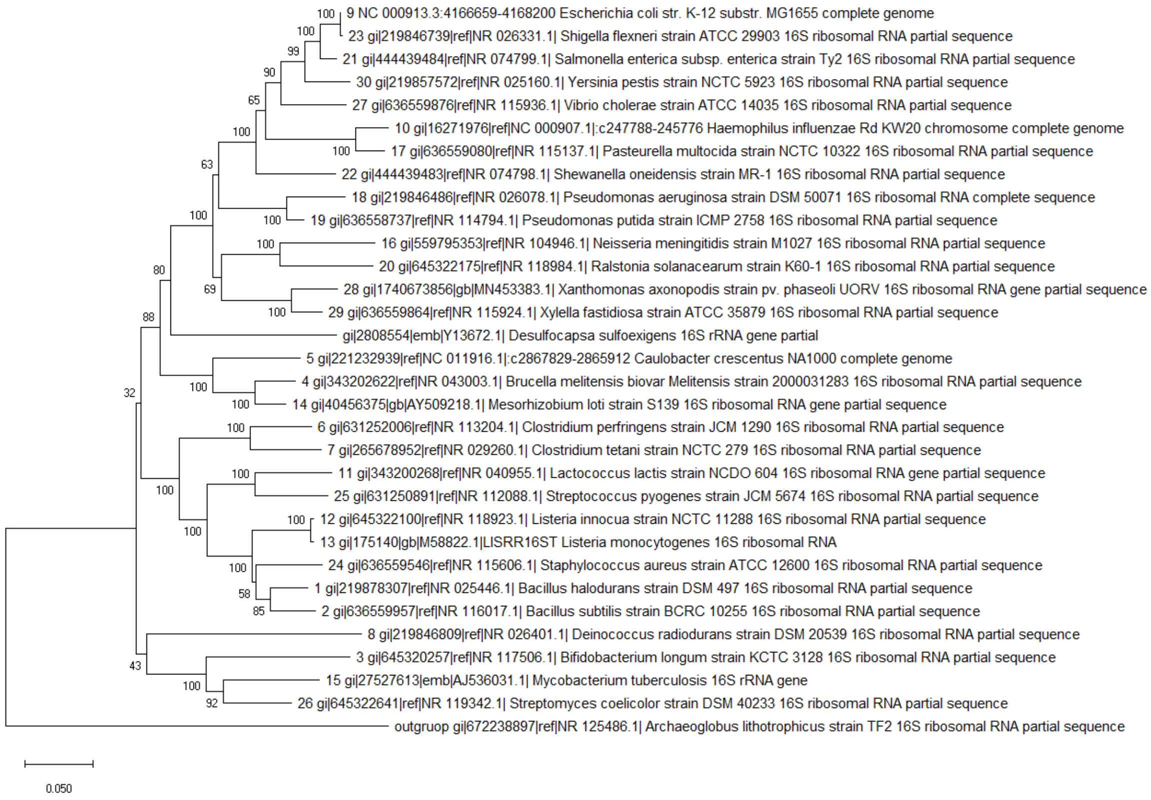

Figure S1.22: Phylogenetic tree of 16S rRNA gene sequences of *Desulfocapsa sulfexigens* DSM 10523 and microbial strains belonging to the training set of PhiSpy.

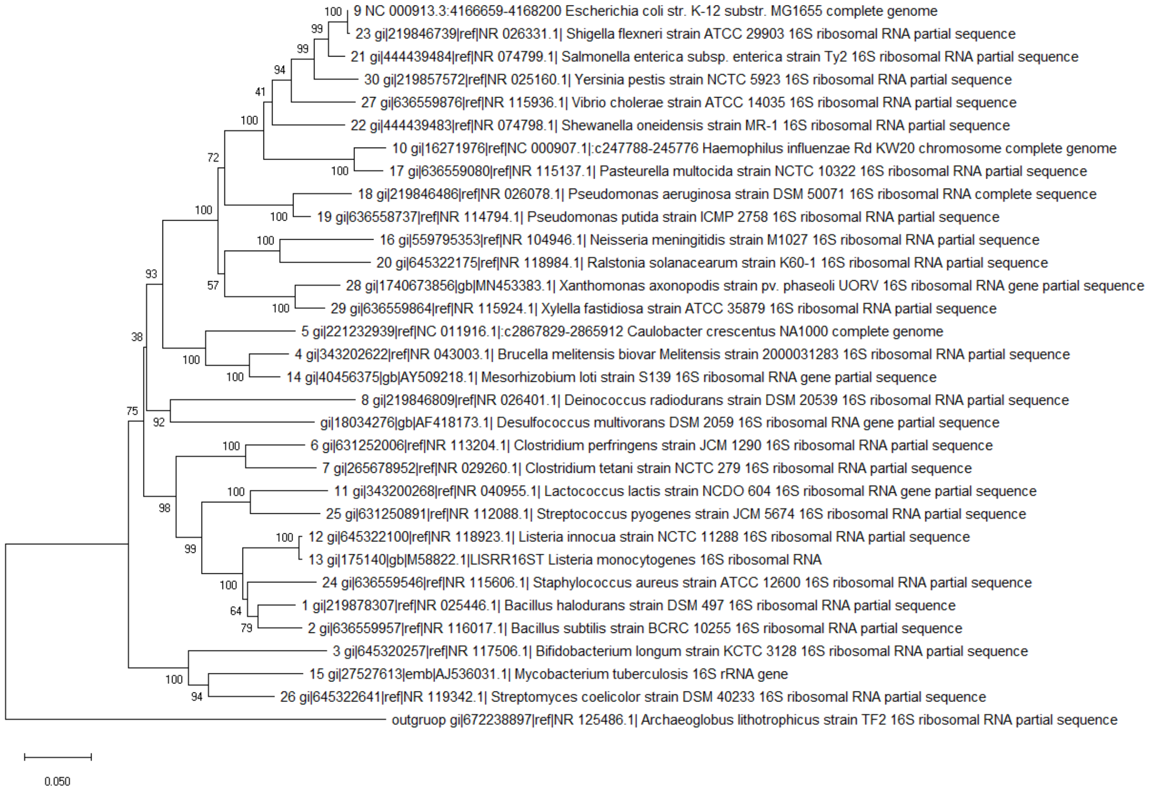

Figure S1.23: Phylogenetic tree of 16S rRNA gene sequences of *Desulfococcus multivorans* DSM 2059 and microbial strains belonging to the training set of PhiSpy.

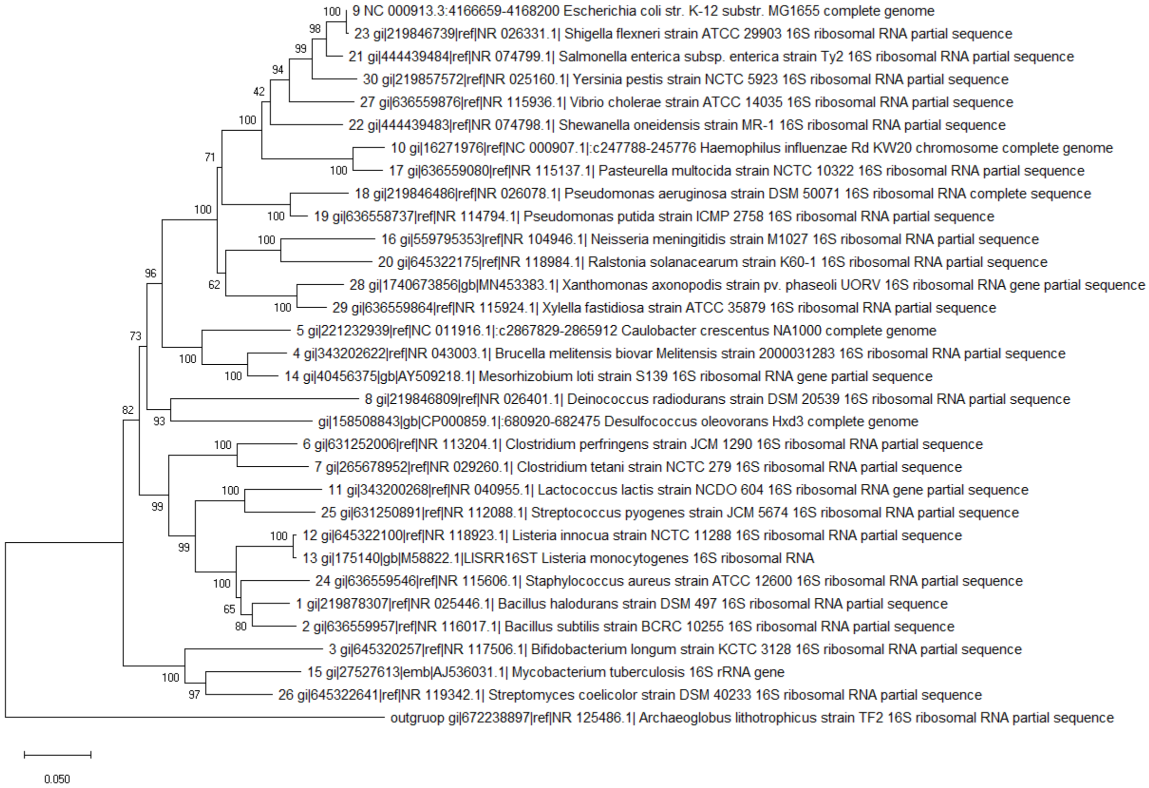

Figure S1.24: Phylogenetic tree of 16S rRNA gene sequences of *Desulfococcus oleovorans* Hxd3 and microbial strains belonging to the training set of PhiSpy.

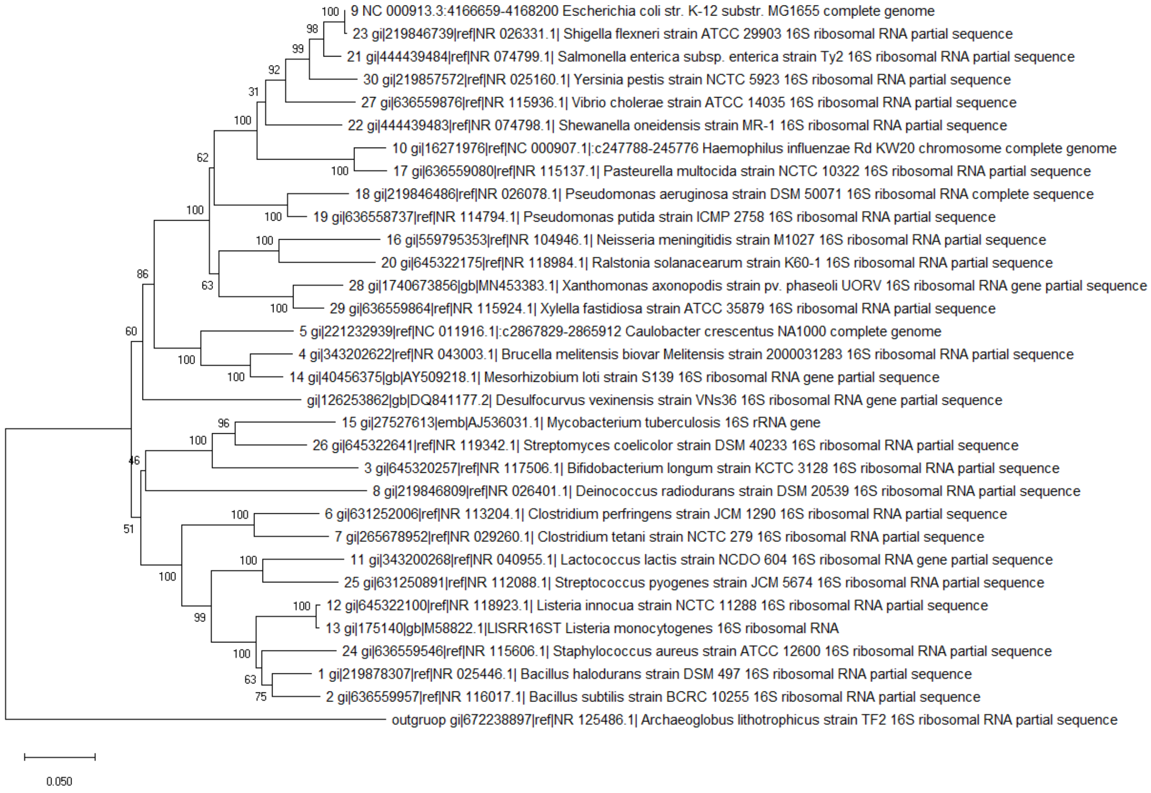

Figure S1.25: Phylogenetic tree of 16S rRNA gene sequences of *Desulfocurvus vexinensis* and microbial strains belonging to the training set of PhiSpy.

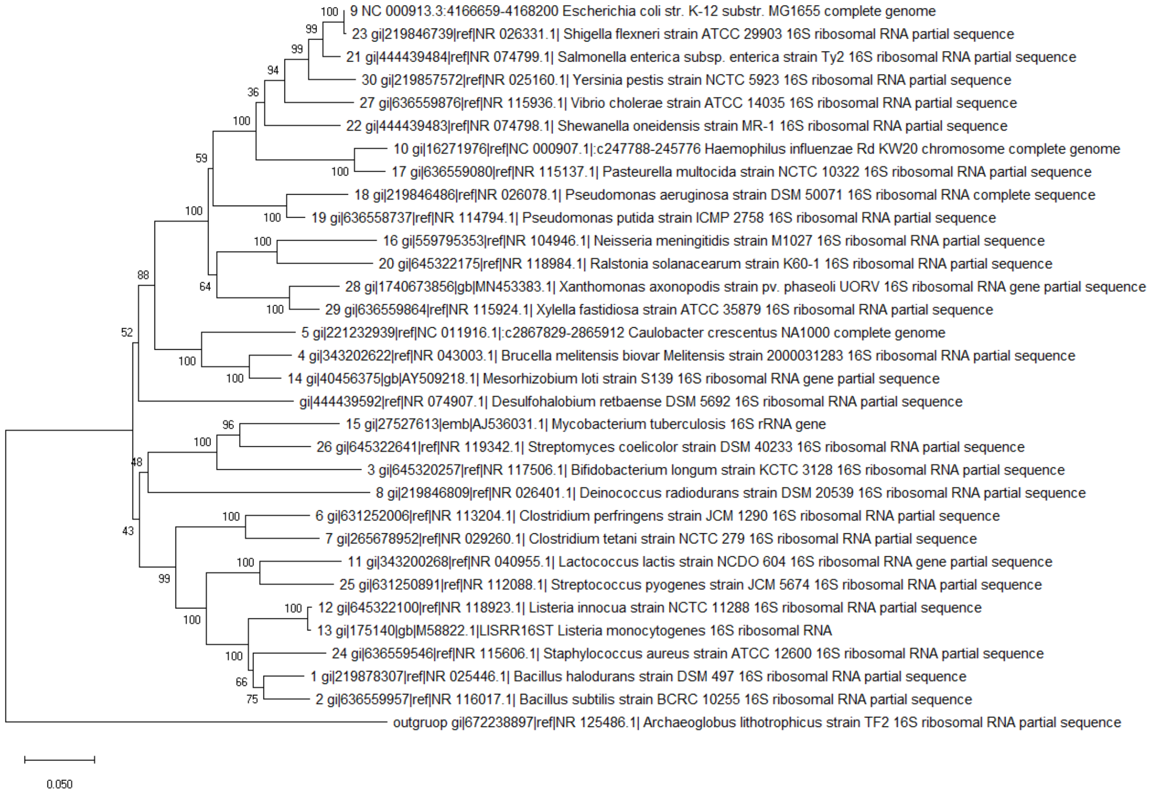

Figure S1.26: Phylogenetic tree of 16S rRNA gene sequences of *Desulfohalobium retbaense* DSM 5692 and microbial strains belonging to the training set of PhiSpy.

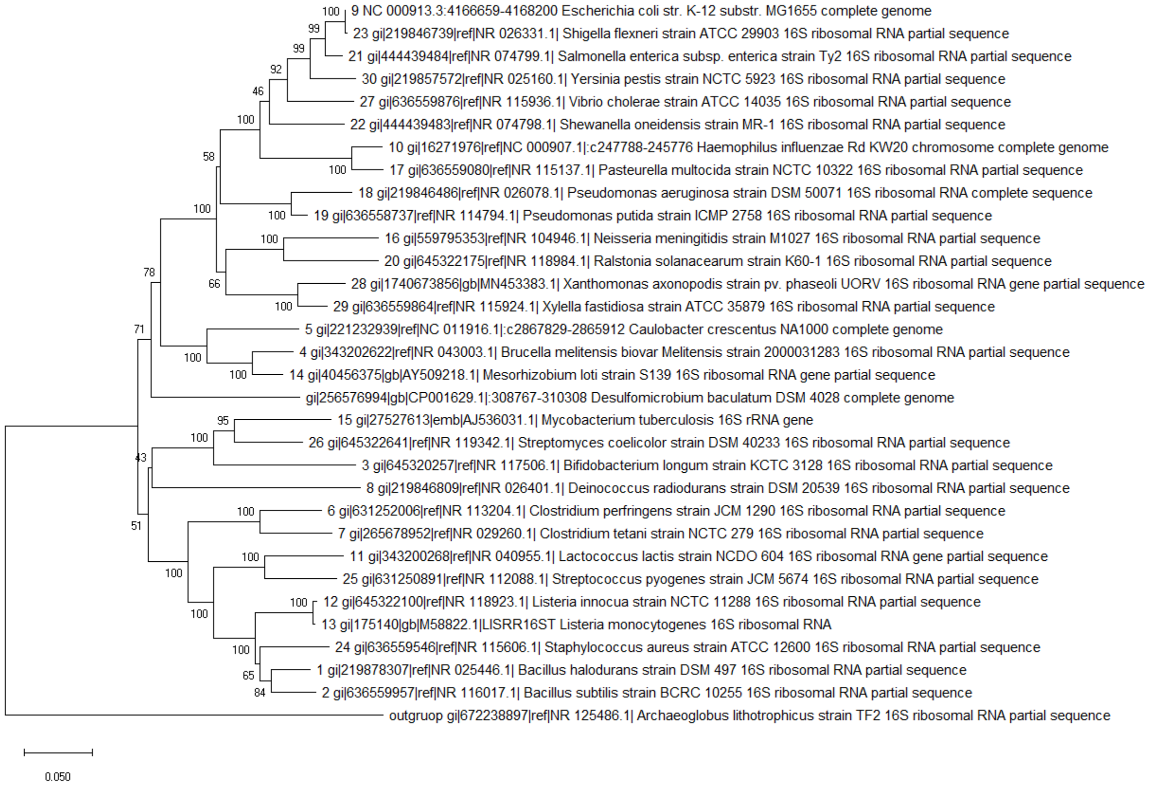

Figure S1.27: Phylogenetic tree of 16S rRNA gene sequences of *Desulfomicrobium baculatum* DSM 4028 and microbial strains belonging to the training set of PhiSpy.

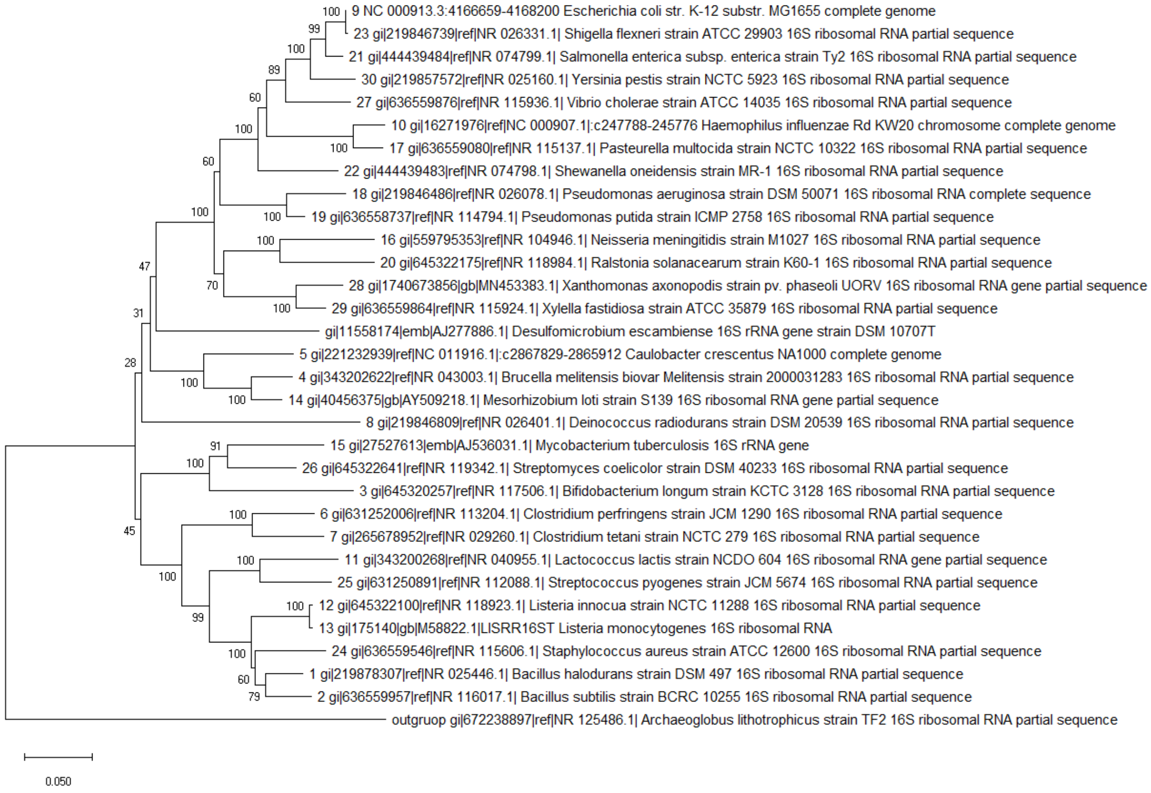

Figure S1.28: Phylogenetic tree of 16S rRNA gene sequences of *Desulfomicrobium escambiense* and microbial strains belonging to the training set of PhiSpy.

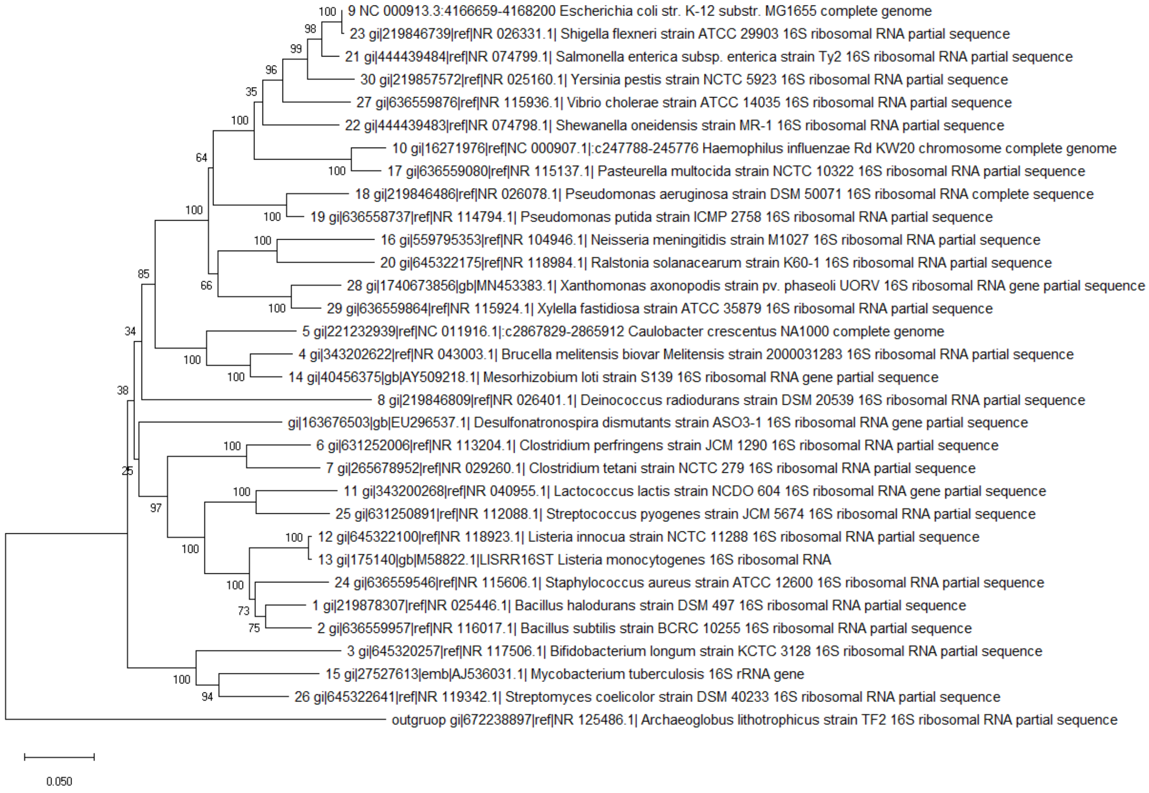

Figure S1.29: Phylogenetic tree of 16S rRNA gene sequences of *Desulfonatronospira dismutants* strain ASO3-1 and microbial strains belonging to the training set of PhiSpy.

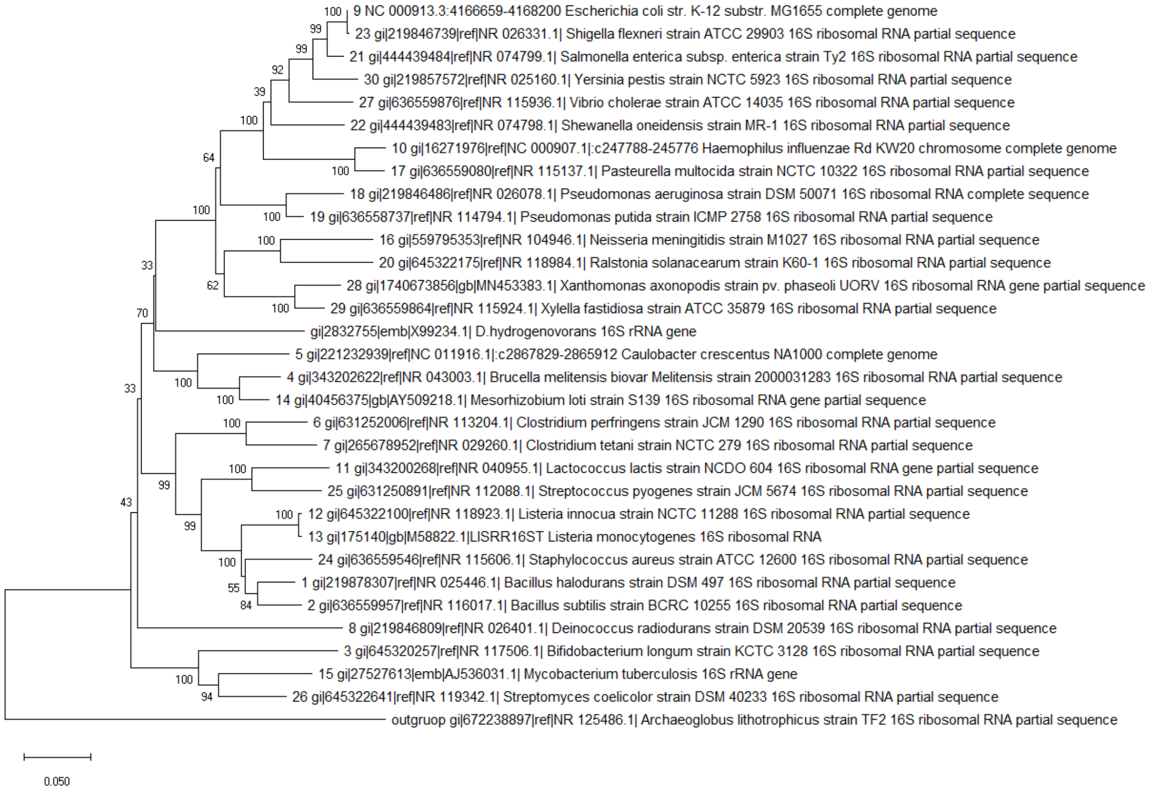

Figure S1.30: Phylogenetic tree of 16S rRNA gene sequences of *Desulfonatronovibrio hydrogenovorans* DSM 9292 and microbial strains belonging to the training set of PhiSpy.

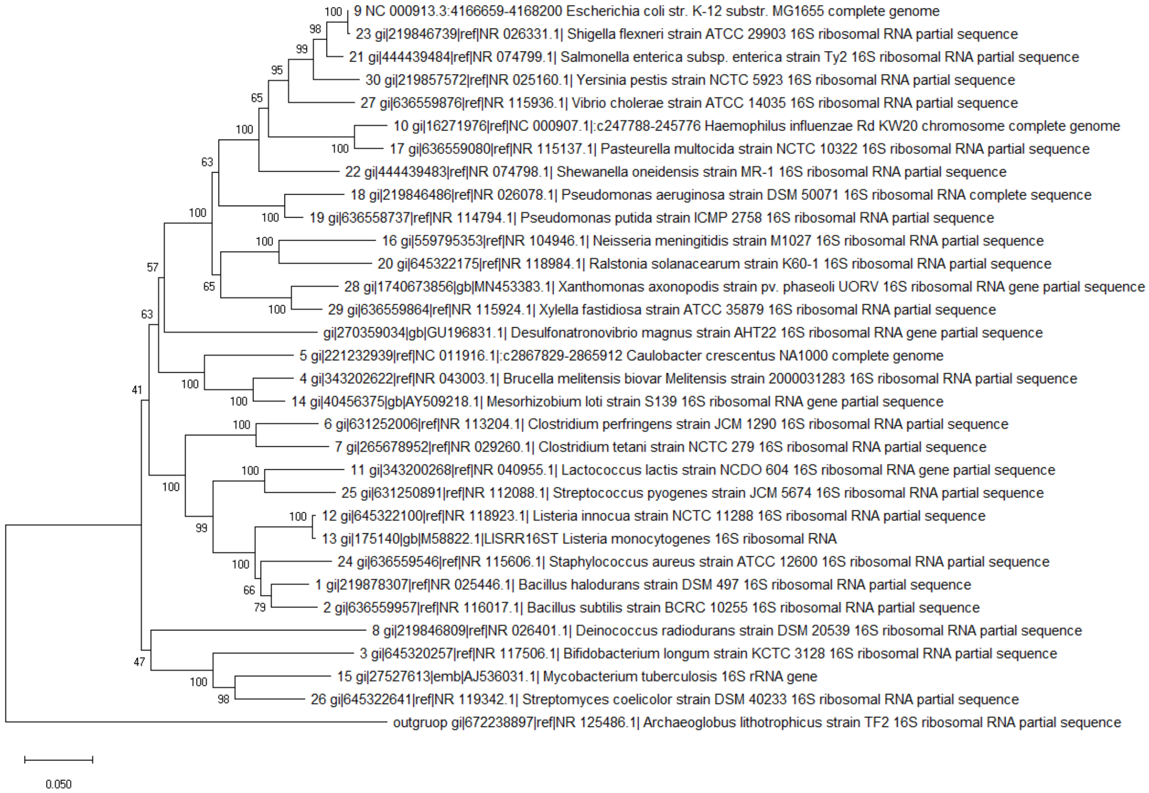

Figure S1.31: Phylogenetic tree of 16S rRNA gene sequences of *Desulfonatronovibrio magnus* strain AHT22 and microbial strains belonging to the training set of PhiSpy.

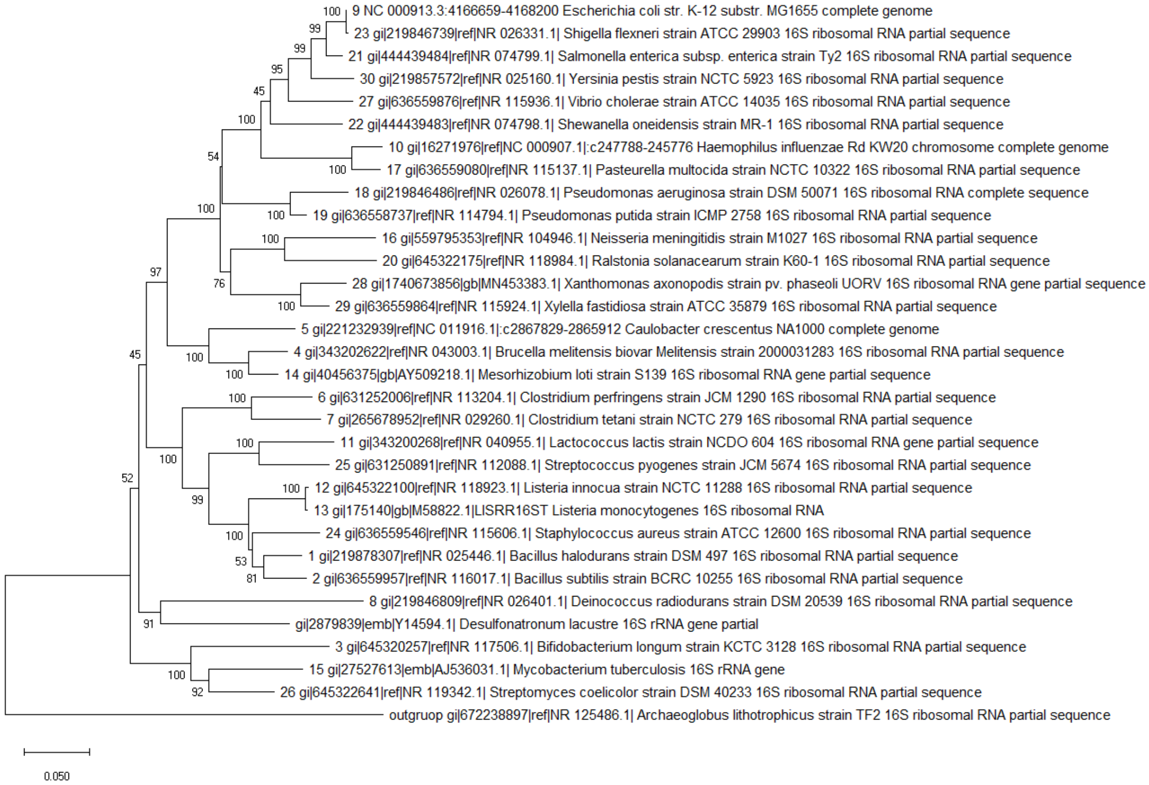

Figure S1.32: Phylogenetic tree of 16S rRNA gene sequences of *Desulfonatronum lacustre* and microbial strains belonging to the training set of PhiSpy.

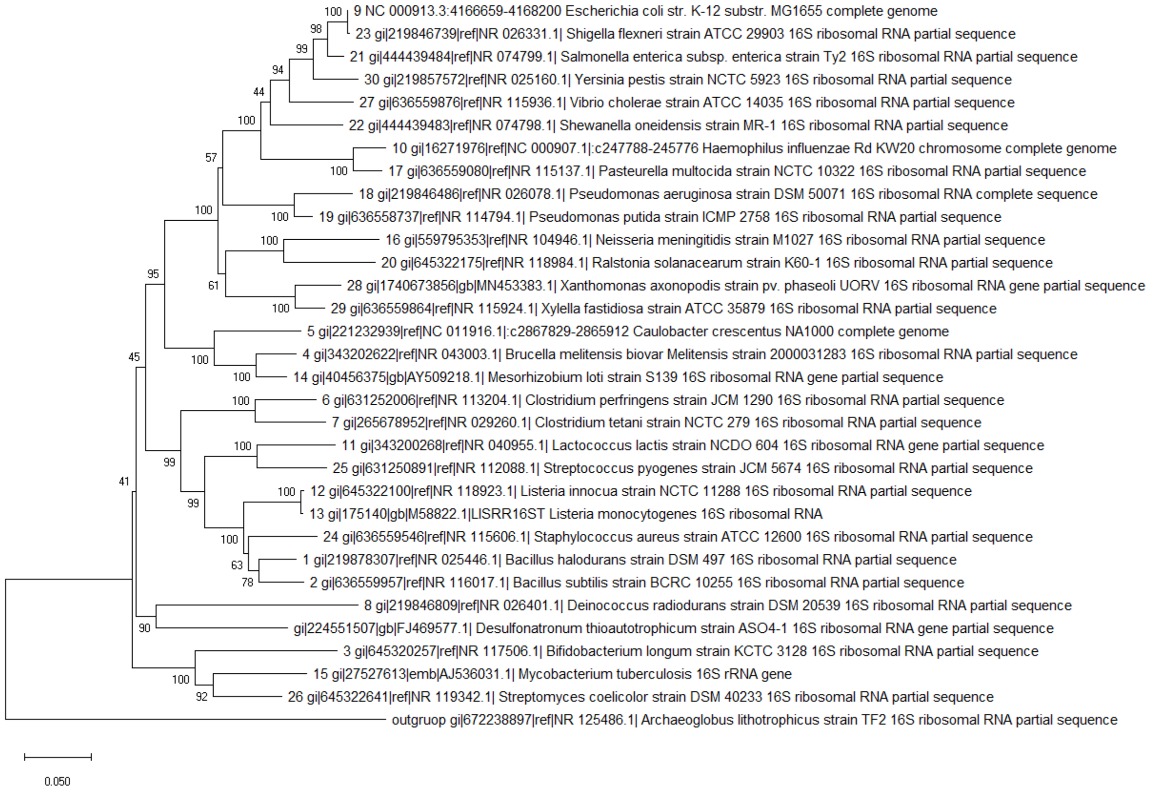

Figure S1.33: Phylogenetic tree of 16S rRNA gene sequences of *Desulfonatronum thioautotrophicum* strain ASO4-1 and microbial strains belonging to the training set of PhiSpy.

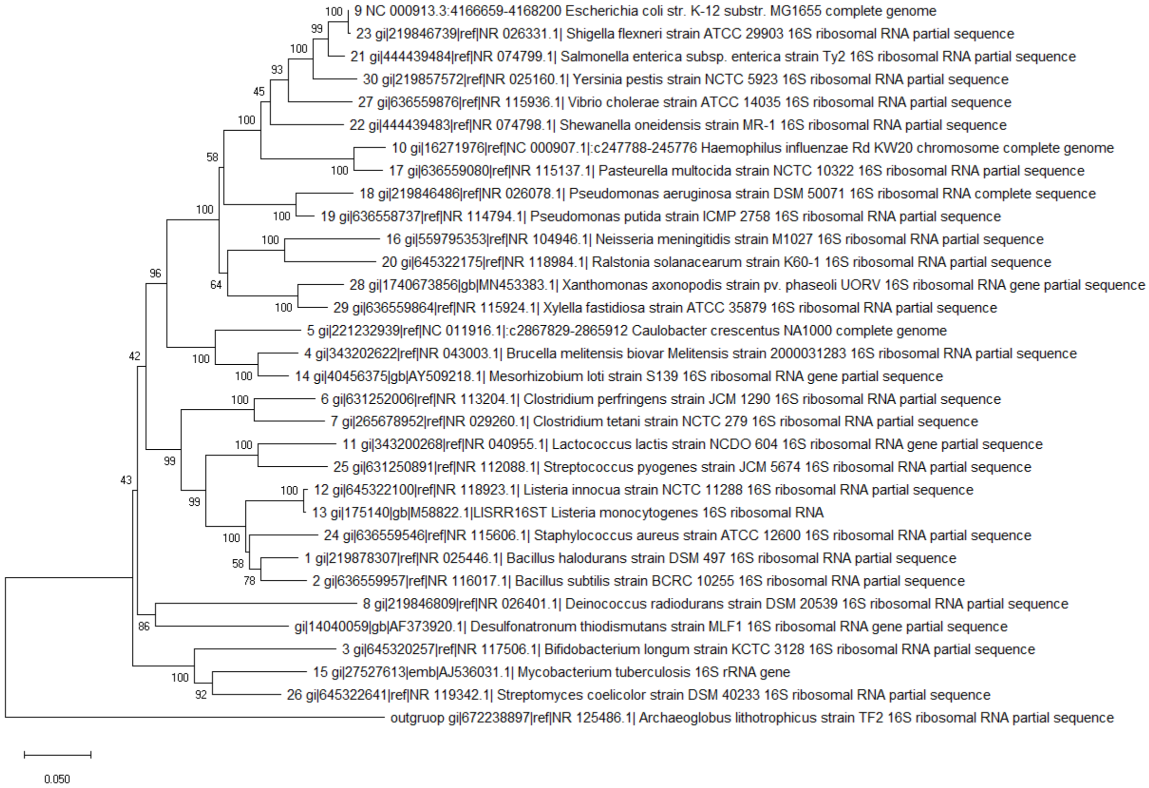

Figure S1.34: Phylogenetic tree of 16S rRNA gene sequences of *Desulfonatronum thiodismutans* and microbial strains belonging to the training set of PhiSpy.

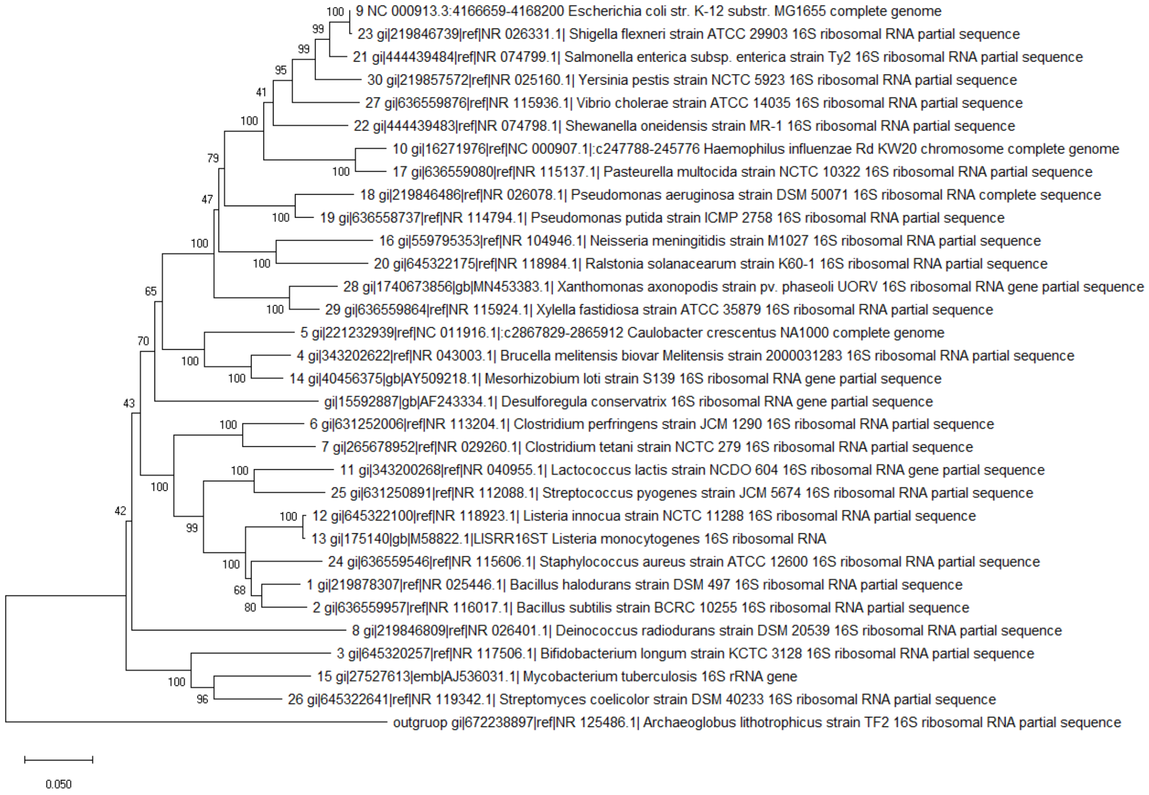

Figure S1.35: Phylogenetic tree of 16S rRNA gene sequences of *Desulforegula conservatrix* and microbial strains belonging to the training set of PhiSpy.

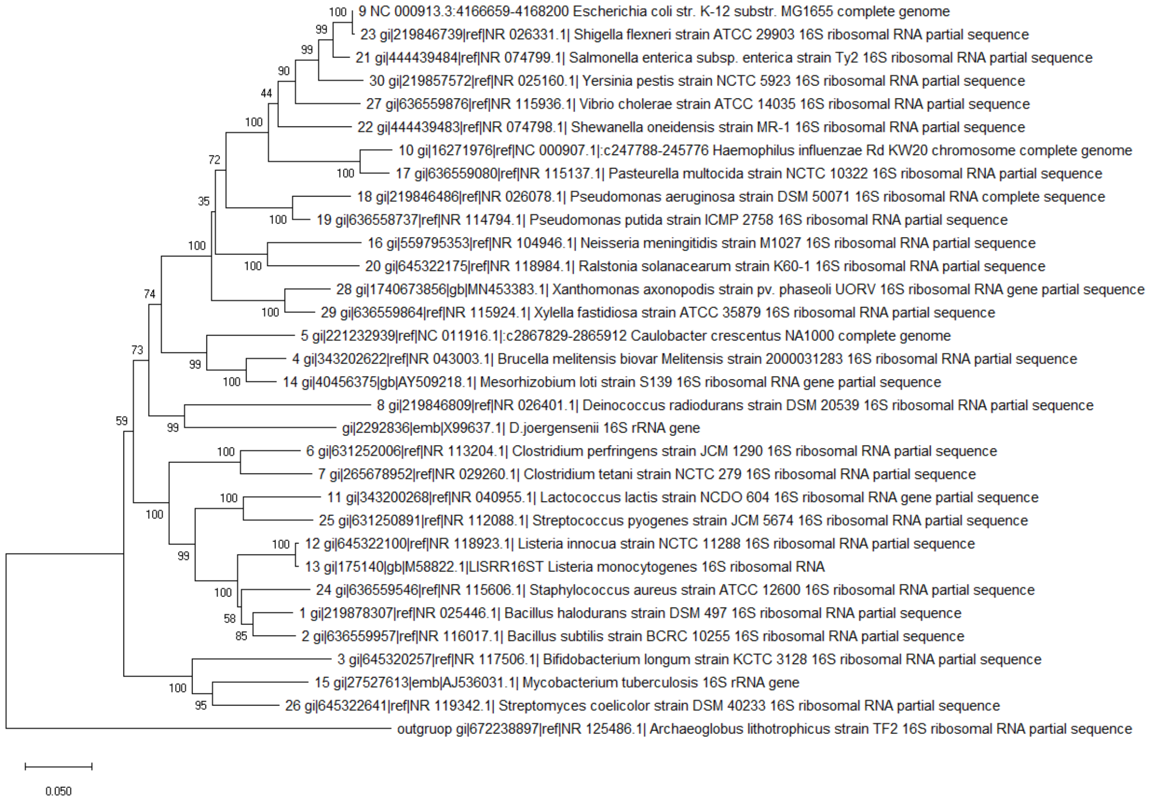

Figure S1.36: Phylogenetic tree of 16S rRNA gene sequences of *Desulfospira joergensenii* DSM 10085 and microbial strains belonging to the training set of PhiSpy.

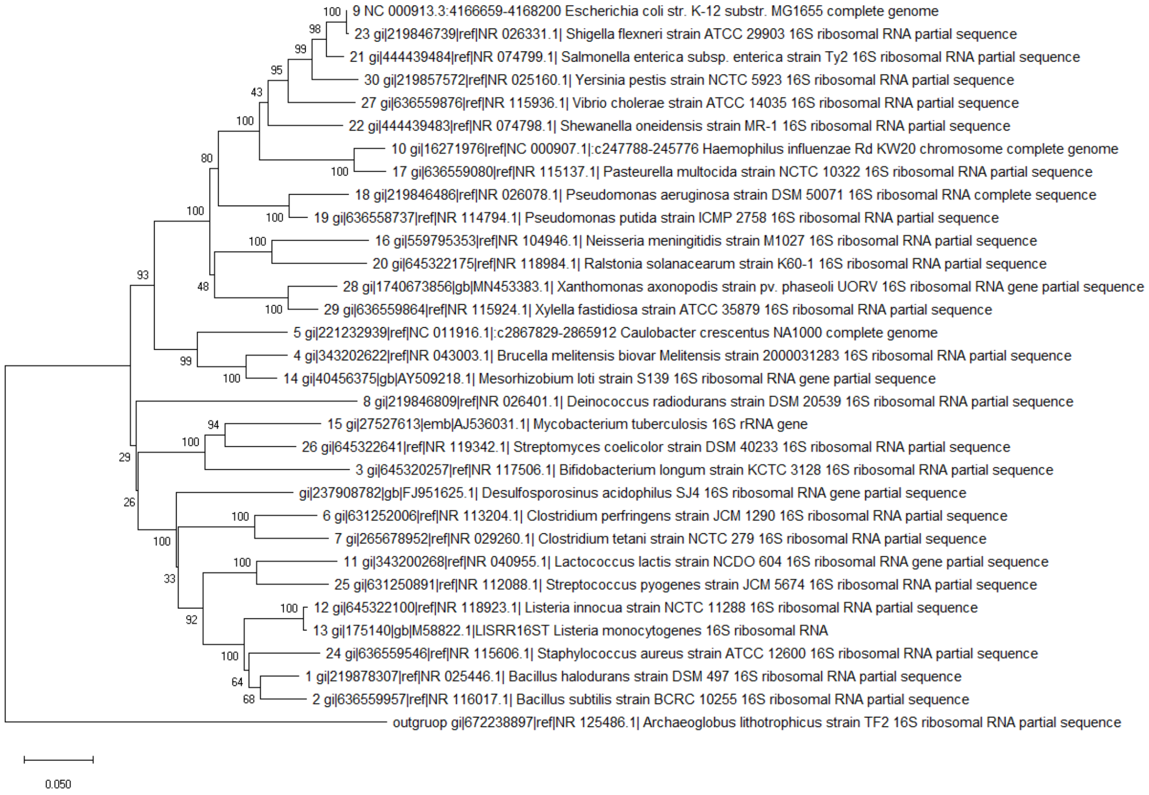

Figure S1.37: Phylogenetic tree of 16S rRNA gene sequences of *Desulfosporosinus acidiphilus* SJ4 and microbial strains belonging to the training set of PhiSpy.

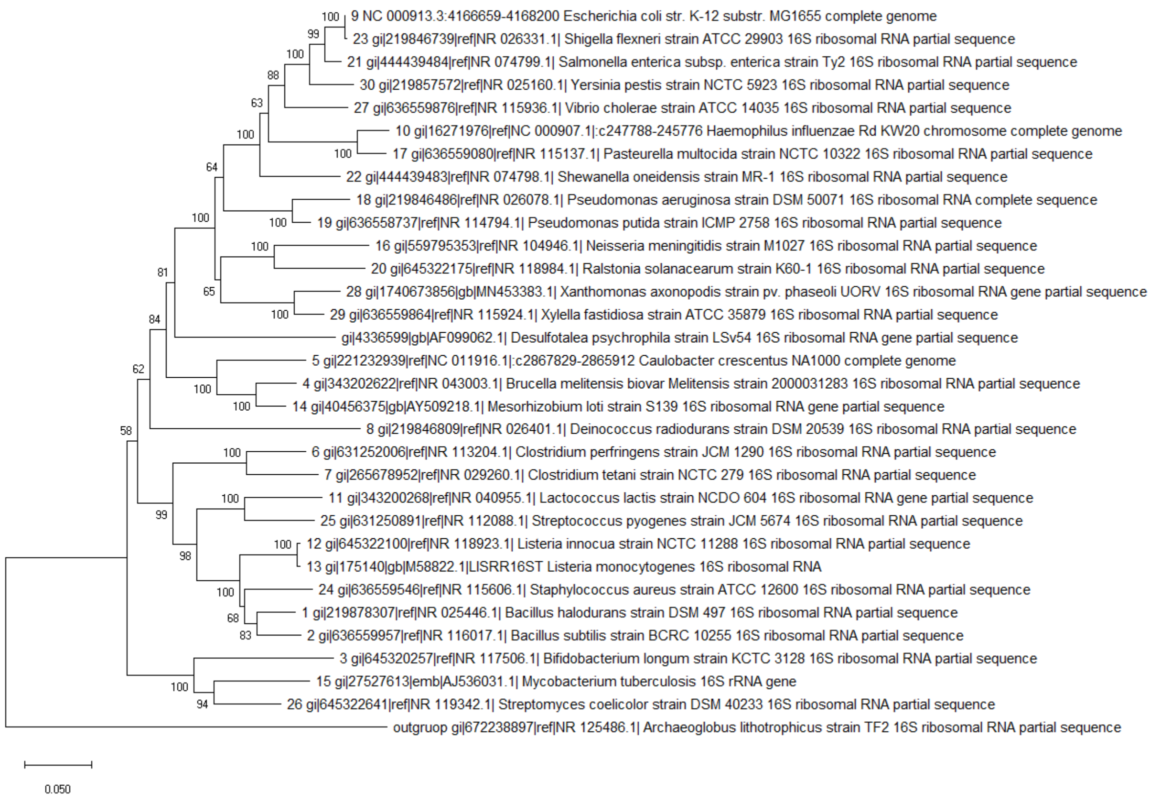

Figure S1.38: Phylogenetic tree of 16S rRNA gene sequences of *Desulfotalea psychrophila* strain LSv54 and microbial strains belonging to the training set of PhiSpy.

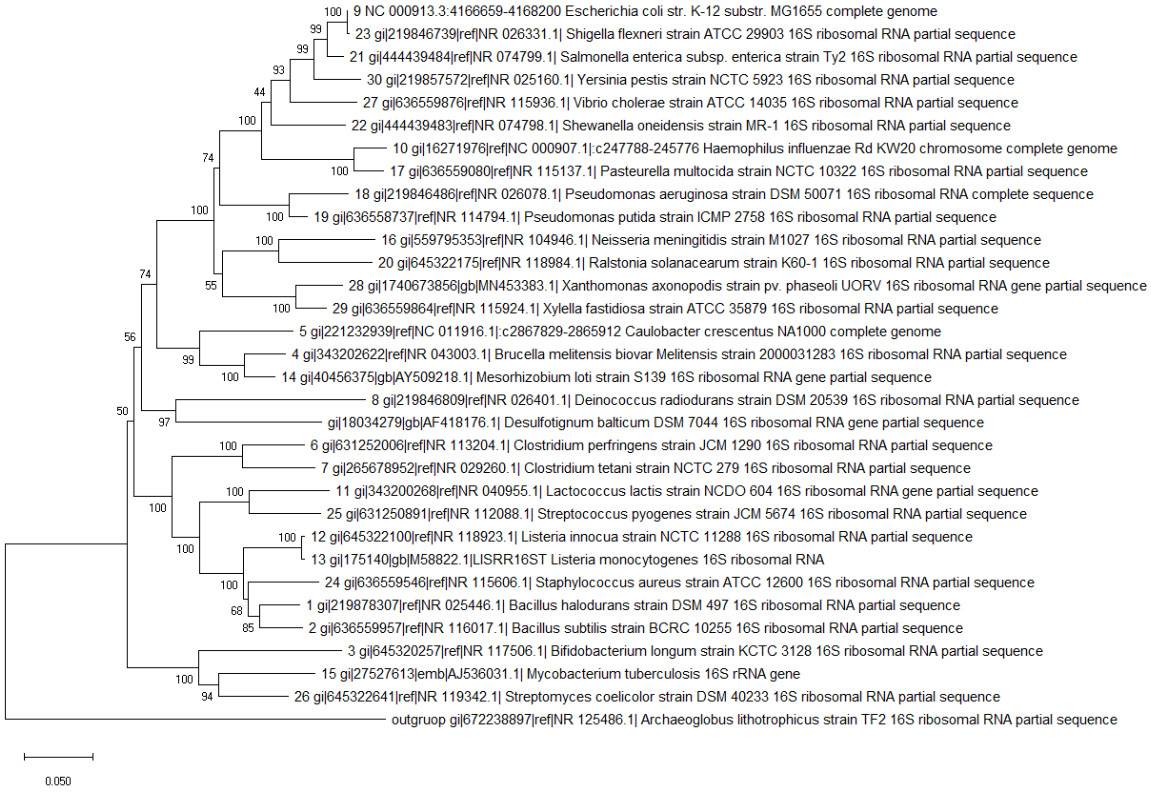

Figure S1.39: Phylogenetic tree of 16S rRNA gene sequences of *Desulfotignum balticum* DSM 7044 and microbial strains belonging to the training set of PhiSpy.

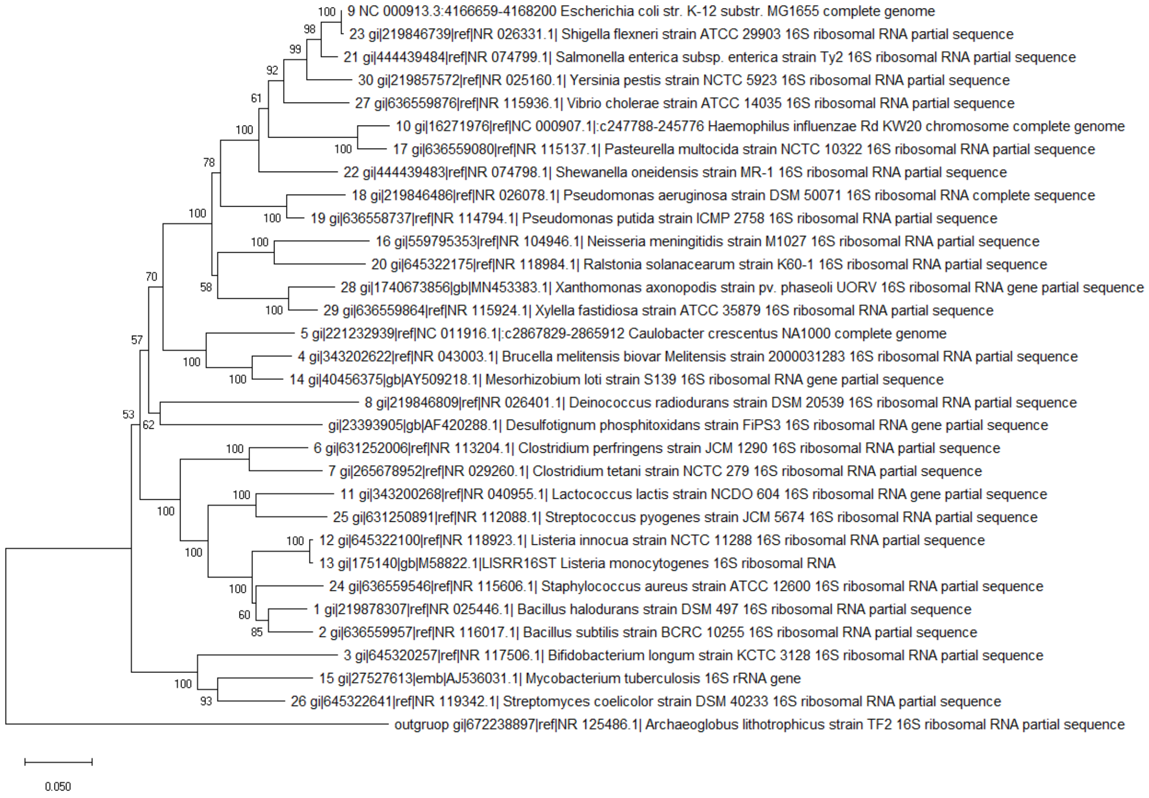

Figure S1.40: Phylogenetic tree of 16S rRNA gene sequences of *Desulfotignum phosphitoxidans* DSM 13687 and microbial strains belonging to the training set of PhiSpy.

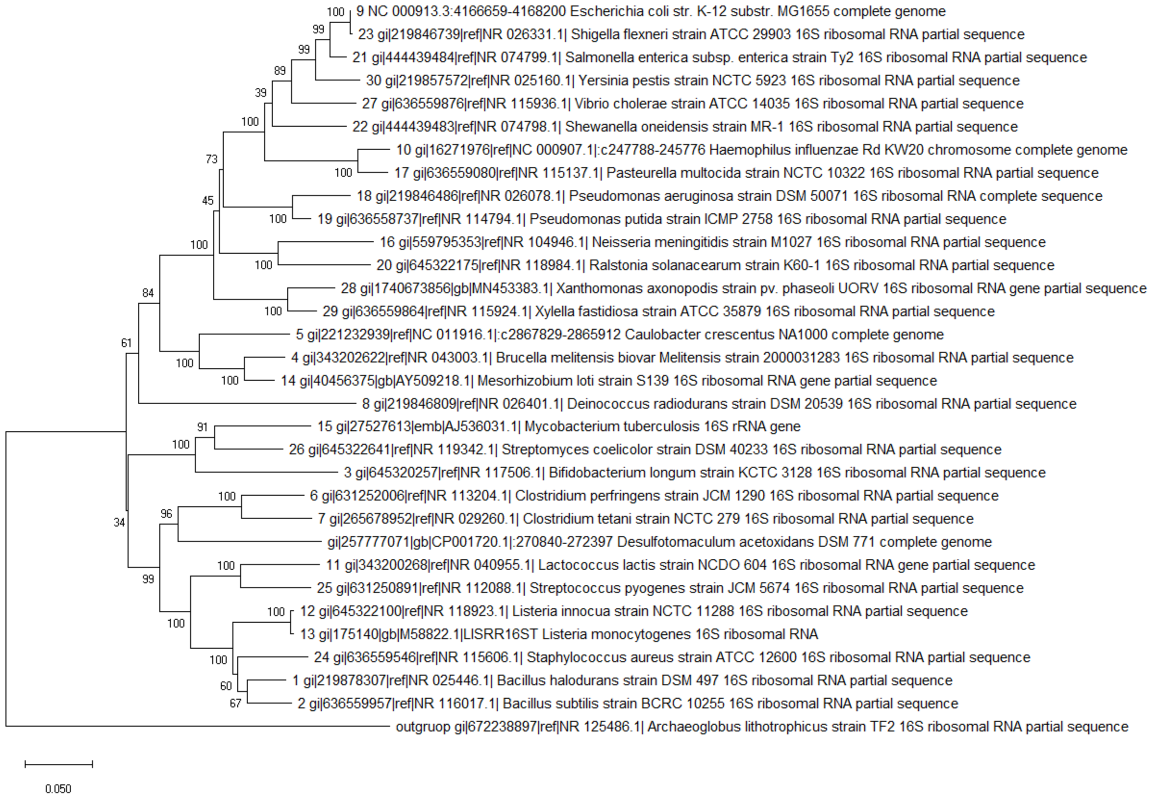

Figure S1.41: Phylogenetic tree of 16S rRNA gene sequences of *Desulfotomaculum acetoxidans* DSM 771 and microbial strains belonging to the training set of PhiSpy.

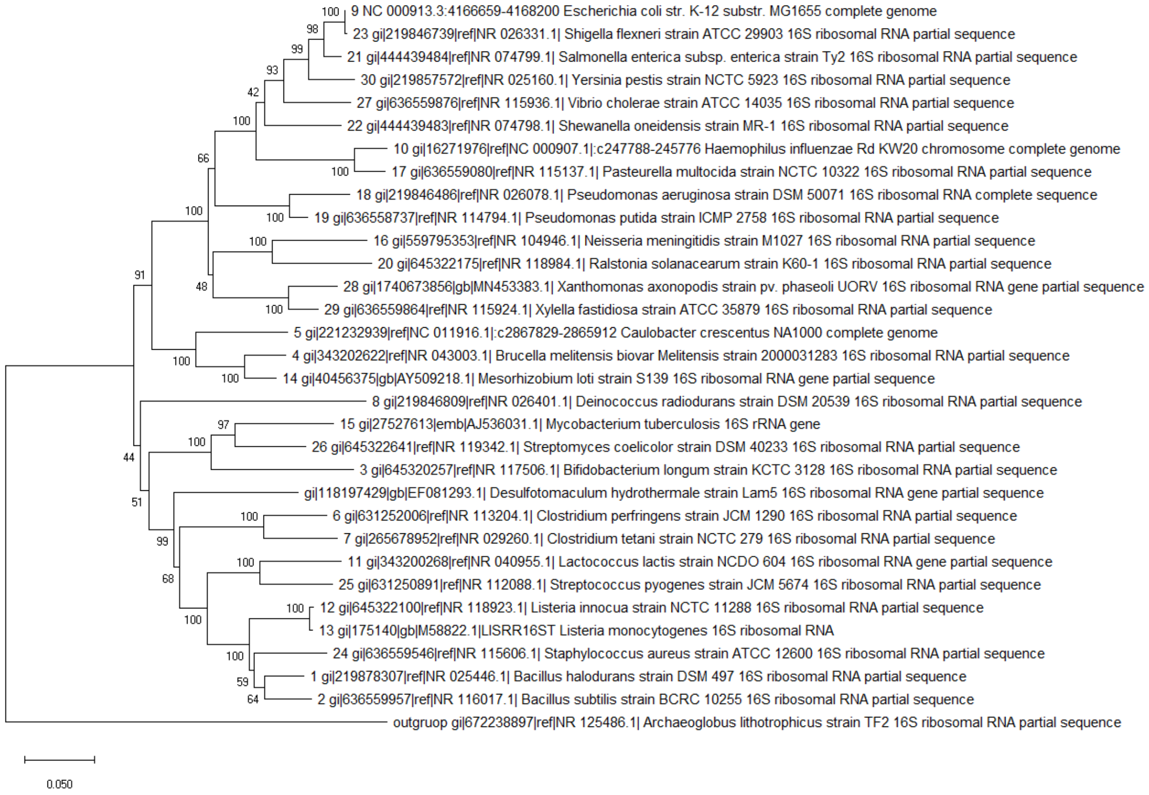

Figure S1.42: Phylogenetic tree of 16S rRNA gene sequences of *Desulfotomaculum hydrothermale* strain Lam5 and microbial strains belonging to the training set of PhiSpy.

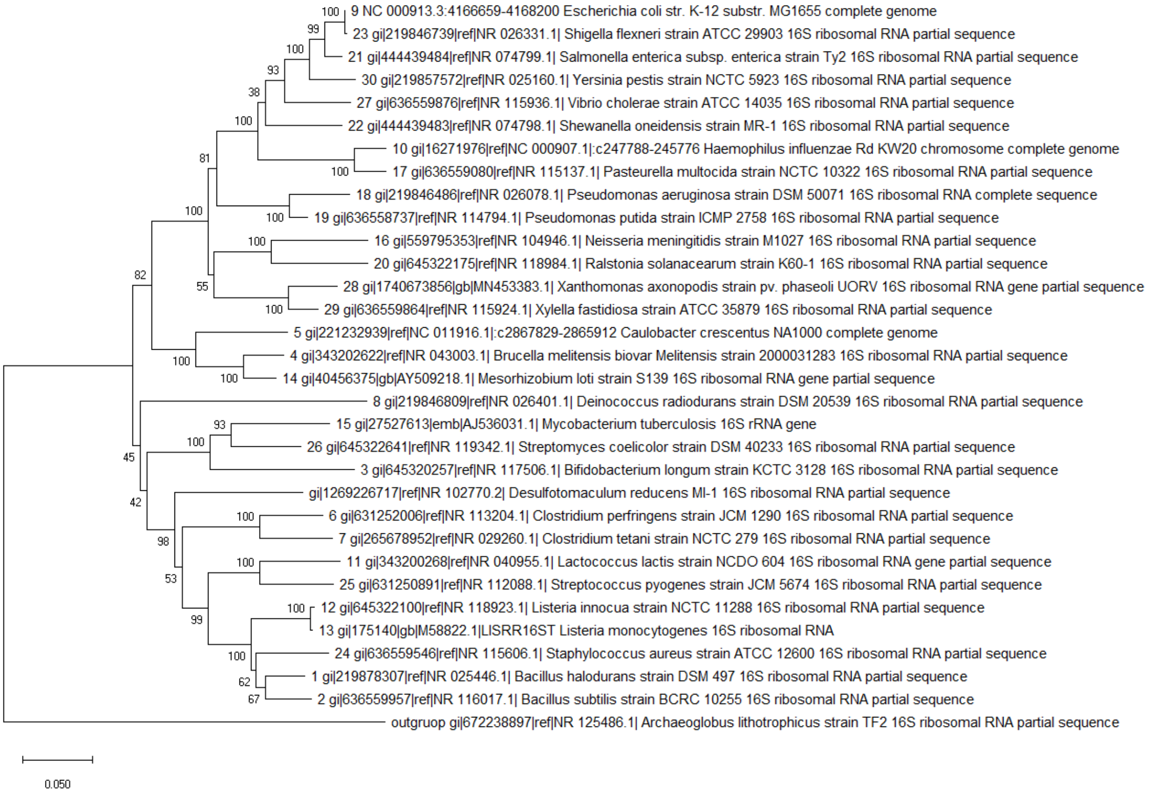

Figure S1.43: Phylogenetic tree of 16S rRNA gene sequences of *Desulfotomaculum reducens* MI-1 and microbial strains belonging to the training set of PhiSpy.

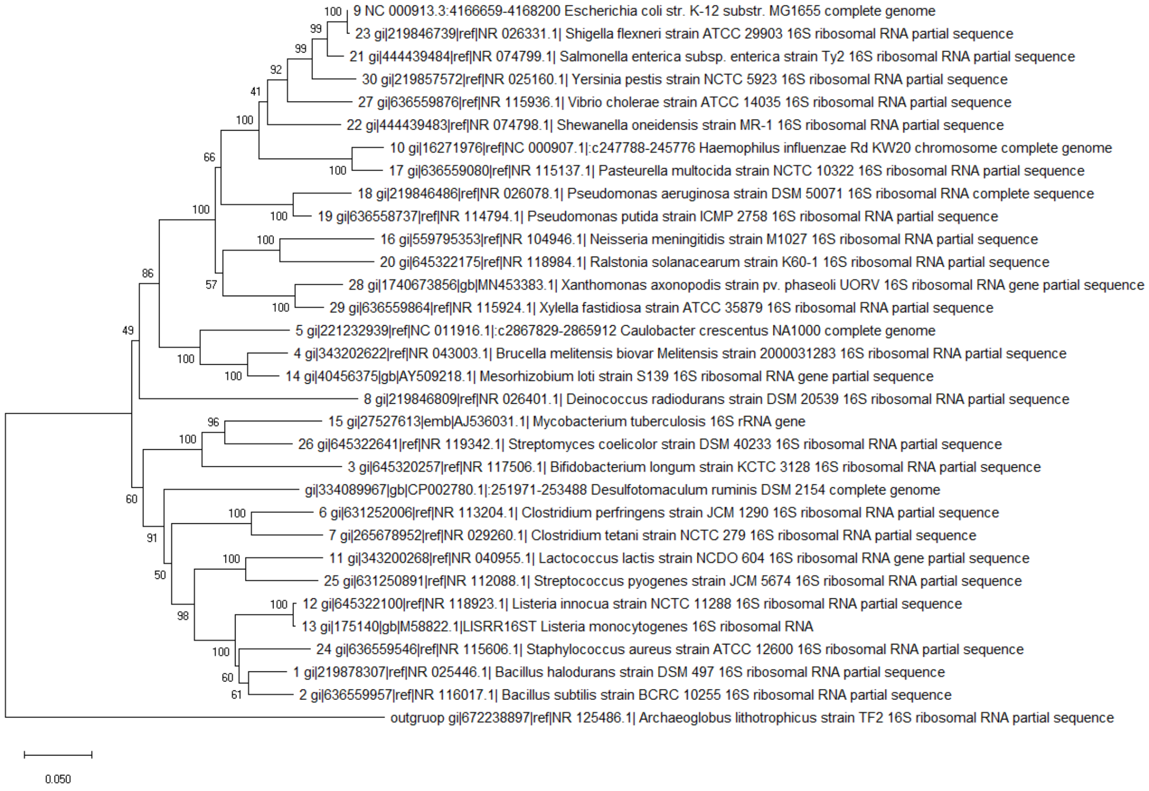

Figure S1.44: Phylogenetic tree of 16S rRNA gene sequences of *Desulfotomaculum ruminis* DSM 2154 and microbial strains belonging to the training set of PhiSpy.

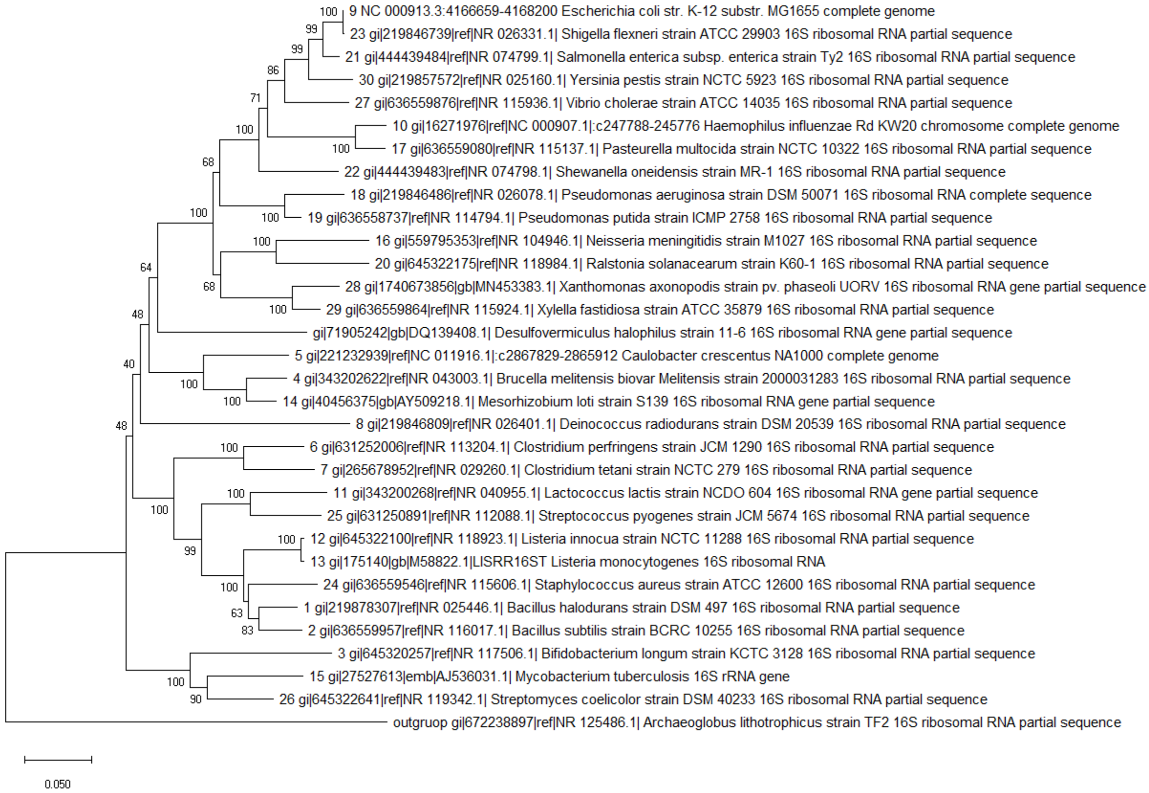

Figure S1.45: Phylogenetic tree of 16S rRNA gene sequences of *Desulfovermiculus halophilus* and microbial strains belonging to the training set of PhiSpy.

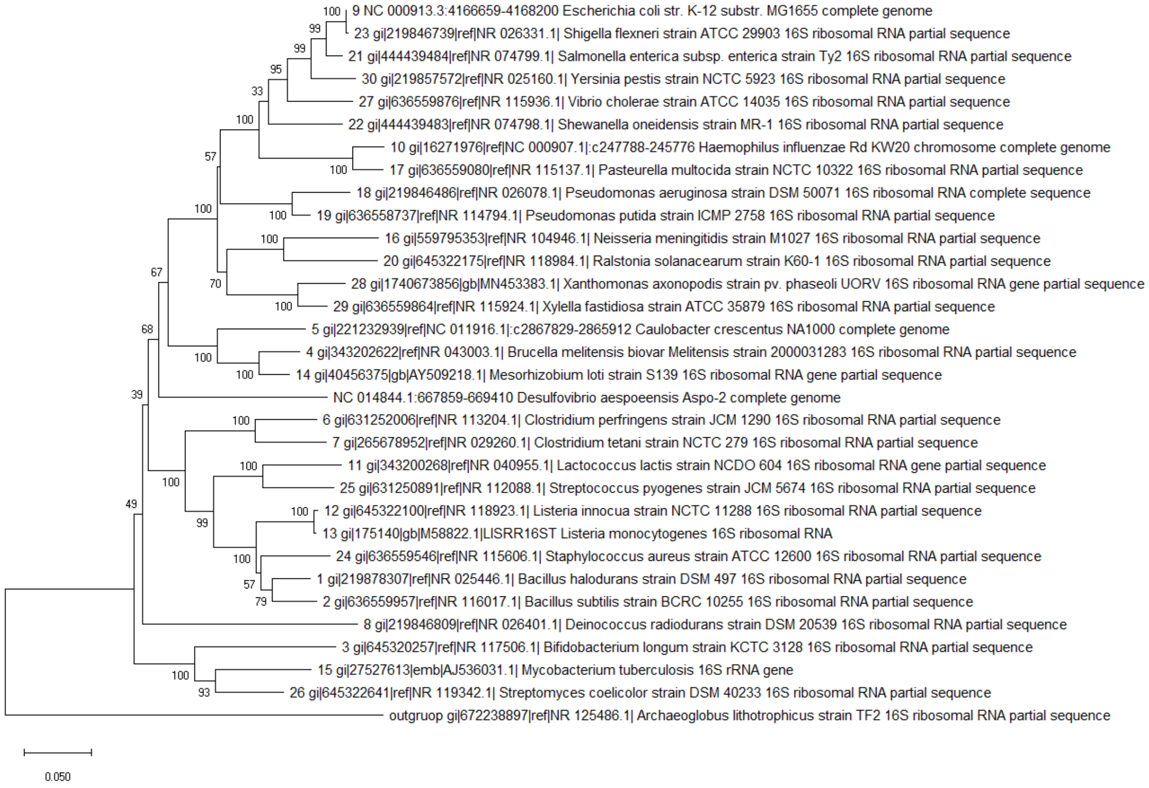

Figure S1.46: Phylogenetic tree of 16S rRNA gene sequences of *Desulfovibrio aespoeensis* Aspo-2 and microbial strains belonging to the training set of PhiSpy.

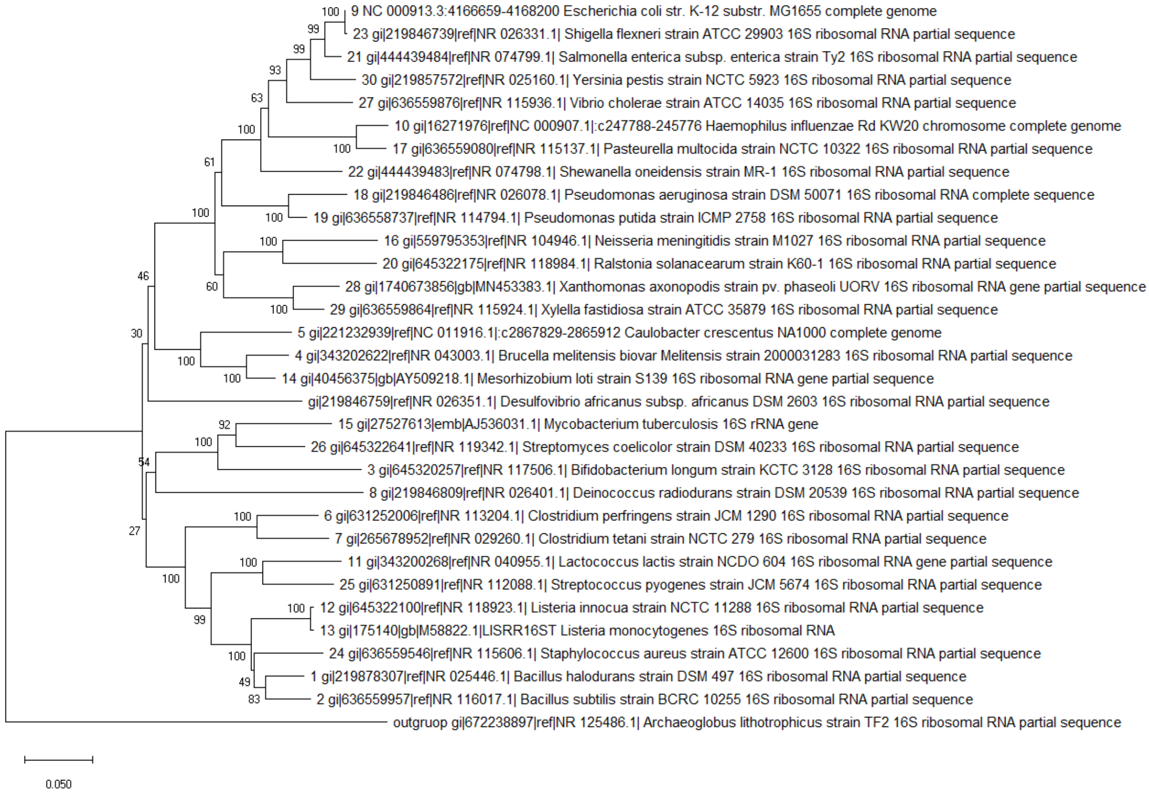

Figure S1.47: Phylogenetic tree of 16S rRNA gene sequences of *Desulfovibrio africanus subsp. africanus* DSM 2603 and microbial strains belonging to the training set of PhiSpy.

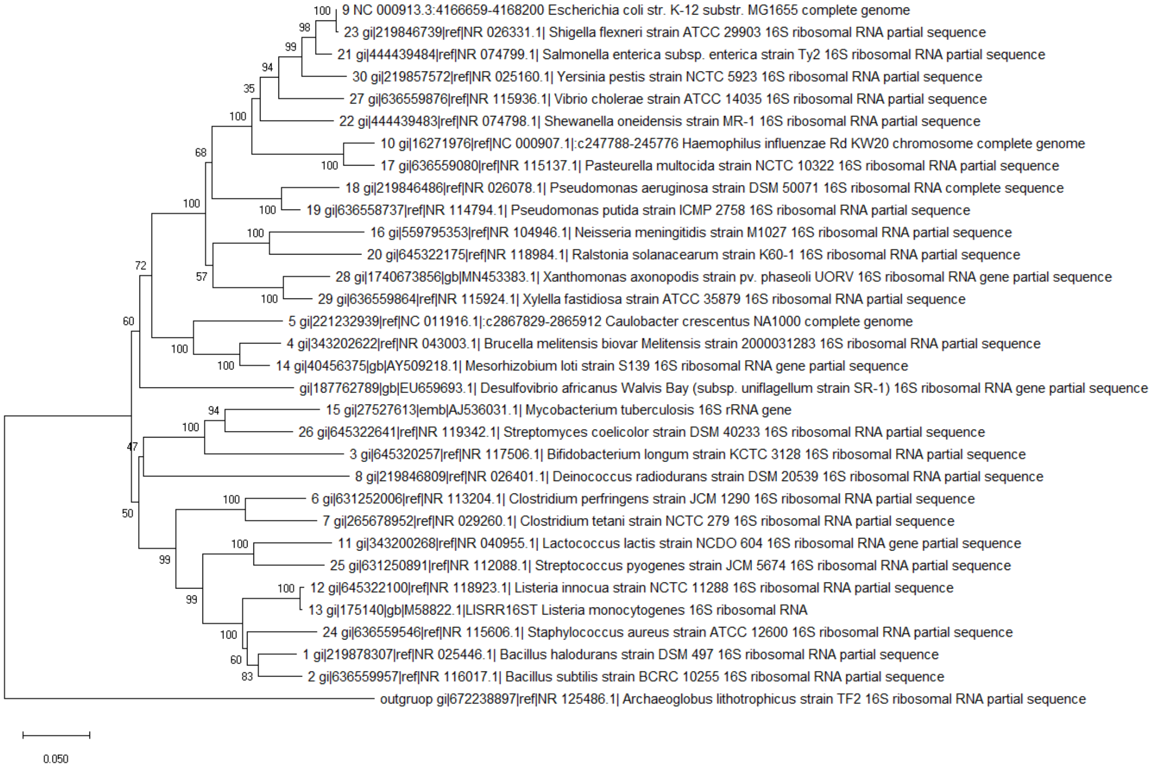

Figure S1.48: Phylogenetic tree of 16S rRNA gene sequences of *Desulfovibrio africanus Walvis Bay* and microbial strains belonging to the training set of PhiSpy.

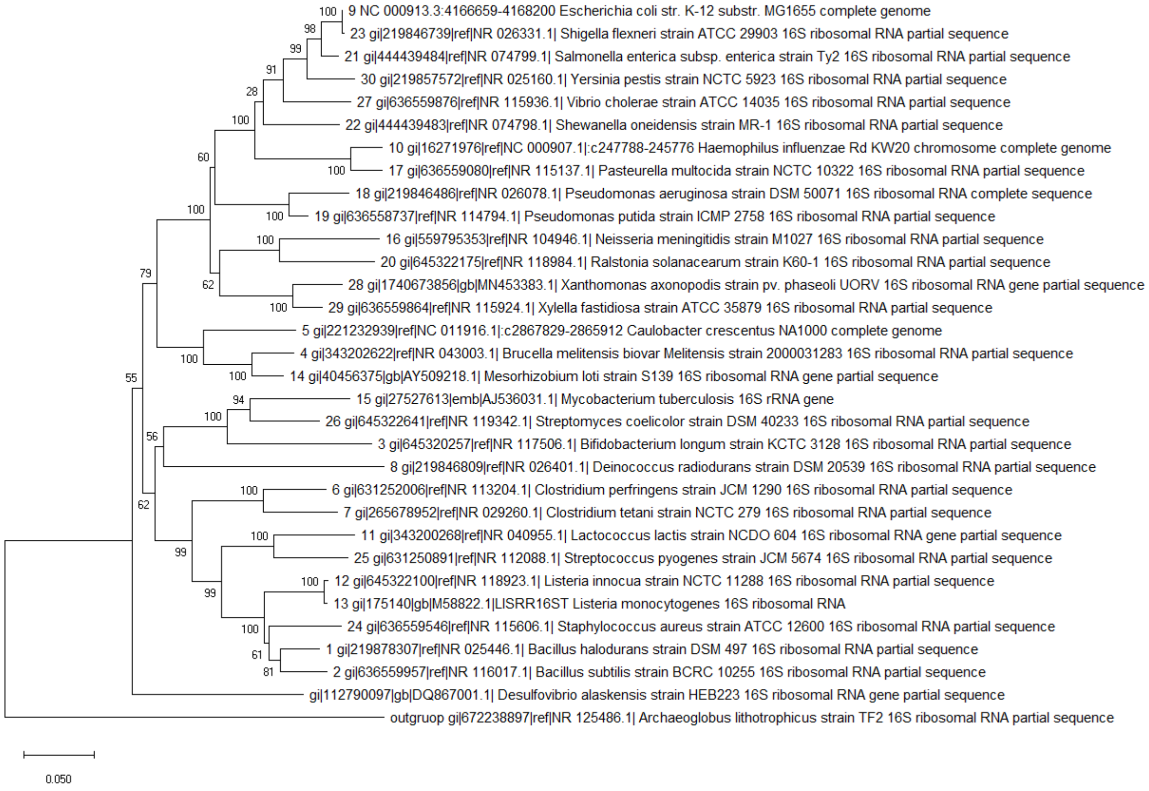

Figure S1.49: Phylogenetic tree of 16S rRNA gene sequences of *Desulfovibrio alaskensis* and microbial strains belonging to the training set of PhiSpy.

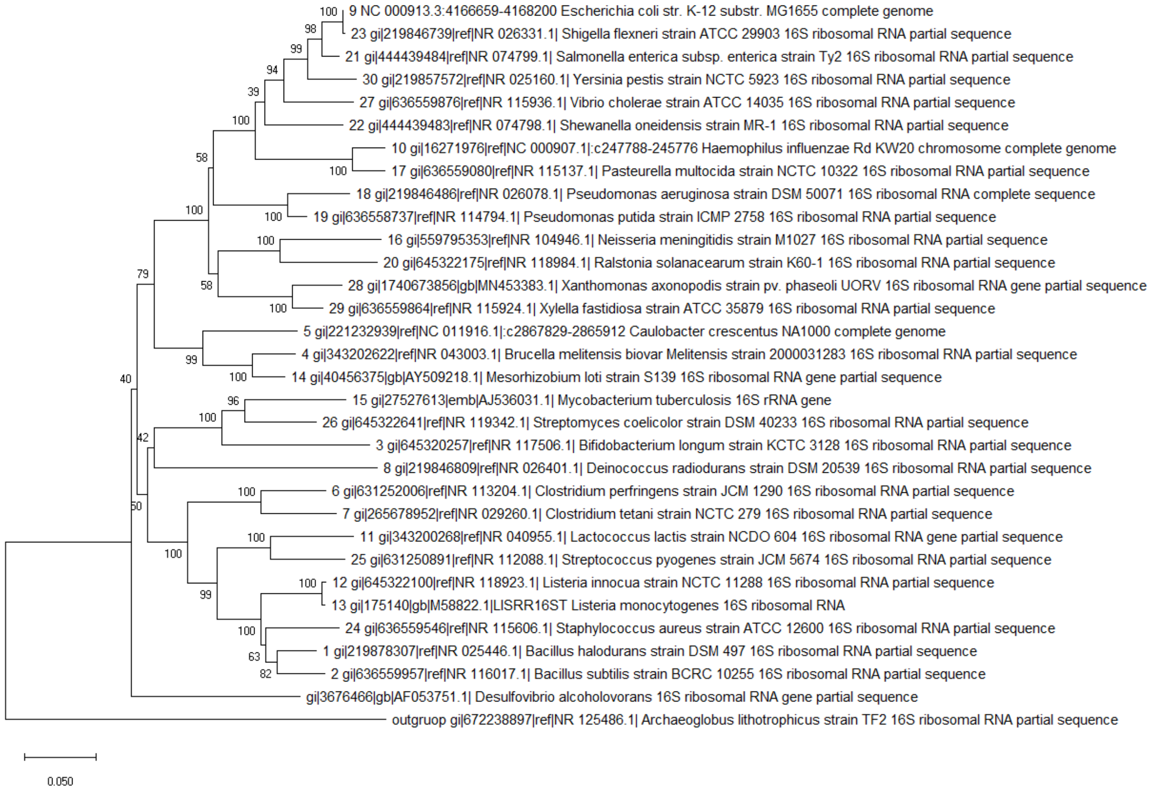

Figure S1.50: Phylogenetic tree of 16S rRNA gene sequences of *Desulfovibrio alcoholivorans* and microbial strains belonging to the training set of PhiSpy.

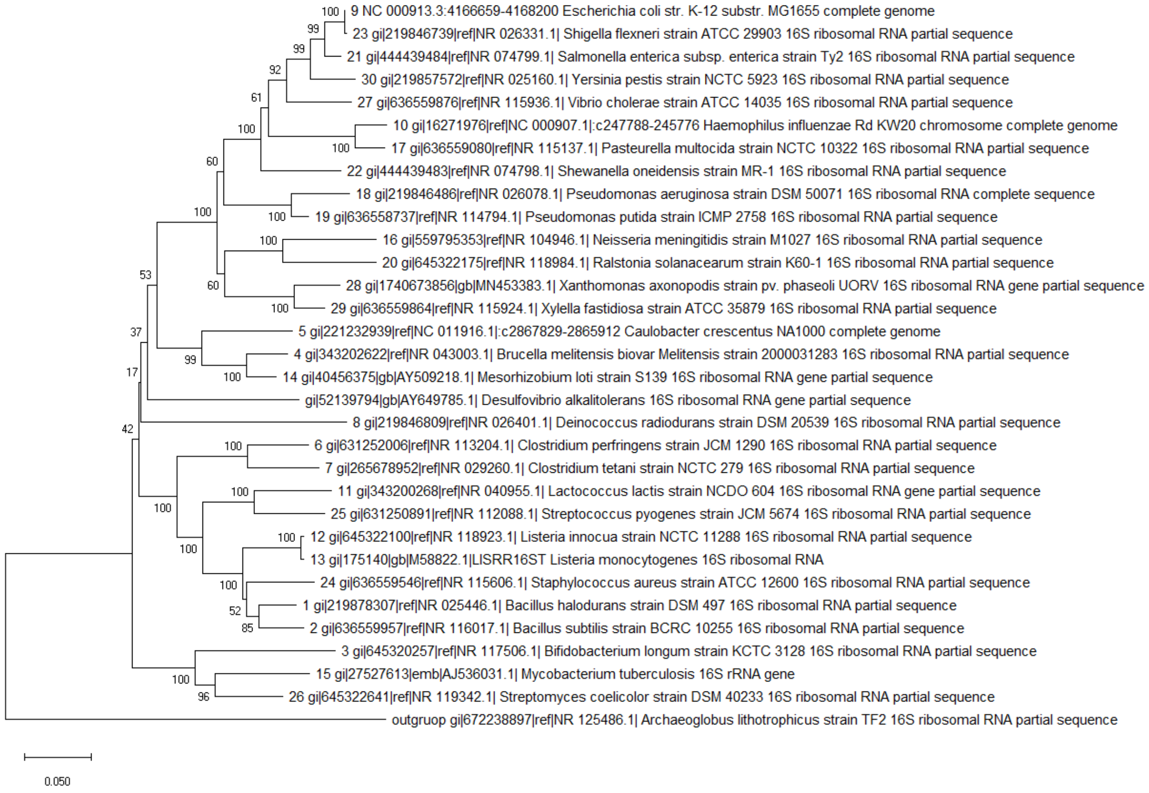

Figure S1.51: Phylogenetic tree of 16S rRNA gene sequences of *Desulfovibrio alkalitolerans* and microbial strains belonging to the training set of PhiSpy.

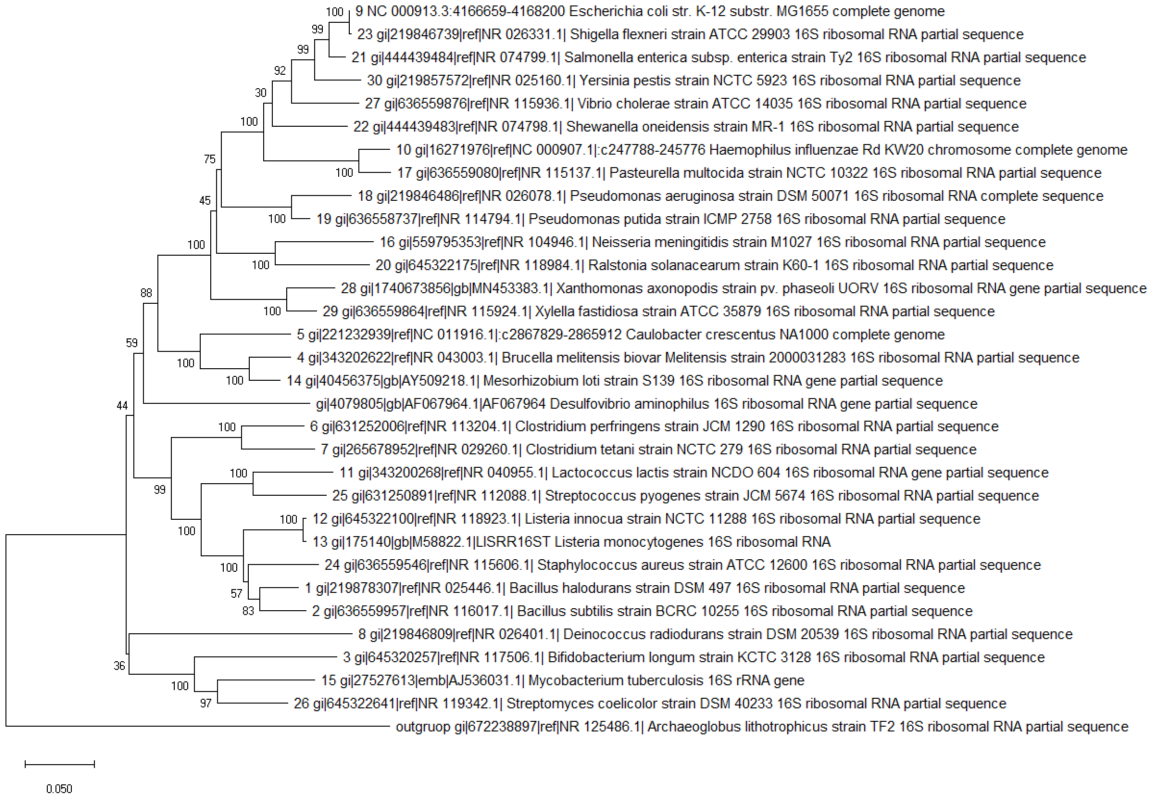

Figure S1.52: Phylogenetic tree of 16S rRNA gene sequences of *Desulfovibrio aminophilus* DSM 12254 and microbial strains belonging to the training set of PhiSpy.

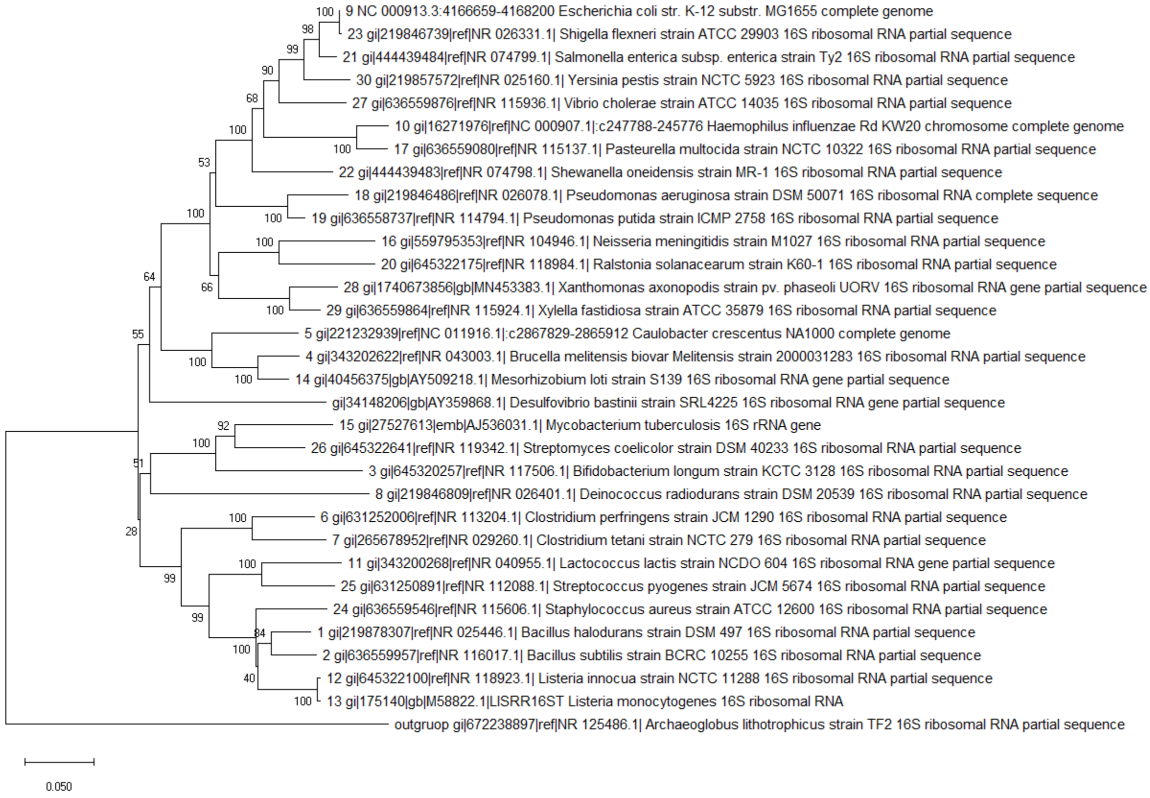

Figure S1.53: Phylogenetic tree of 16S rRNA gene sequences of *Desulfovibrio bastinii strain* SRL4225 and microbial strains belonging to the training set of PhiSpy.

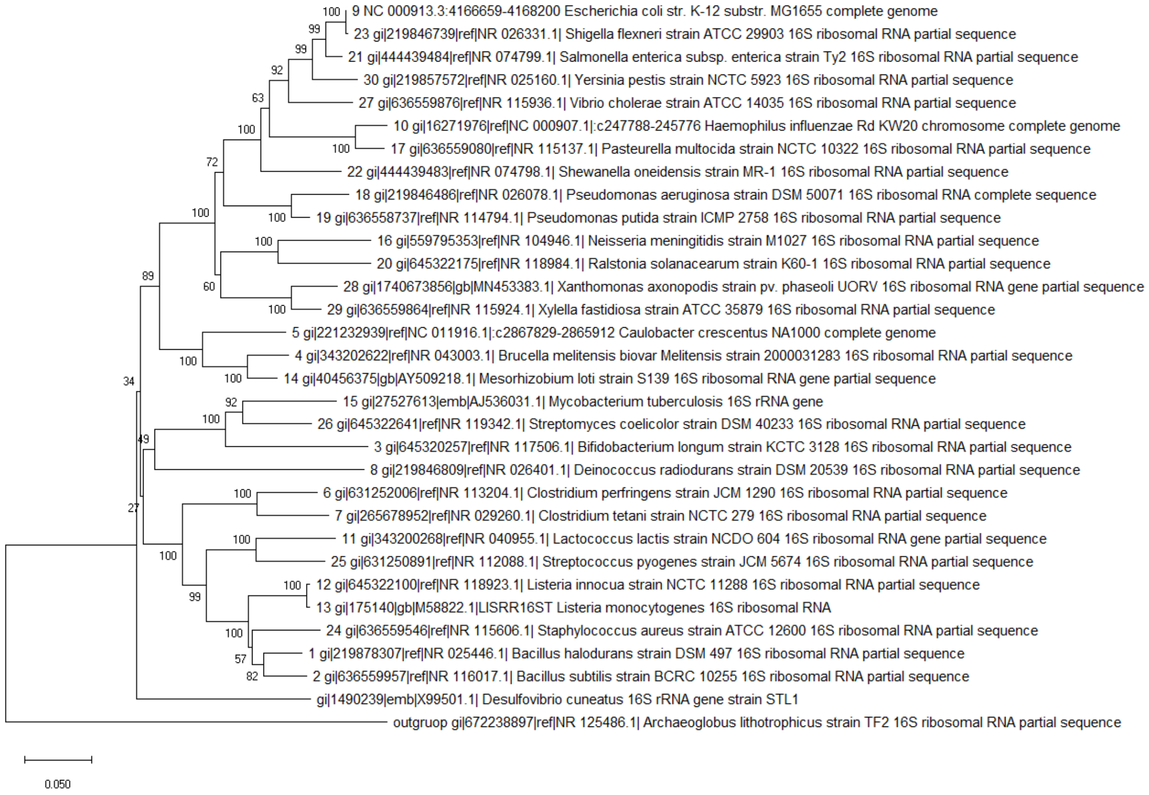

Figure S1.54: Phylogenetic tree of 16S rRNA gene sequences of *Desulfovibrio cuneatus* DSM 11391 and microbial strains belonging to the training set of PhiSpy.

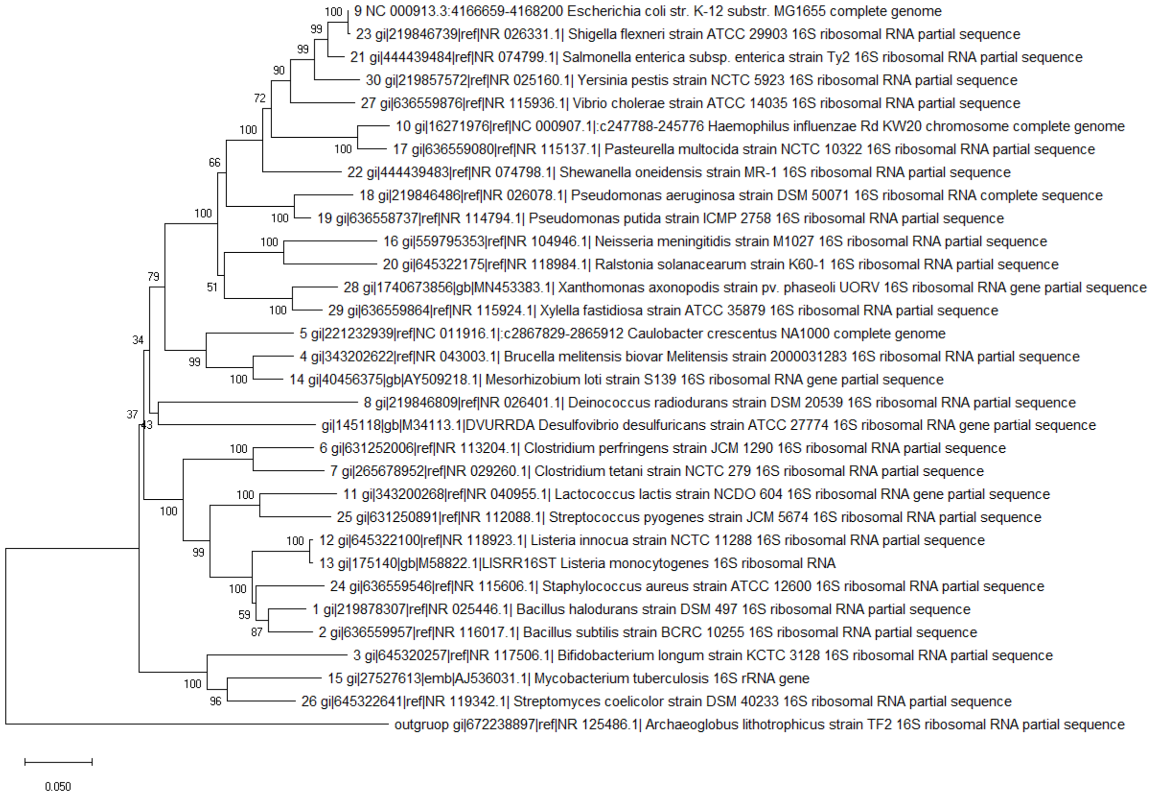

Figure S1.55: Phylogenetic tree of 16S rRNA gene sequences of *Desulfovibrio desulfuricans* 642 and microbial strains belonging to the training set of PhiSpy.

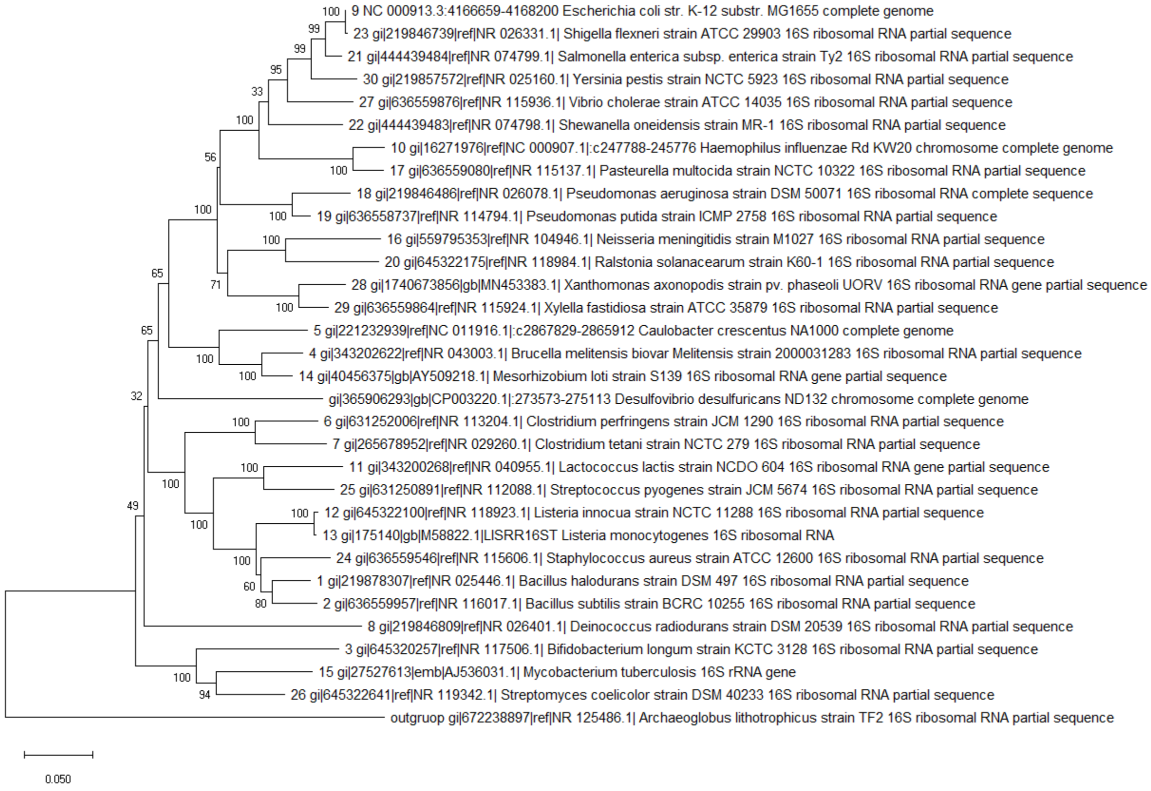

Figure S1.56: Phylogenetic tree of 16S rRNA gene sequences of *Desulfovibrio desulfuricans* ND132 and microbial strains belonging to the training set of PhiSpy.

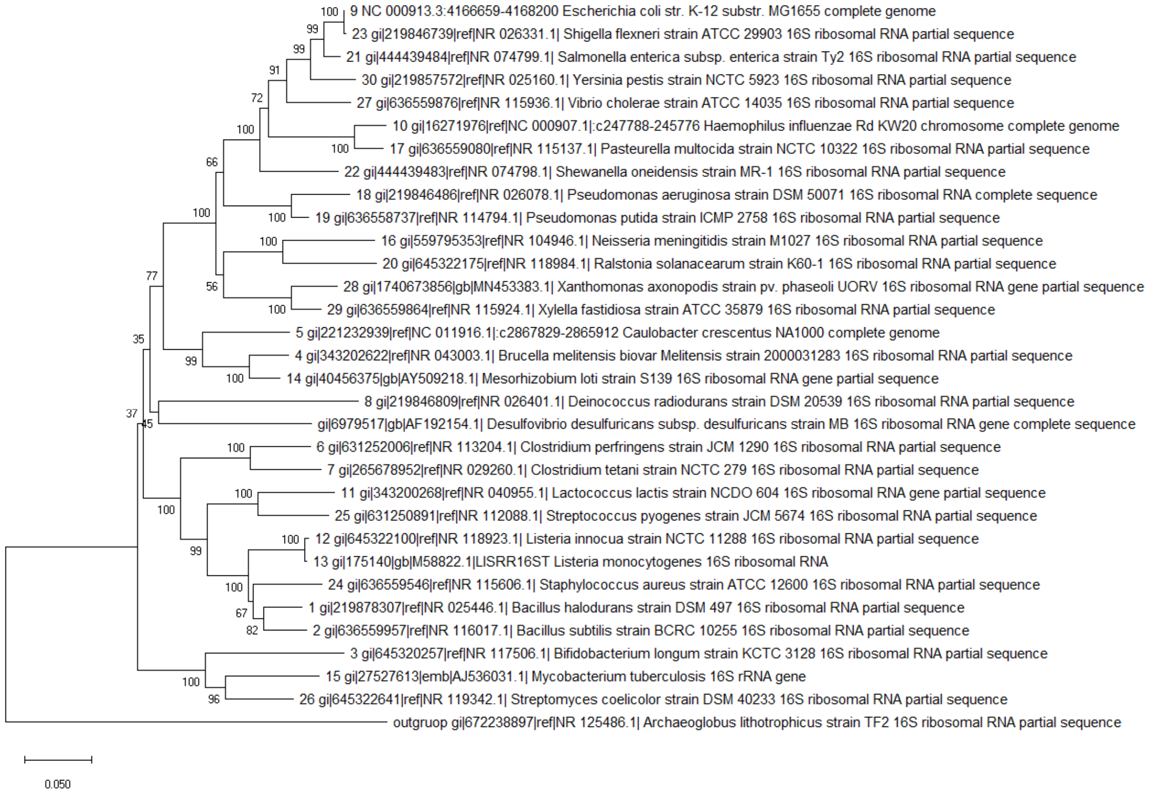

Figure S1.57: Phylogenetic tree of 16S rRNA gene sequences of *Desulfovibrio desulfuricans subsp. desulfuricans* strain MB and microbial strains belonging to the training set of PhiSpy.

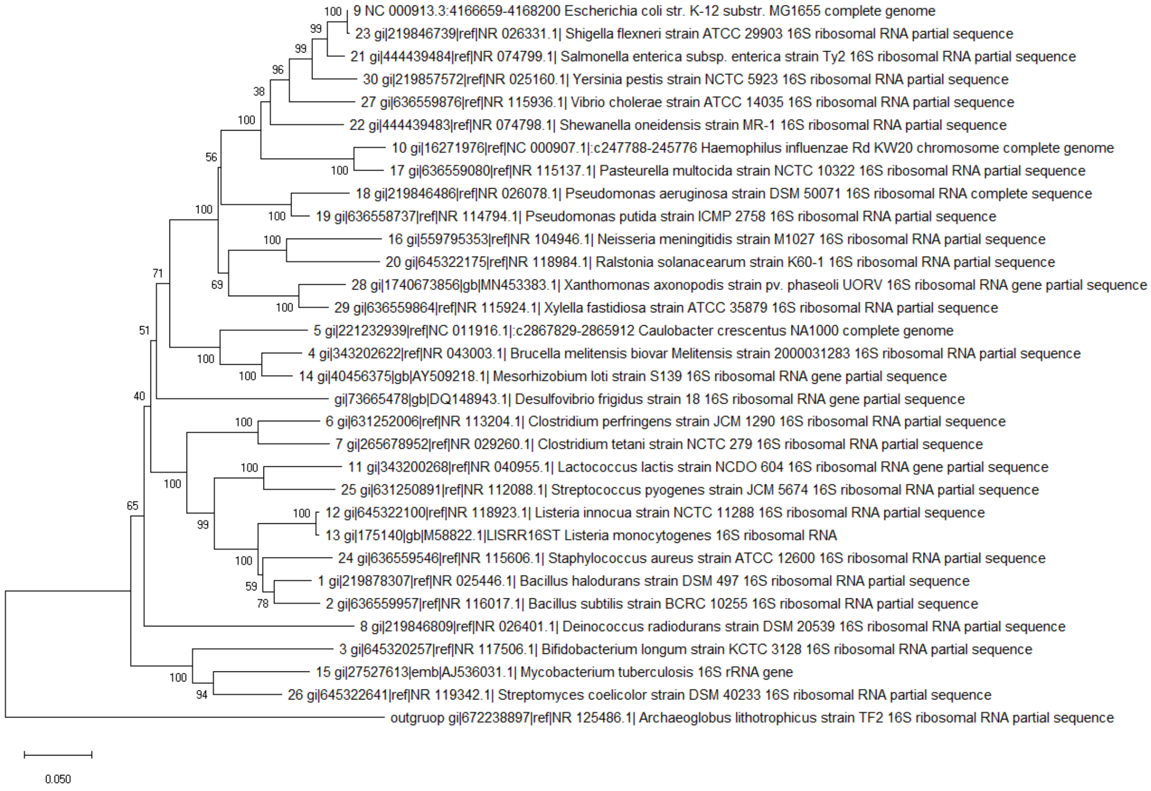

Figure S1.58: Phylogenetic tree of 16S rRNA gene sequences of *Desulfovibrio frigidus* DSM 17176 and microbial strains belonging to the training set of PhiSpy.

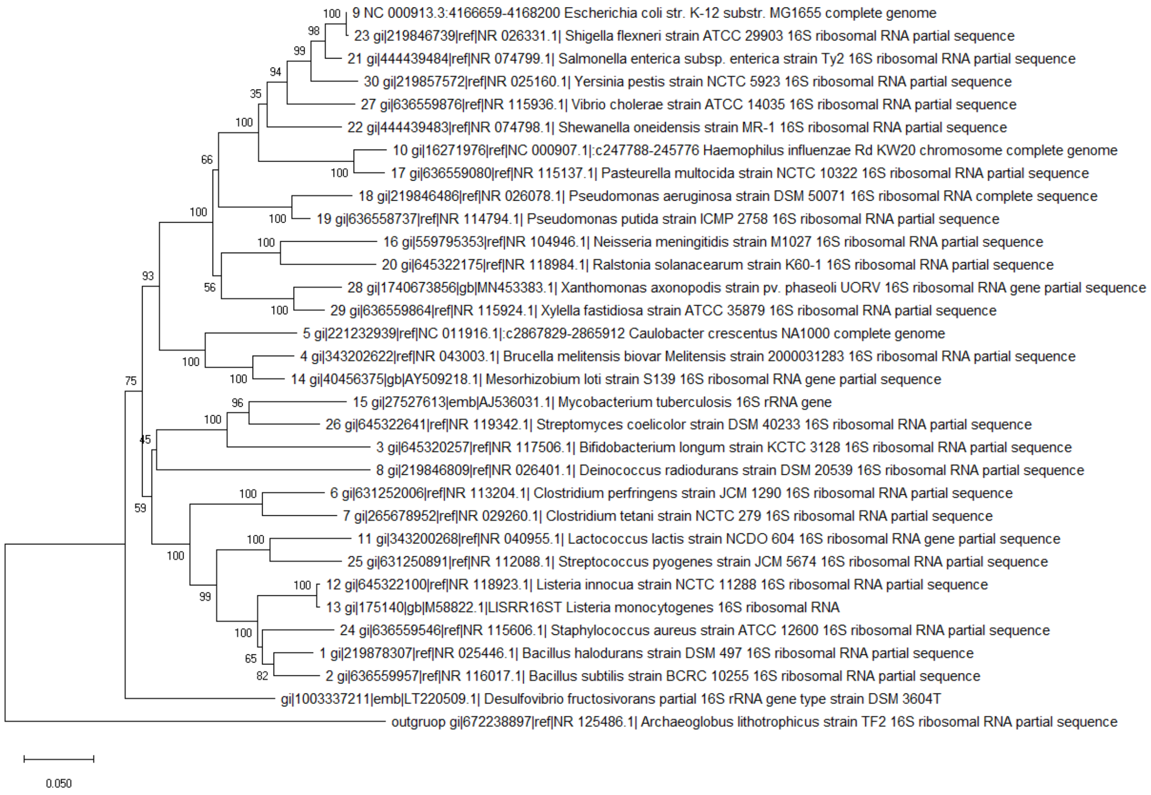

Figure S1.59: Phylogenetic tree of 16S rRNA gene sequences of *Desulfovibrio fructosivorans* JJ and microbial strains belonging to the training set of PhiSpy.

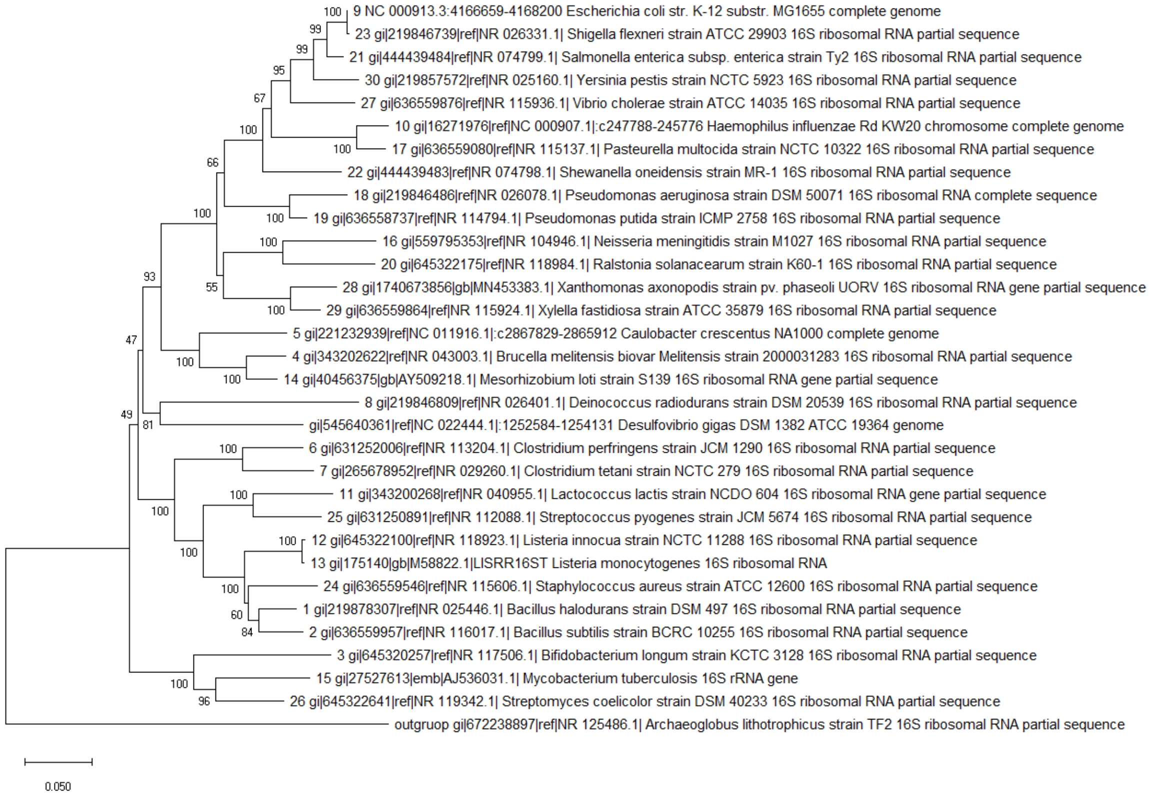

Figure S1.60: Phylogenetic tree of 16S rRNA gene sequences of *Desulfovibrio gigas* DSM 1382 and microbial strains belonging to the training set of PhiSpy.

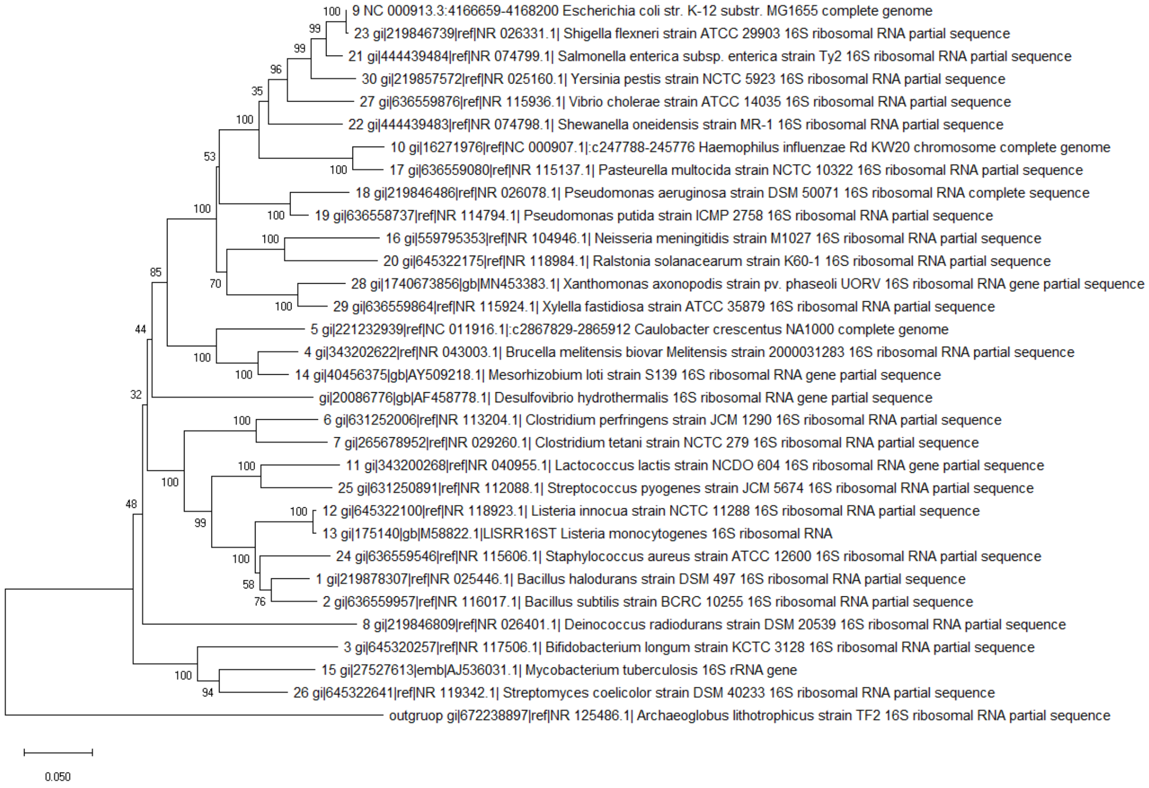

Figure S1.61: Phylogenetic tree of 16S rRNA gene sequences of *Desulfovibrio hydrothermalis* DSM 14728 and microbial strains belonging to the training set of PhiSpy.

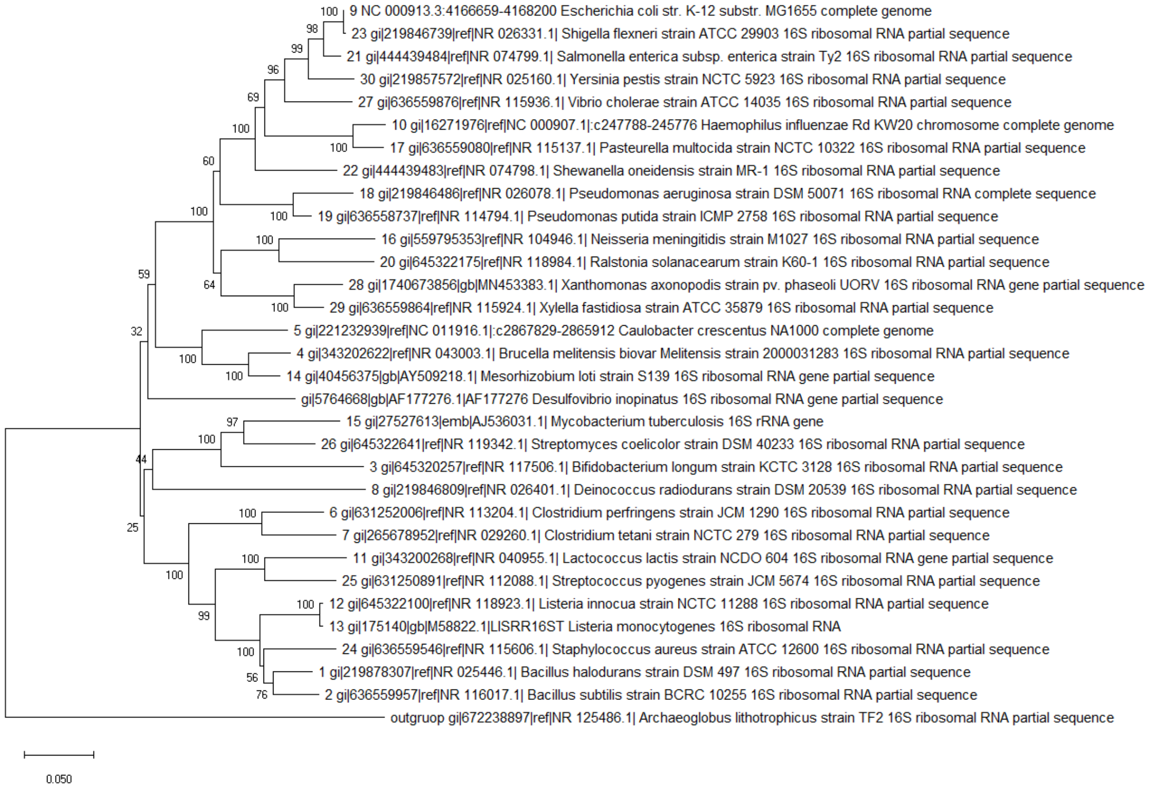

Figure S1.62: Phylogenetic tree of 16S rRNA gene sequences of *Desulfovibrio inopinatus* DSM 10711 and microbial strains belonging to the training set of PhiSpy.

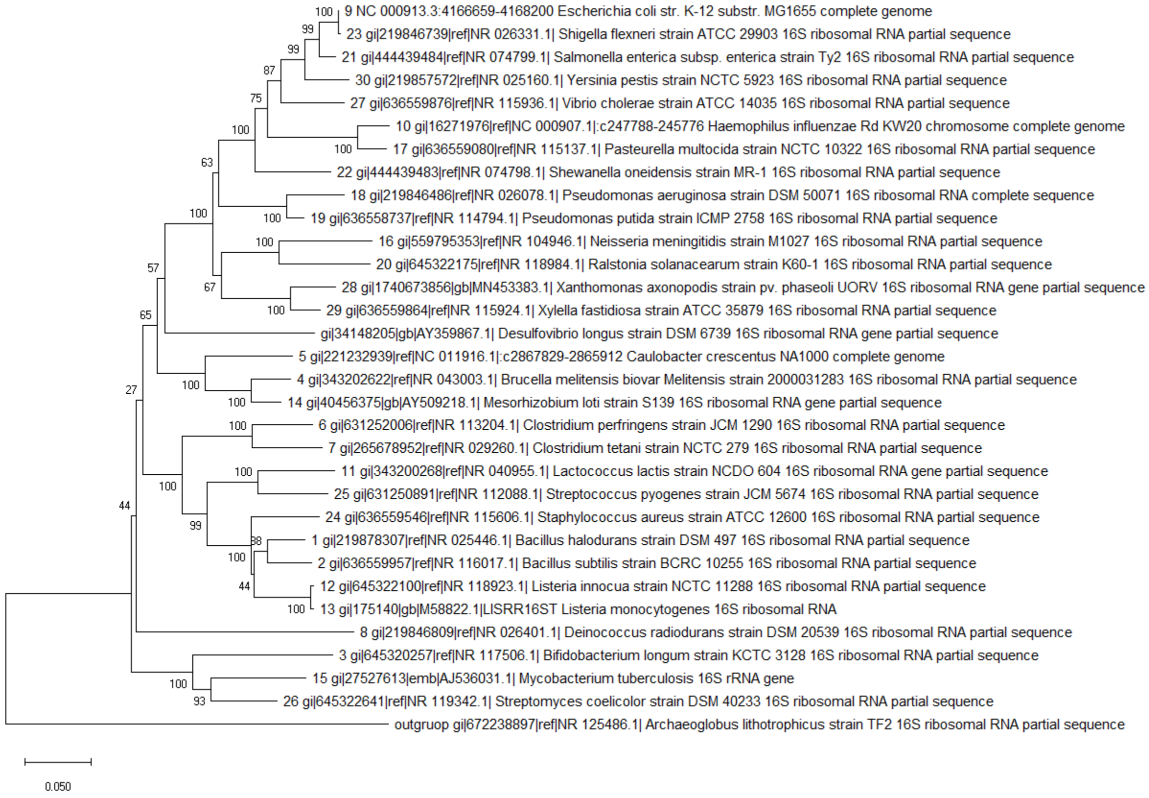

Figure S1.63: Phylogenetic tree of 16S rRNA gene sequences of *Desulfovibrio longus strain* DSM 6739 and microbial strains belonging to the training set of PhiSpy.

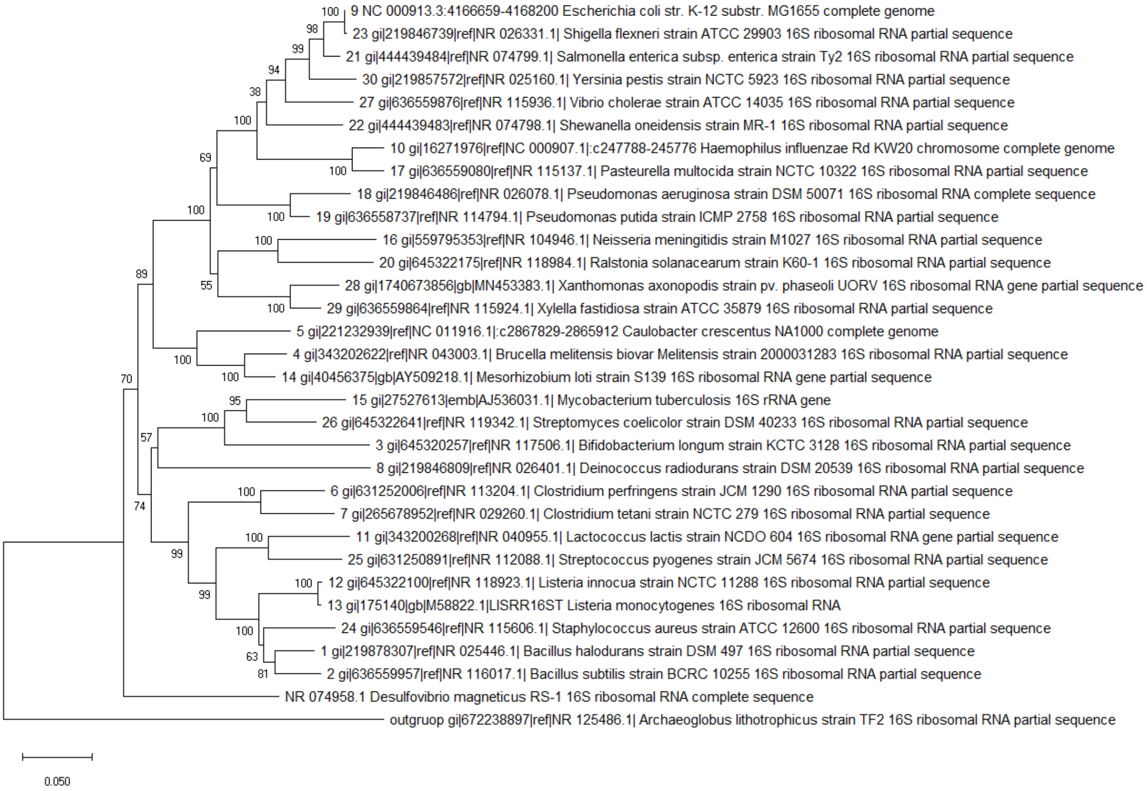

Figure S1.64: Phylogenetic tree of 16S rRNA gene sequences of *Desulfovibrio magneticus* RS-1 and microbial strains belonging to the training set of PhiSpy.

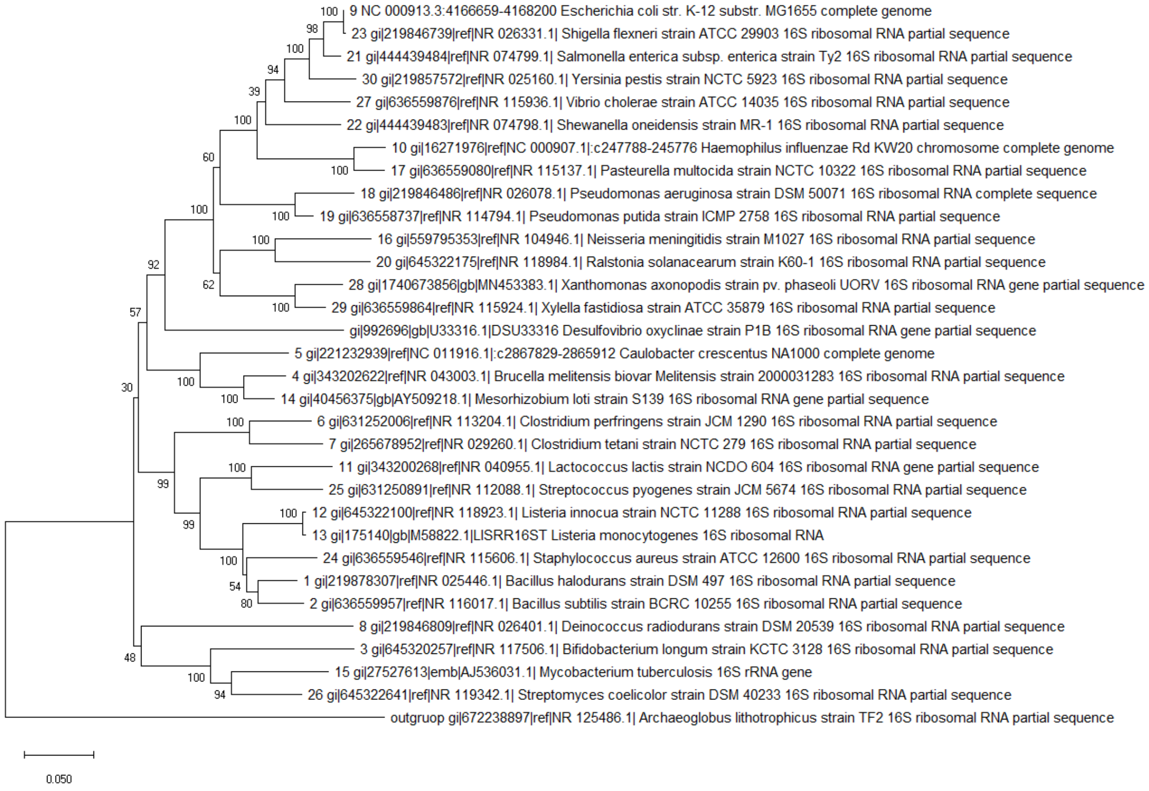

Figure S1.65: Phylogenetic tree of 16S rRNA gene sequences of *Desulfovibrio oxyclinae* strain P1B and microbial strains belonging to the training set of PhiSpy.

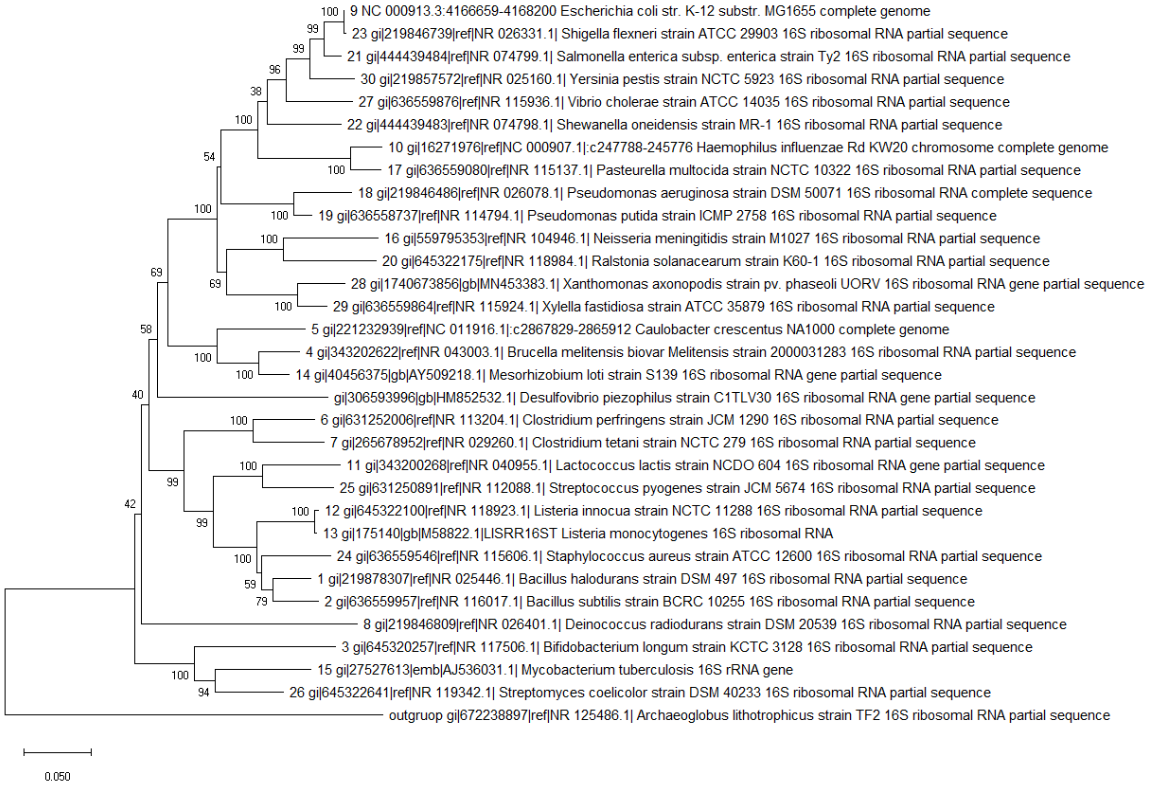

Figure S1.66: Phylogenetic tree of 16S rRNA gene sequences of *Desulfovibrio piezophilus* strain C1TLV30 and microbial strains belonging to the training set of PhiSpy.

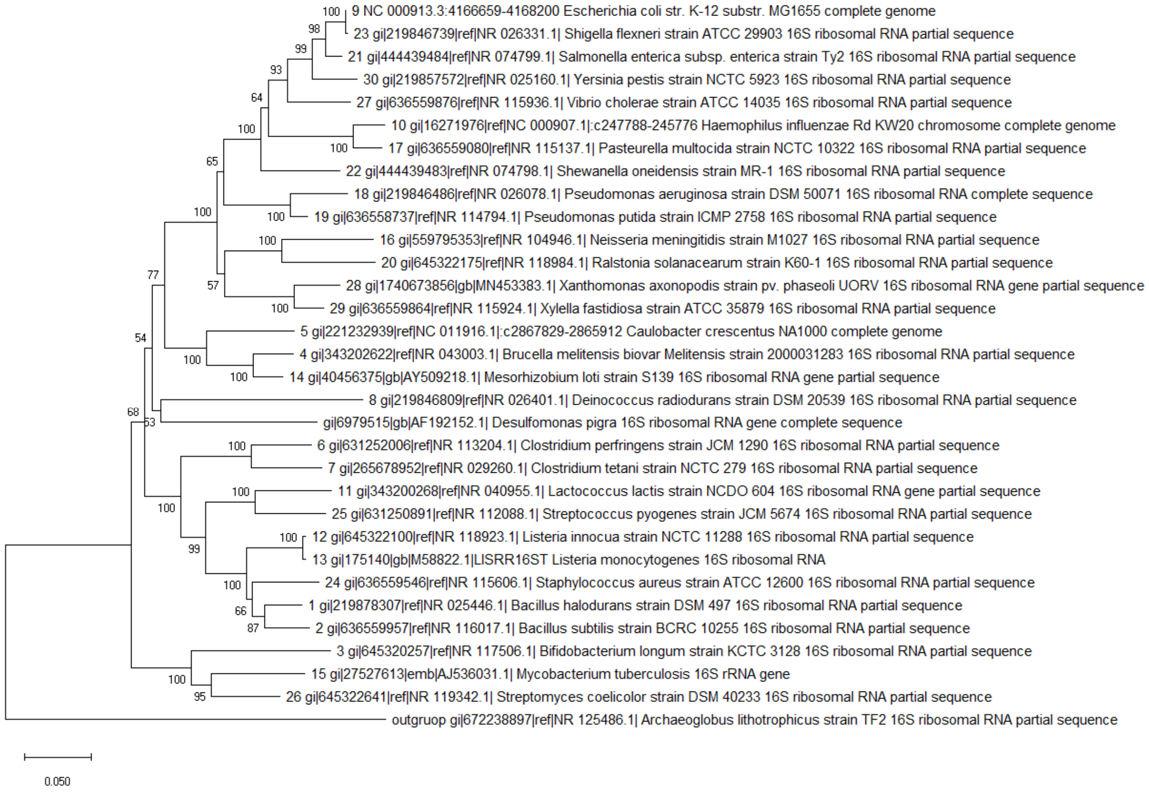

Figure S1.67: Phylogenetic tree of 16S rRNA gene sequences of *Desulfovibrio piger* ATCC 29098 and microbial strains belonging to the training set of PhiSpy.

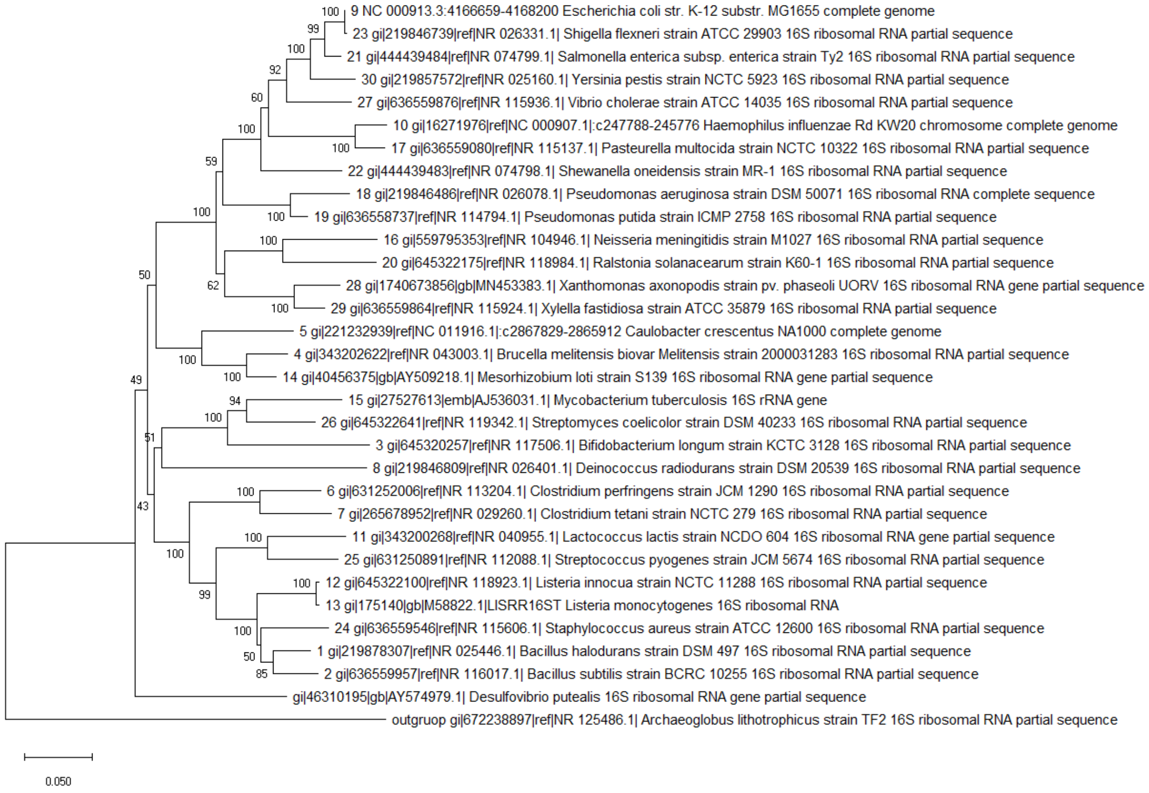

Figure S1.68: Phylogenetic tree of 16S rRNA gene sequences of *Desulfovibrio putealis* DSM 16056 and microbial strains belonging to the training set of PhiSpy.

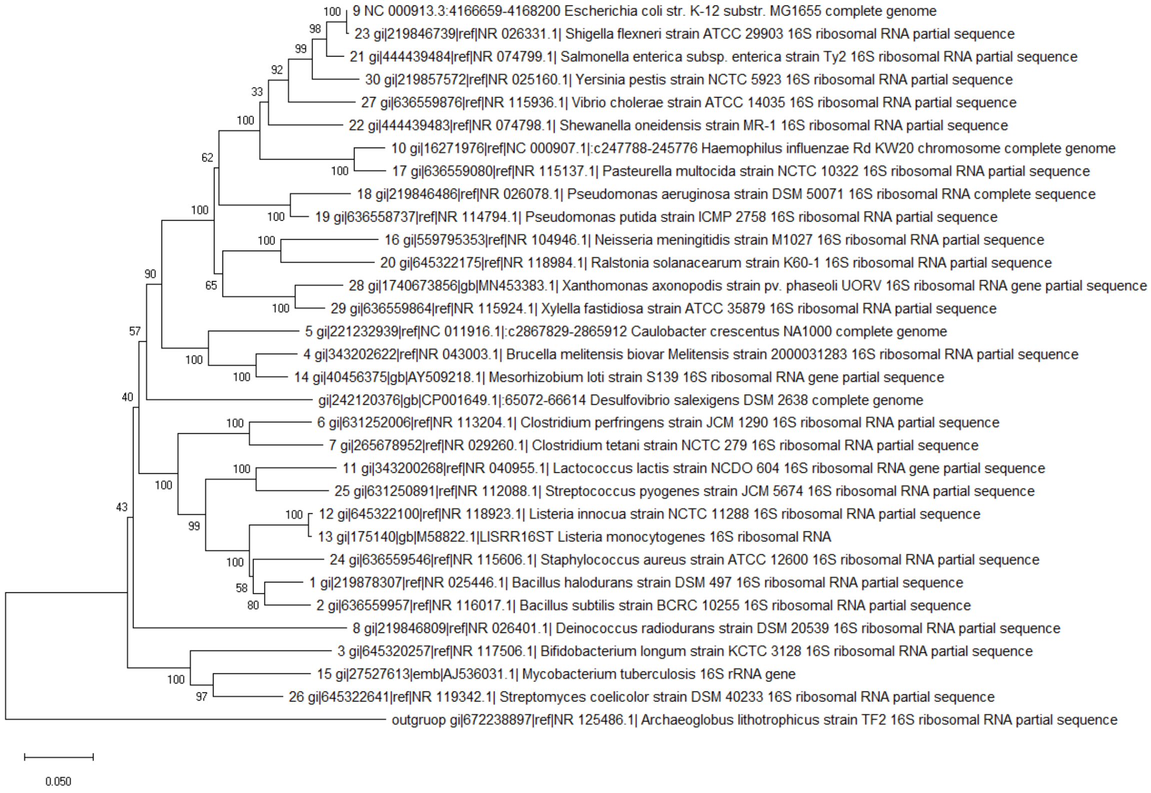

Figure S1.69: Phylogenetic tree of 16S rRNA gene sequences of *Desulfovibrio salexigens* and microbial strains belonging to the training set of PhiSpy.

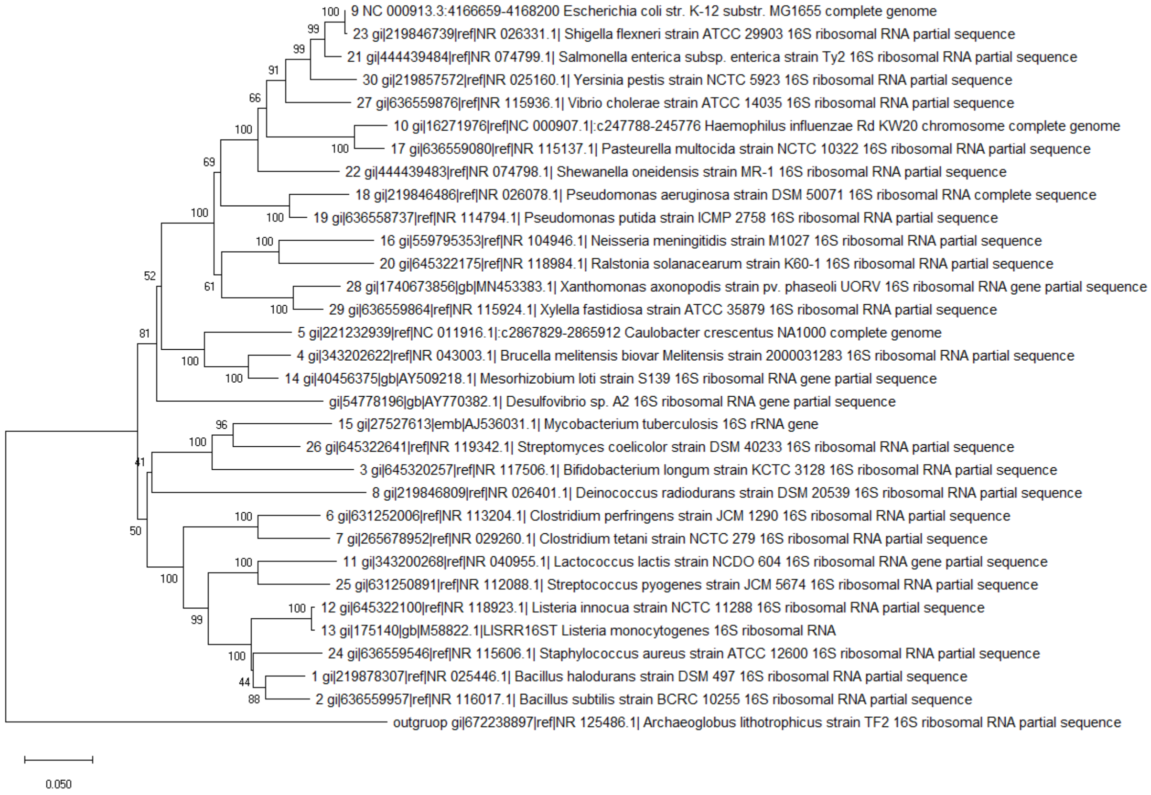

Figure S1.70: Phylogenetic tree of 16S rRNA gene sequences of *Desulfovibrio sp.* A2 and microbial strains belonging to the training set of PhiSpy.

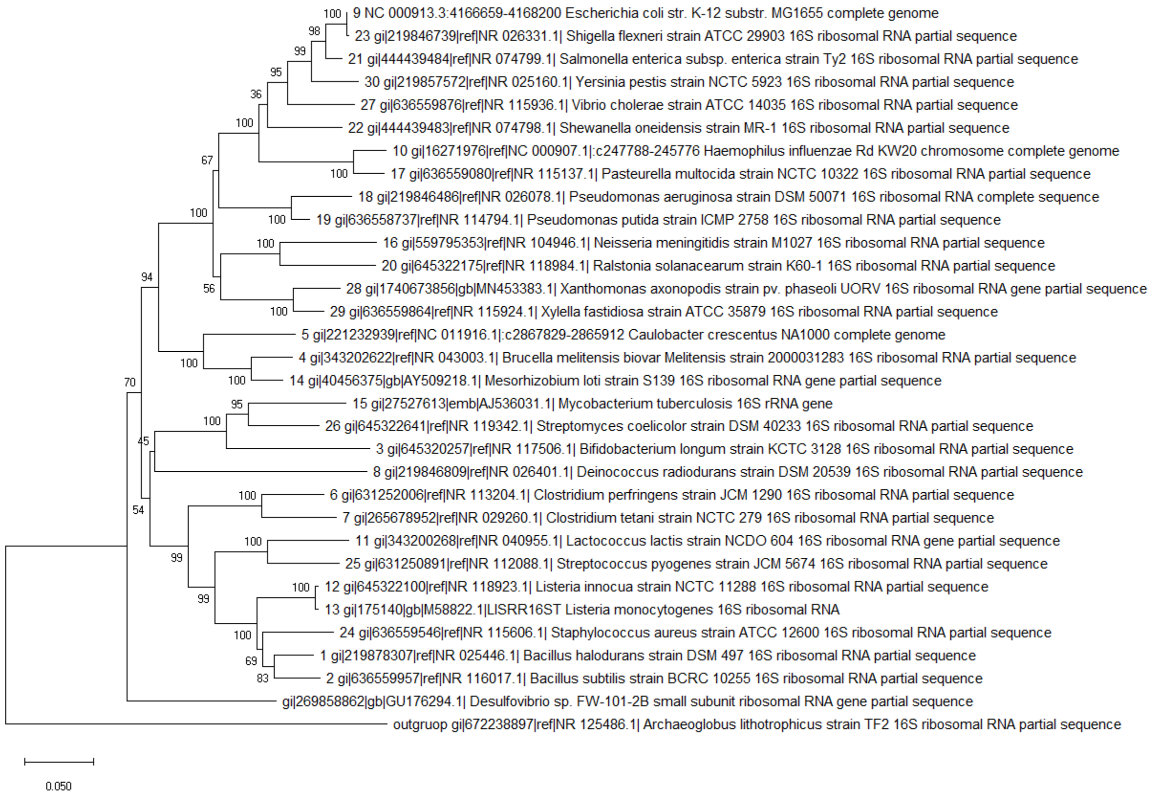

Figure S1.71: Phylogenetic tree of 16S rRNA gene sequences of *Desulfovibrio sp.* FW1012B and microbial strains belonging to the training set of PhiSpy.

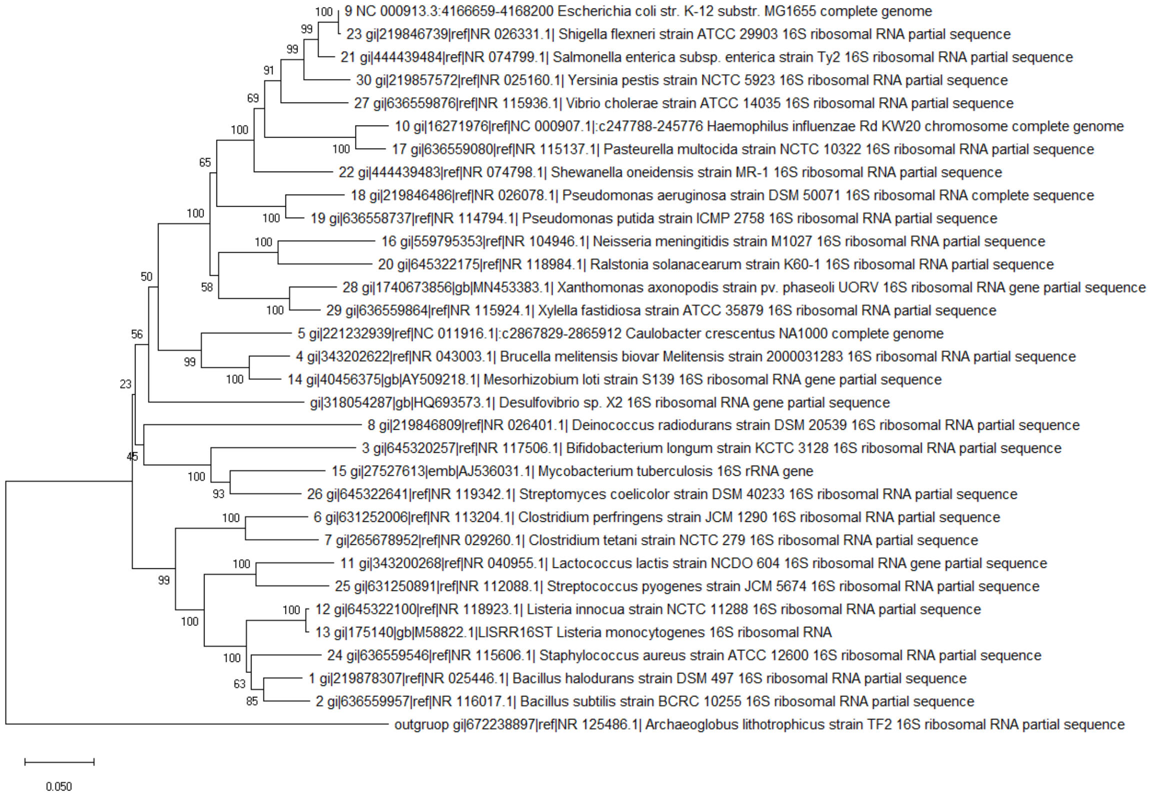

Figure S1.72: Phylogenetic tree of 16S rRNA gene sequences of *Desulfovibrio sp.* X2 and microbial strains belonging to the training set of PhiSpy.

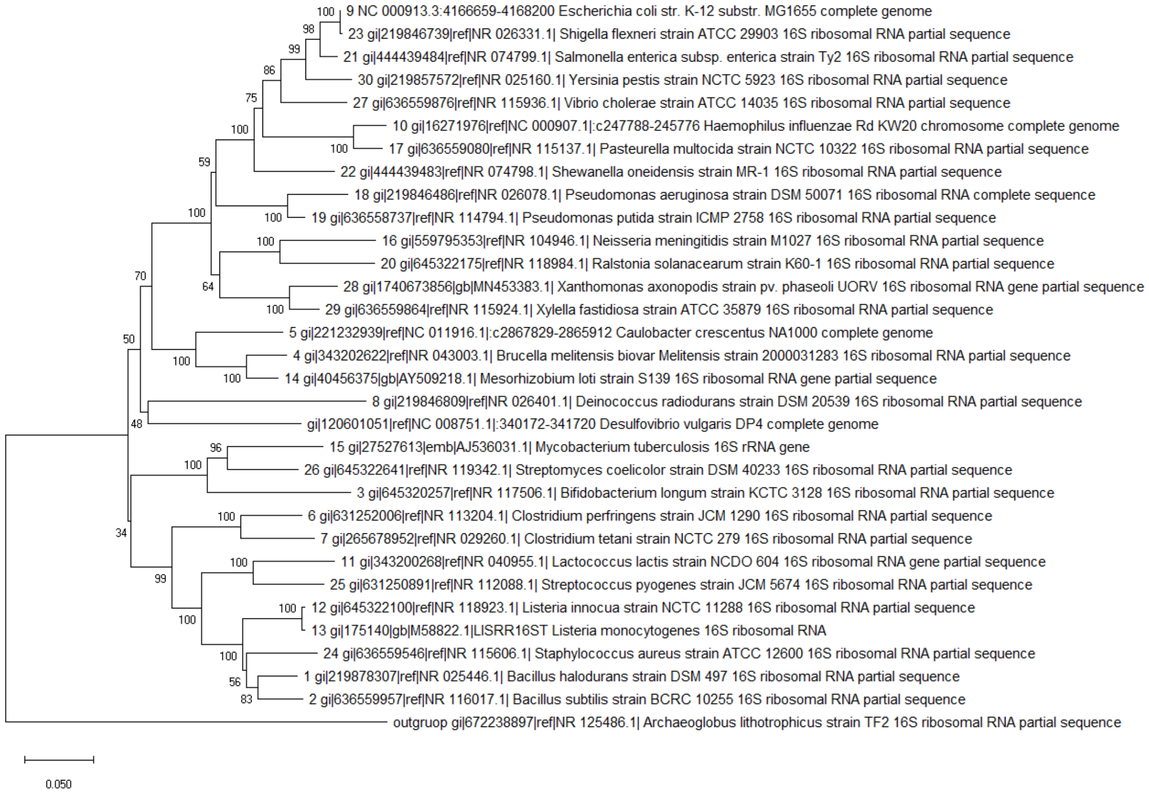

Figure S1.73: Phylogenetic tree of 16S rRNA gene sequences of *Desulfovibrio vulgaris* DP4 and microbial strains belonging to the training set of PhiSpy.

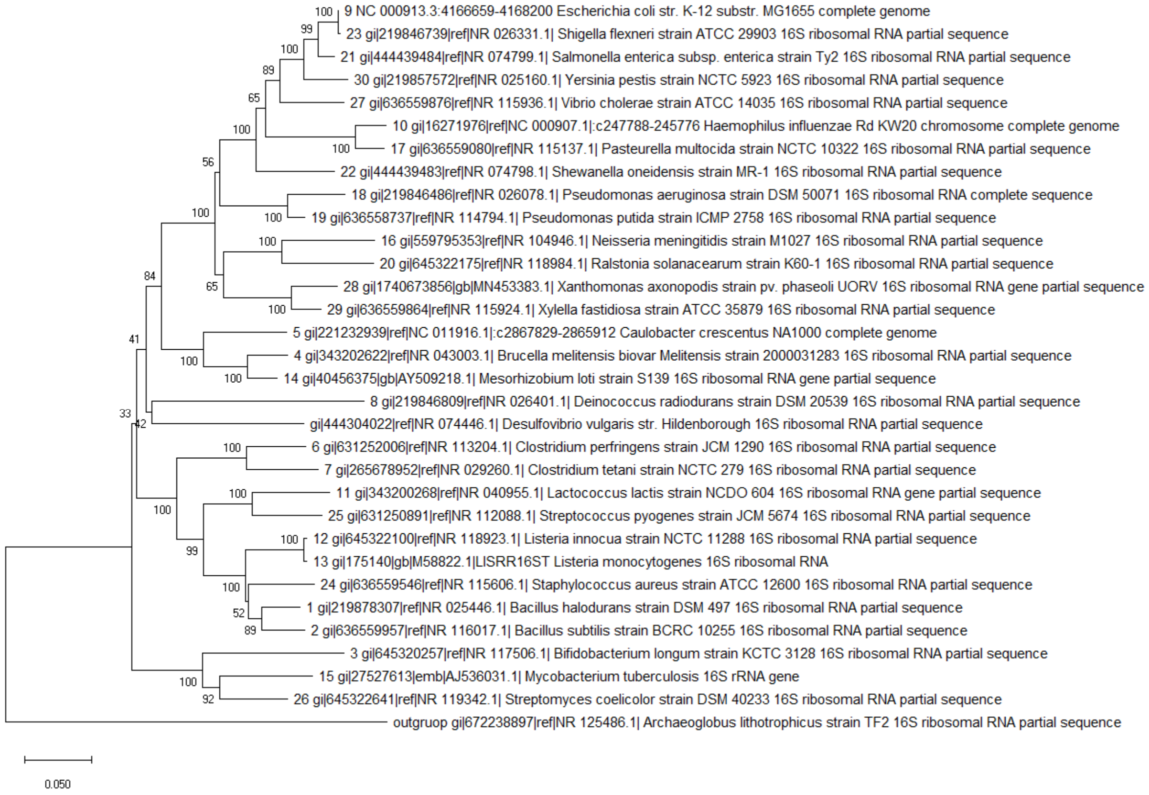

Figure S1.74: Phylogenetic tree of 16S rRNA gene sequences of *Desulfovibrio vulgaris* str. *Hildenborough* and microbial strains belonging to the training set of PhiSpy.

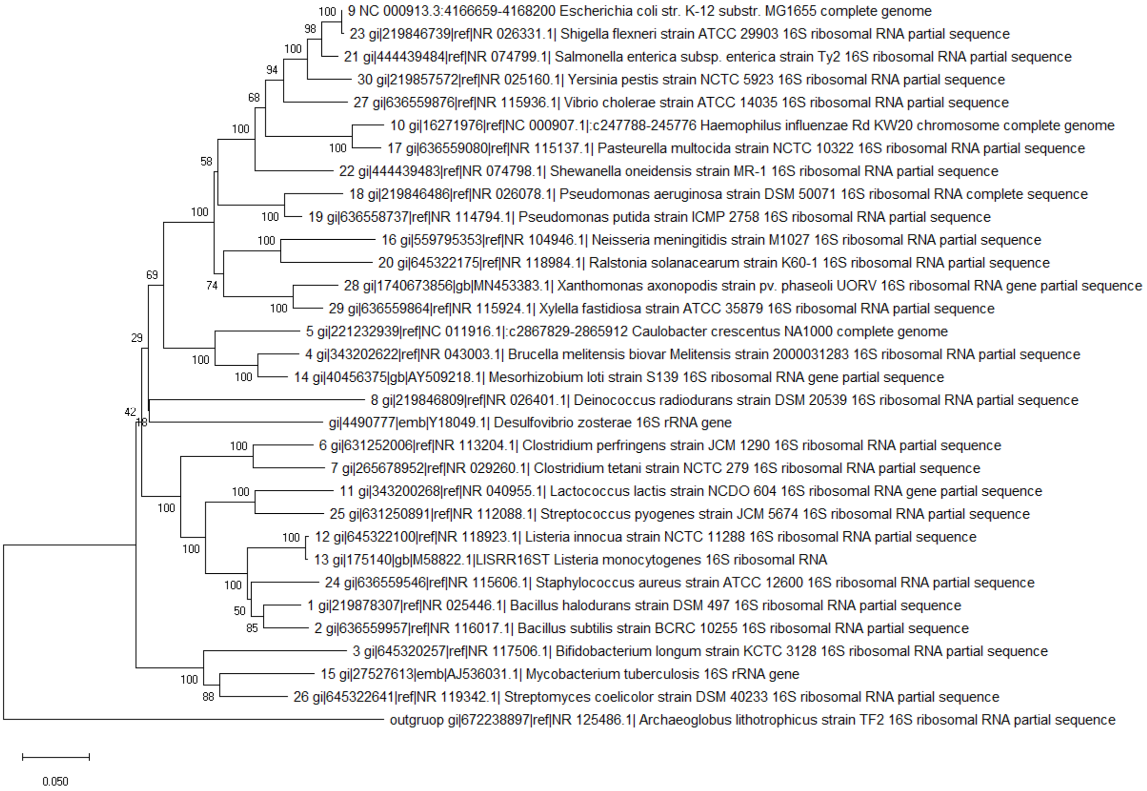

Figure S1.75: Phylogenetic tree of 16S rRNA gene sequences of *Desulfovibrio zosterae* DSM 11974 and microbial strains belonging to the training set of PhiSpy.

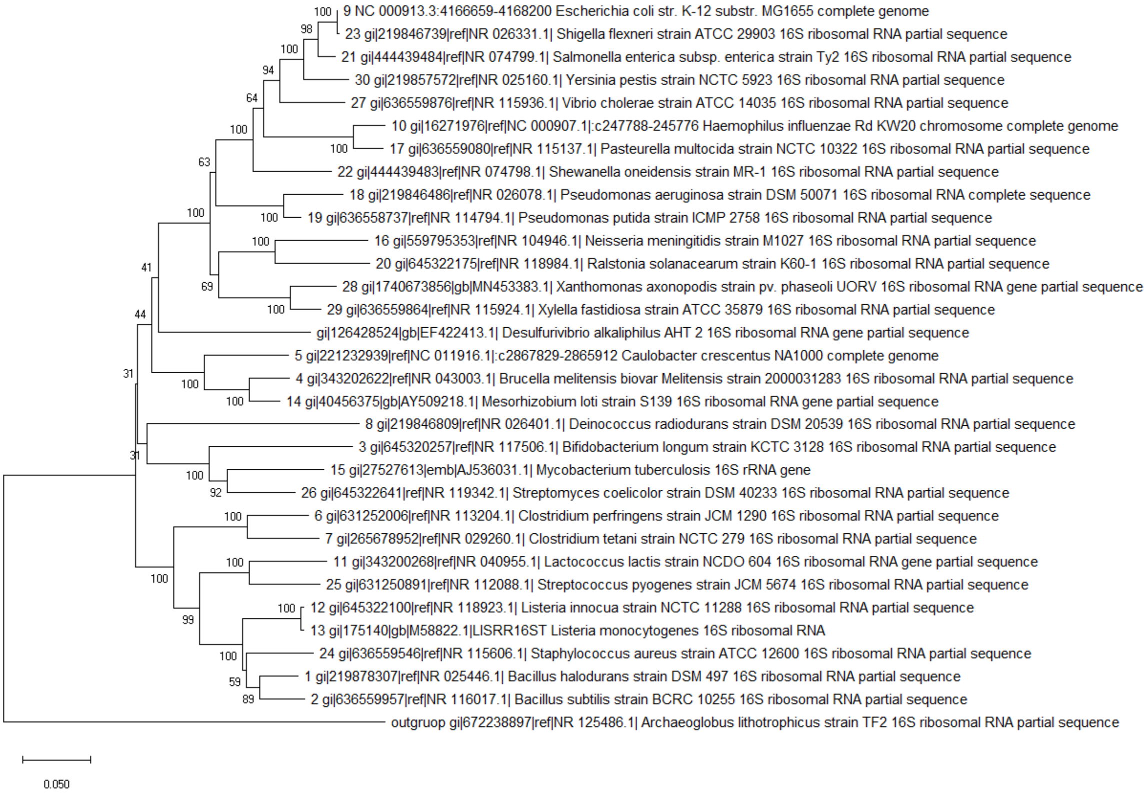

Figure S1.76: Phylogenetic tree of 16S rRNA gene sequences of *Desulfurivibrio alkaliphilus* AHT2 and microbial strains belonging to the training set of PhiSpy.

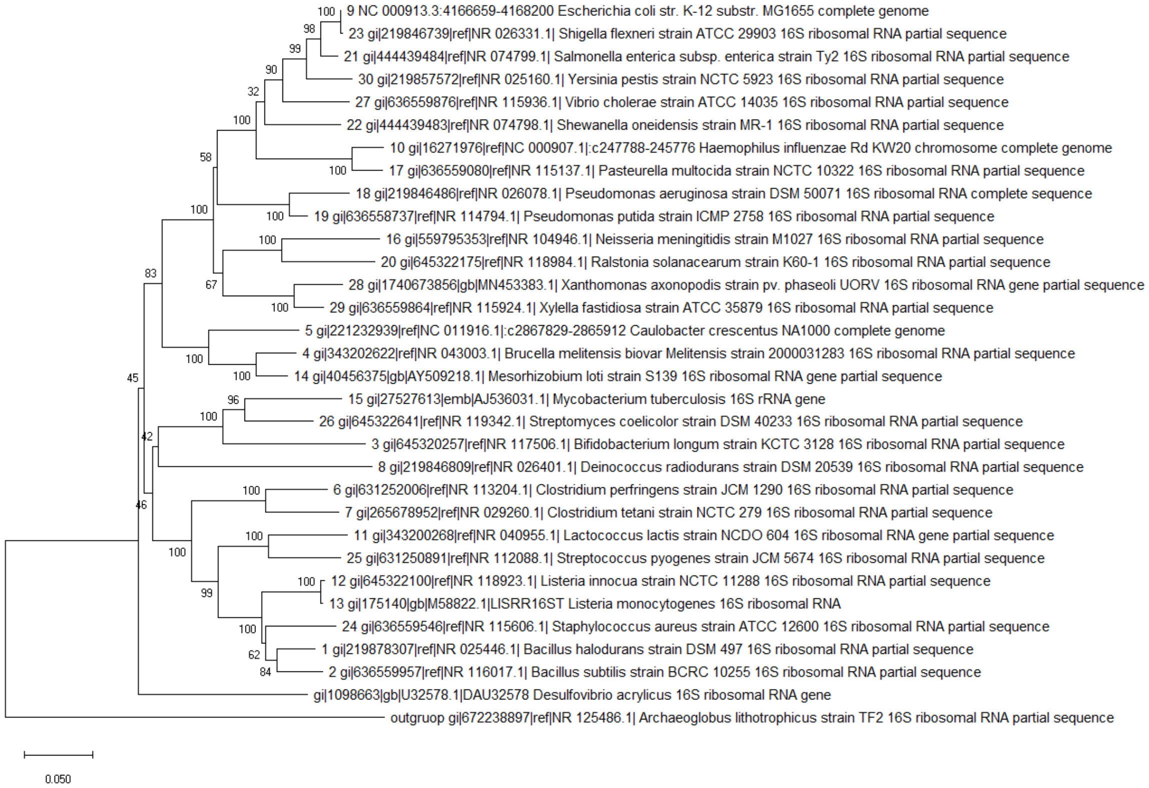

Figure S1.77: Phylogenetic tree of 16S rRNA gene sequences of *Halodesulfovibrio aestuarii* DSM 10141 and microbial strains belonging to the training set of PhiSpy.

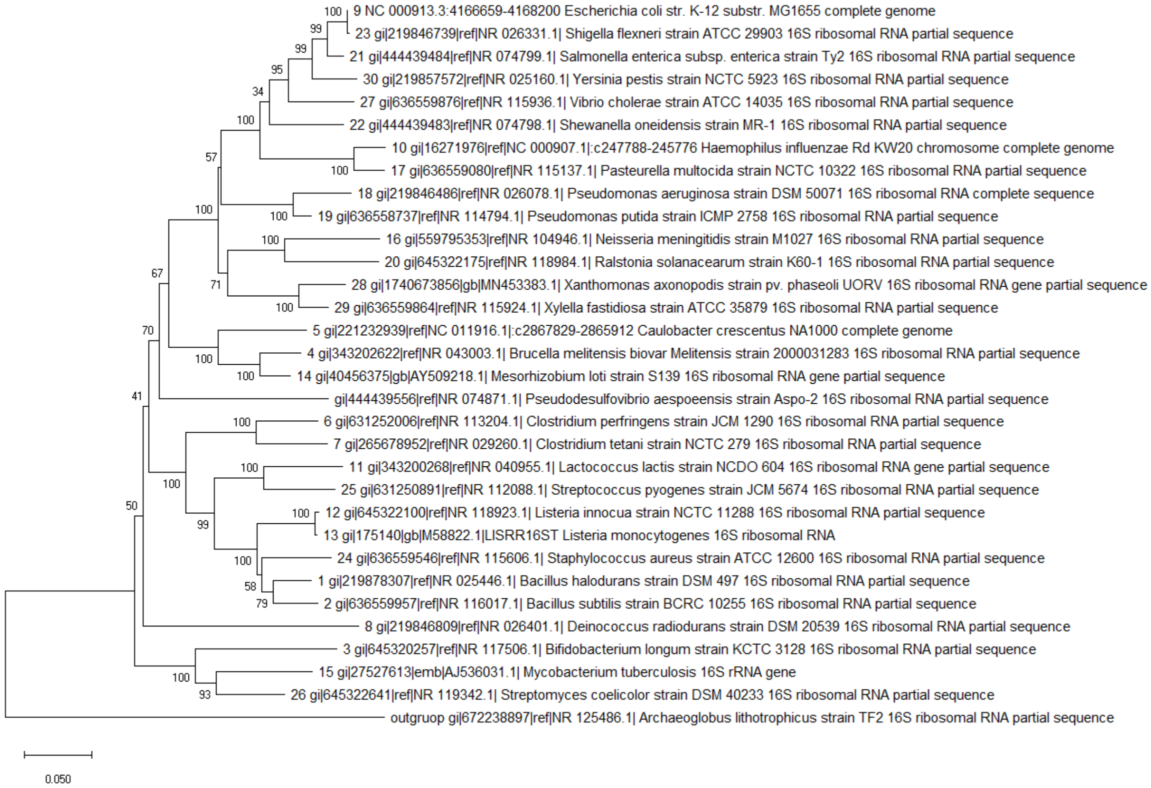

Figure S1.78: Phylogenetic tree of 16S rRNA gene sequences of *Pseudodesulfovibrio aespoeensis* strain Aspo-2 and microbial strains belonging to the training set of PhiSpy.

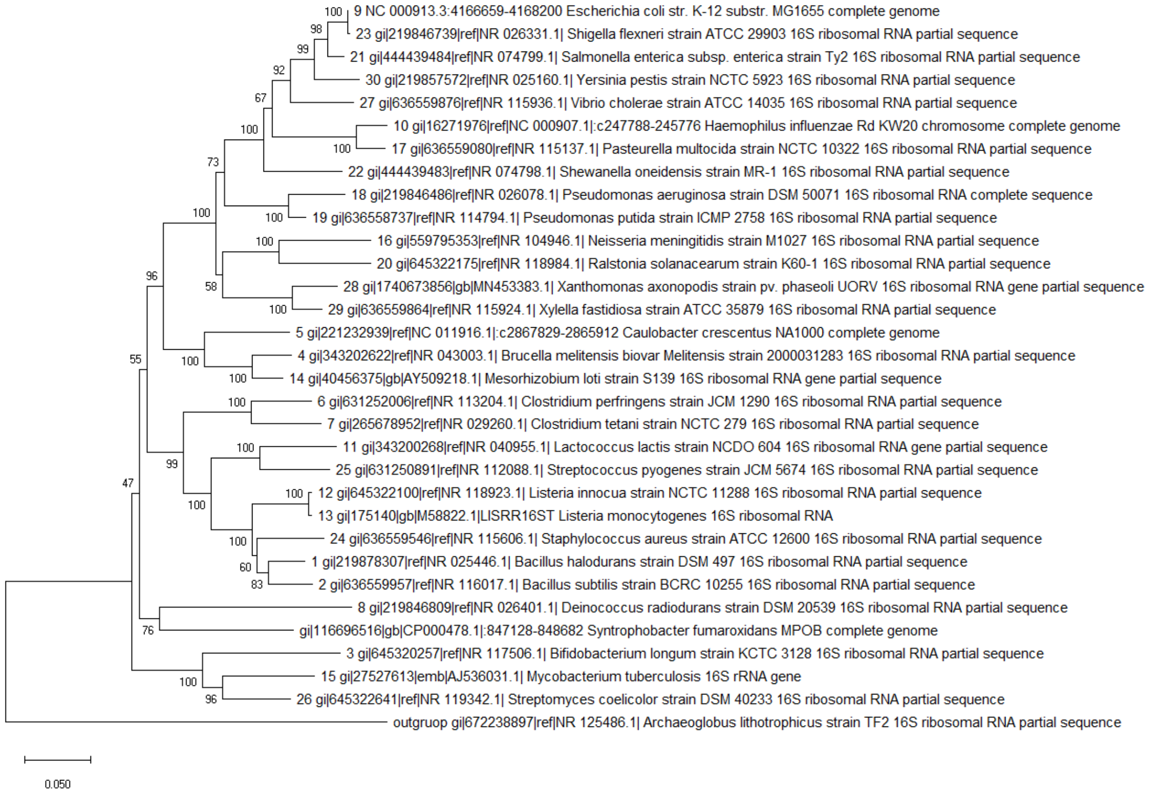

Figure S1.79: Phylogenetic tree of 16S rRNA gene sequences of *Syntrophobacter fumaroxidans* MPOB and microbial strains belonging to the training set of PhiSpy.

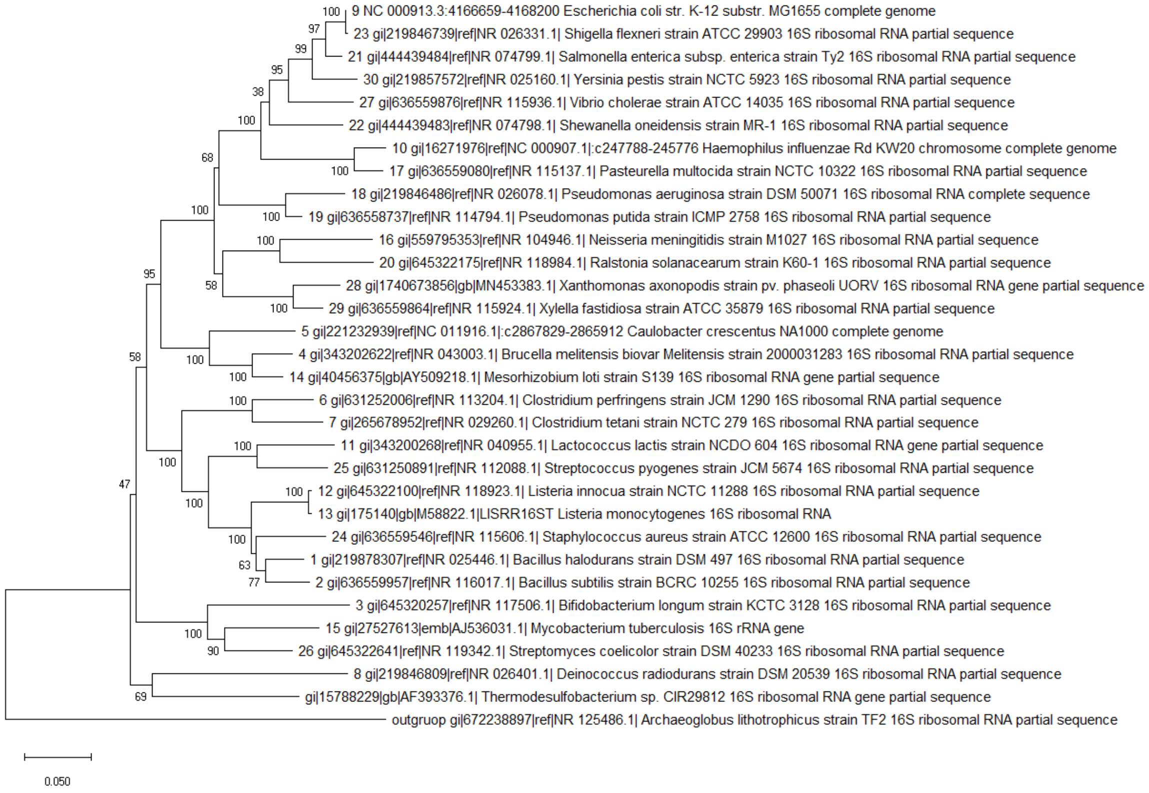

Figure S1.80: Phylogenetic tree of 16S rRNA gene sequences of *Thermodesulfatator indicus* DSM 15286 and microbial strains belonging to the training set of PhiSpy.

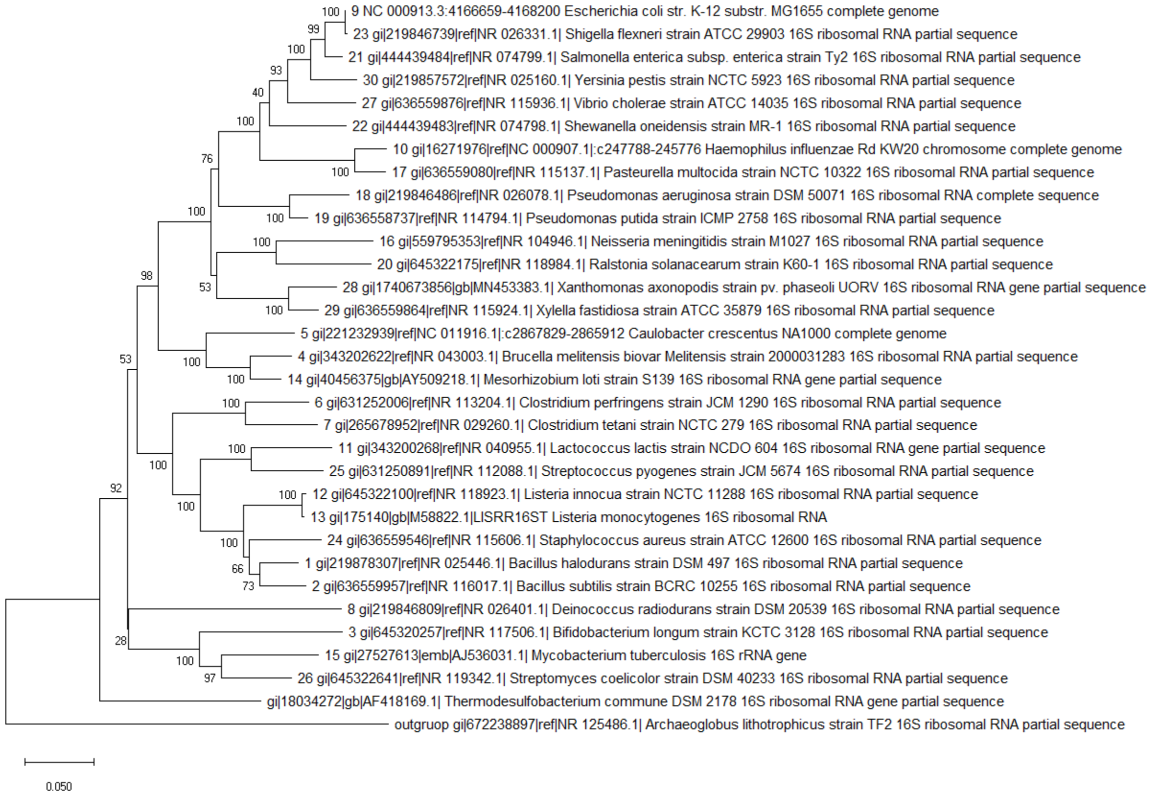

Figure S1.81: Phylogenetic tree of 16S rRNA gene sequences of *Thermodesulfobacterium commune* DSM 2178 and microbial strains belonging to the training set of PhiSpy.

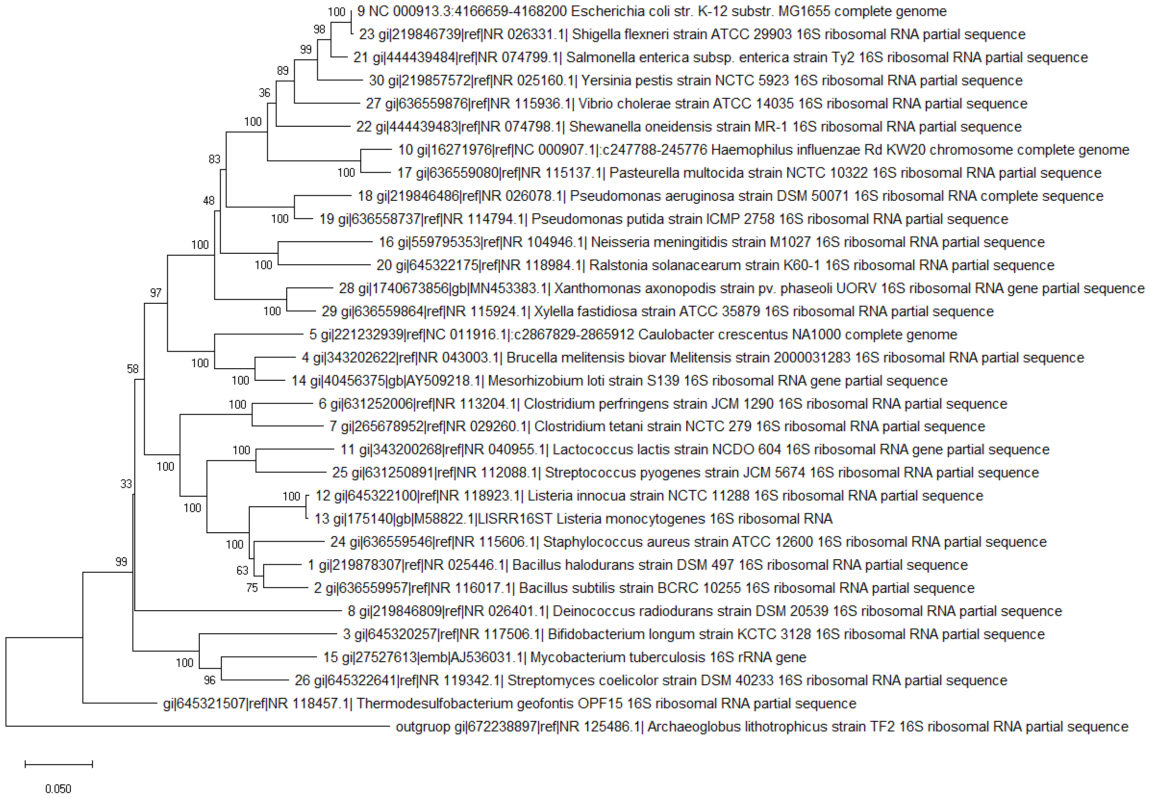

Figure S1.82: Phylogenetic tree of 16S rRNA gene sequences of *Thermodesulfobacterium geofontis* OPF15 and microbial strains belonging to the training set of PhiSpy.

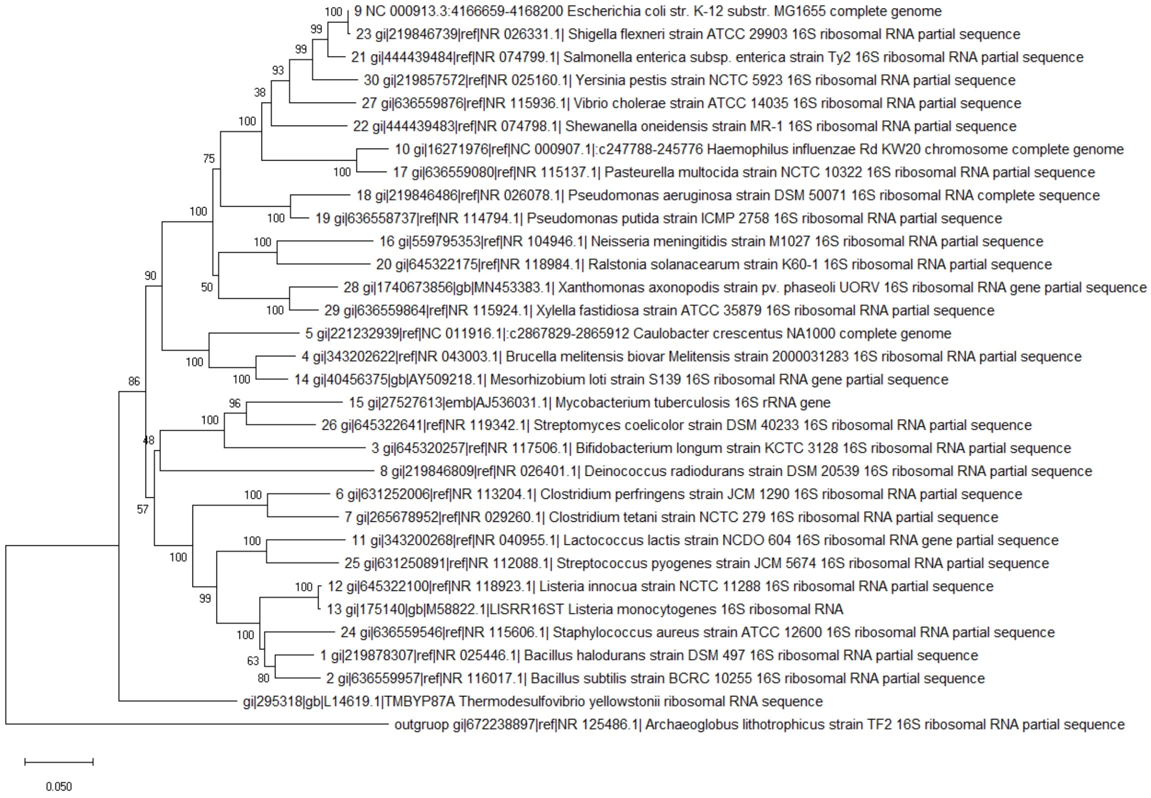

Figure S1.83: Phylogenetic tree of 16S rRNA gene sequences of *Thermodesulfovibrio yellowstonii* and microbial strains belonging to the training set of PhiSpy.

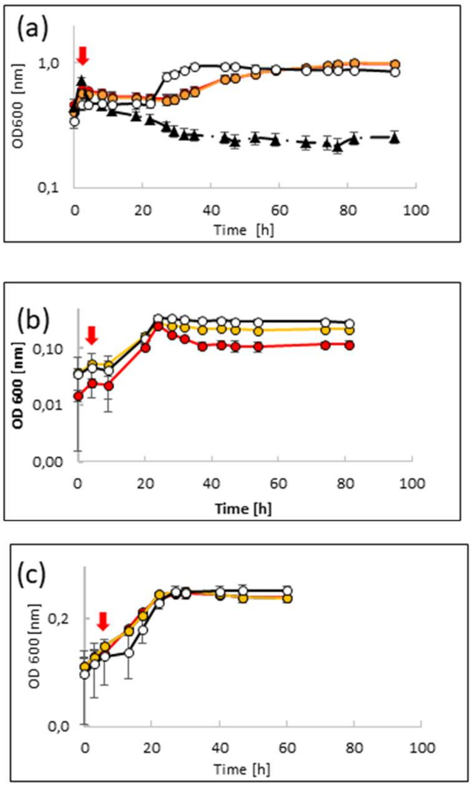

Strain	Prophage	CRISPR
D. vulgaris str. Hildenborough	+++	+
D. africanus DSM 2603	+++	-
D. acrylicus DSM 10141	-	-

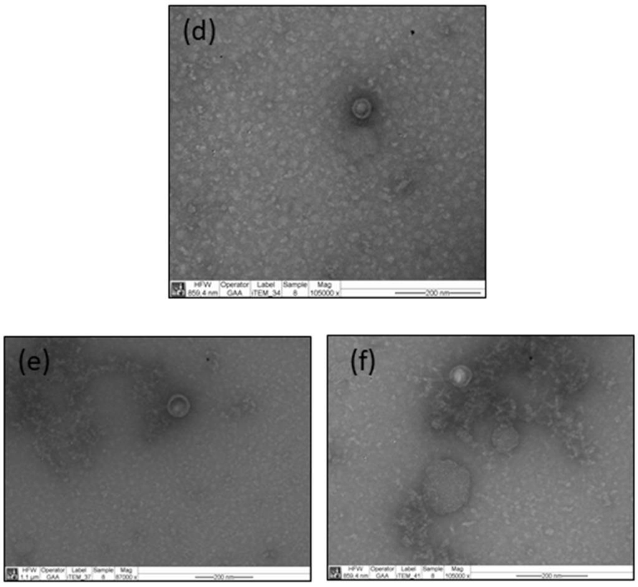

Figure S2: Effect of Mitomycin C on the growth of (a) *D. vulgaris* str. Hildenborough, (b) *D. africanus* 2603 and (c) *D. acrylicus* DSM 10141. White circles indicate cultures grown in the absence of Mitomycin C; orange circles, cultures grown in the presence of 1 µg ml^{-1} of Mitomycin C; red circles, cultures grown in the presence of 3.5 µg ml^{-1} of Mitomycin C. In (a), black triangles indicate cultures grown in the presence of 10 µg ml^{-1} of Mitomycin C. Data represent the mean±sd from three independent replicate cultures. Bars designate one standard deviation of the mean. The table in the upper right section indicate presence (+) or absence (-) of prophages and CRISPR. (d) TEM micrographs of viral-like particles with typical morphotype of *Podoviridae* were obtained from *D. vulgaris* str. Hildenborough batch cultures after induction with Mitomycin C. (e-f) TEM micrographs presents structures that resembles outer membrane vesicles previously observed in *D. alaskensis* DSM16109. Most of the micrographs obtained in this study contained low-resolution viral particles and structures, cell debris, and a noisy background as a result of the interactions of the compounds of the growth medium of SRP.

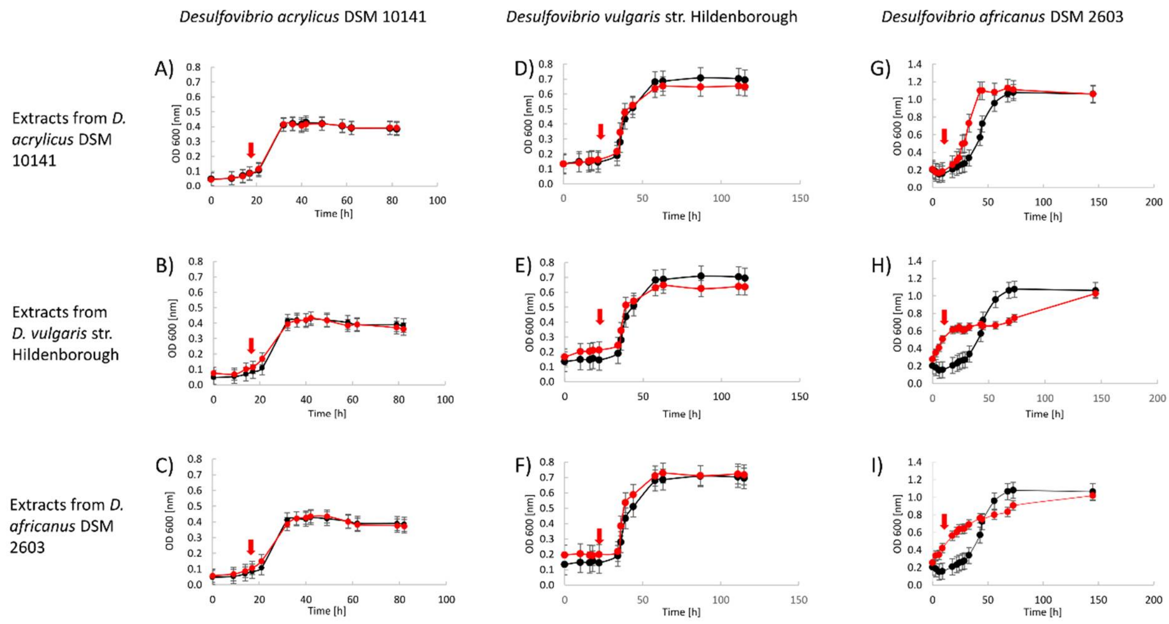

Figure S3A: Effect of filtrates from mitomycin induced SRB cultures added at early exponential phase on the growth of *Desulfovibrio acrylicus* DSM 10141 (A-C), *Desulfovibrio vulgaris* str. Hildenborough (D-F), and *Desulfovibrio africanus* DSM 2603 (G-I). Black and red circles indicate cultures grown in the absence and presence of filtrates. Data represent the mean±sd from three independent replicate cultures. Bars designate one standard deviation of the mean.

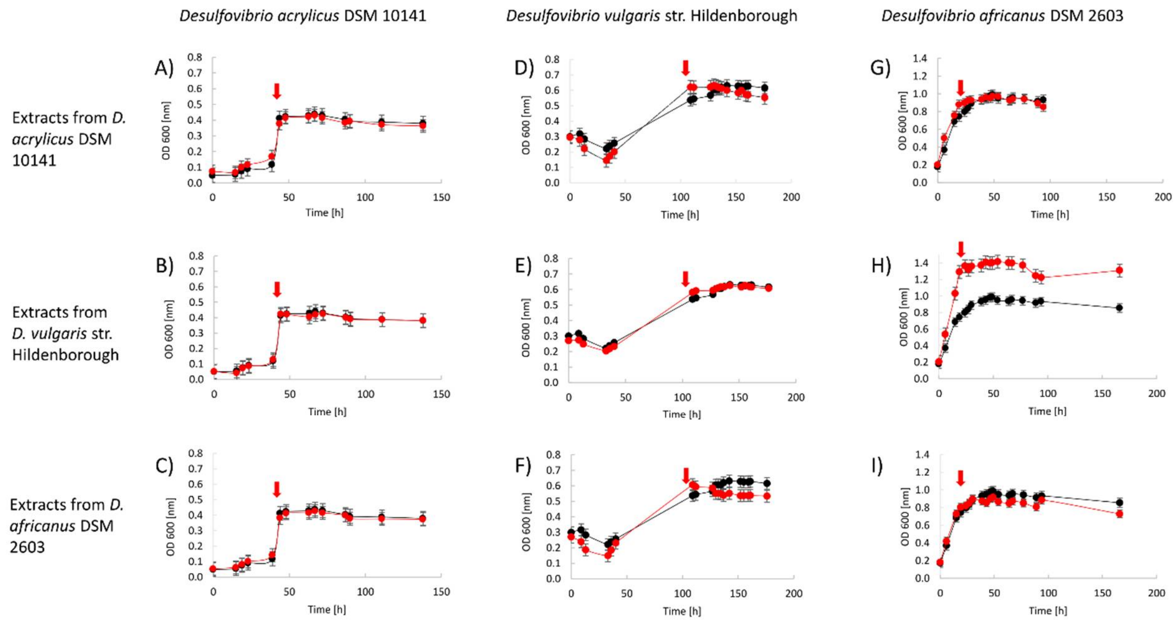

Figure S3B: Effect of filtrates from mitomycin induced SRB cultures added at early stationary phase on the growth of *Desulfovibrio acrylicus* DSM 10141 (A-C), *Desulfovibrio vulgaris* str. Hildenborough (D-F), and *Desulfovibrio africanus* DSM 2603 (G-I). Black and red circles indicate cultures grown in the absence and presence of filtrates. Data represent the mean±sd from three independent replicate cultures. Bars designate one standard deviation of the mean.

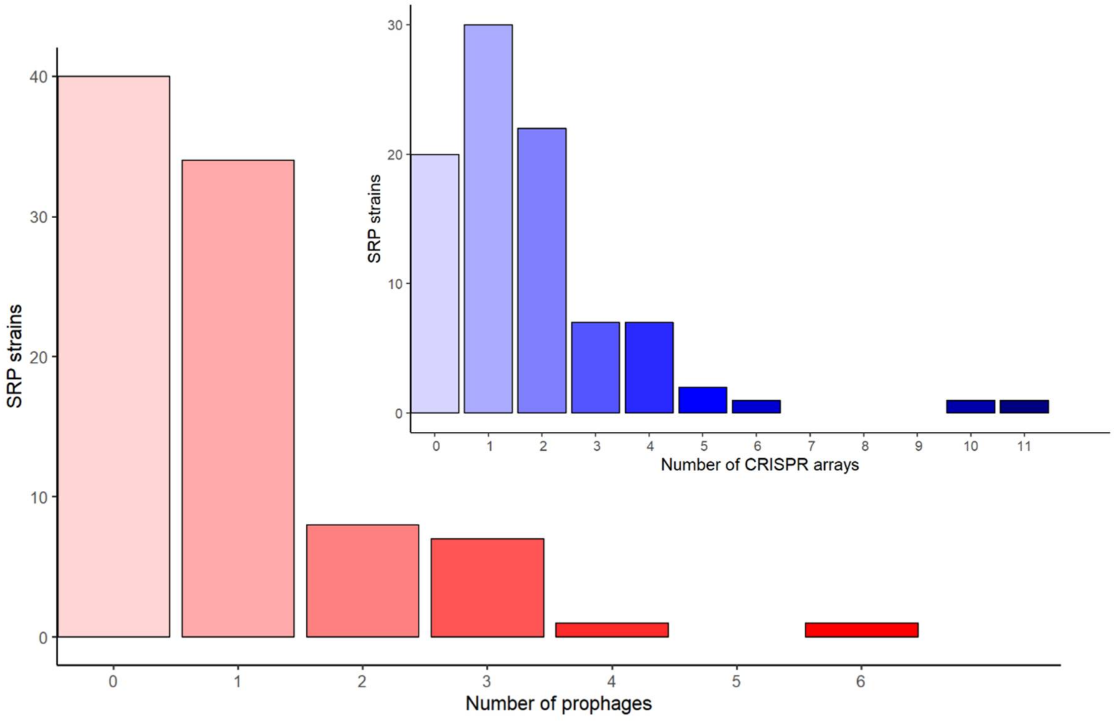

Figure S4: Frequencies of integrated prophages (light red) and CRISPR arrays (light blue) in the genomes of SRP strains.

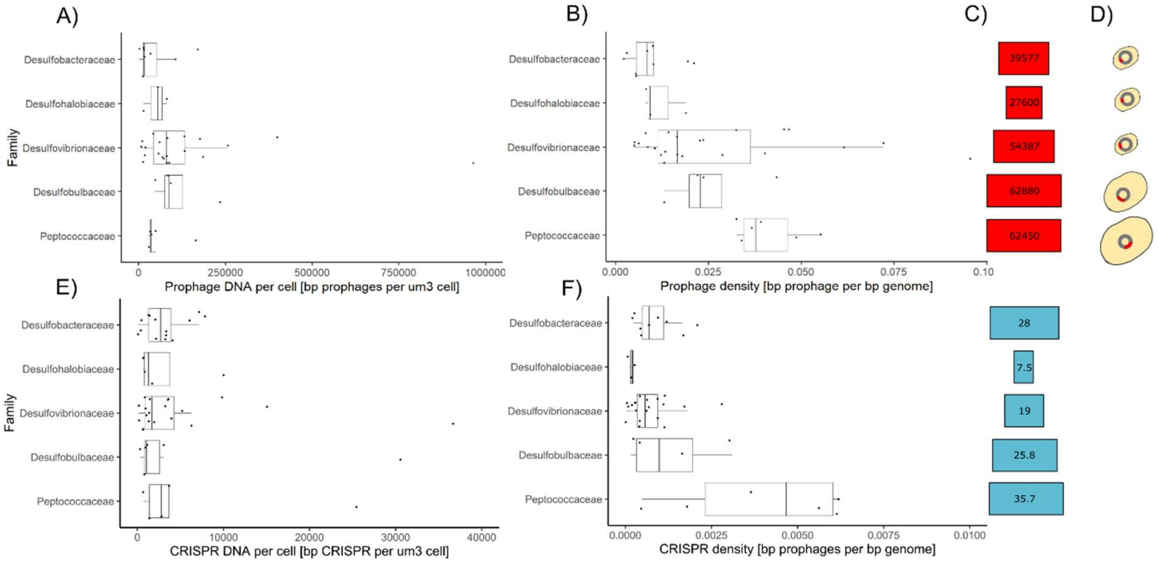

Figure S5: Box-plots of (A) DNA prophage per cell (volume) and (B) prophage density of lysogens belonging to five families of SRP; (C) red bars indicate the median of the length of prophages and (D) represents the median cellular volume and median genome size found in each family. Box-plots of (E) CRISPR DNA per cell and (F) CRISPR density of CRISPR-carrying strains belonging to five families of SRP; (G) light blue bars indicate the median of number of spacers belonging to strains of each family.

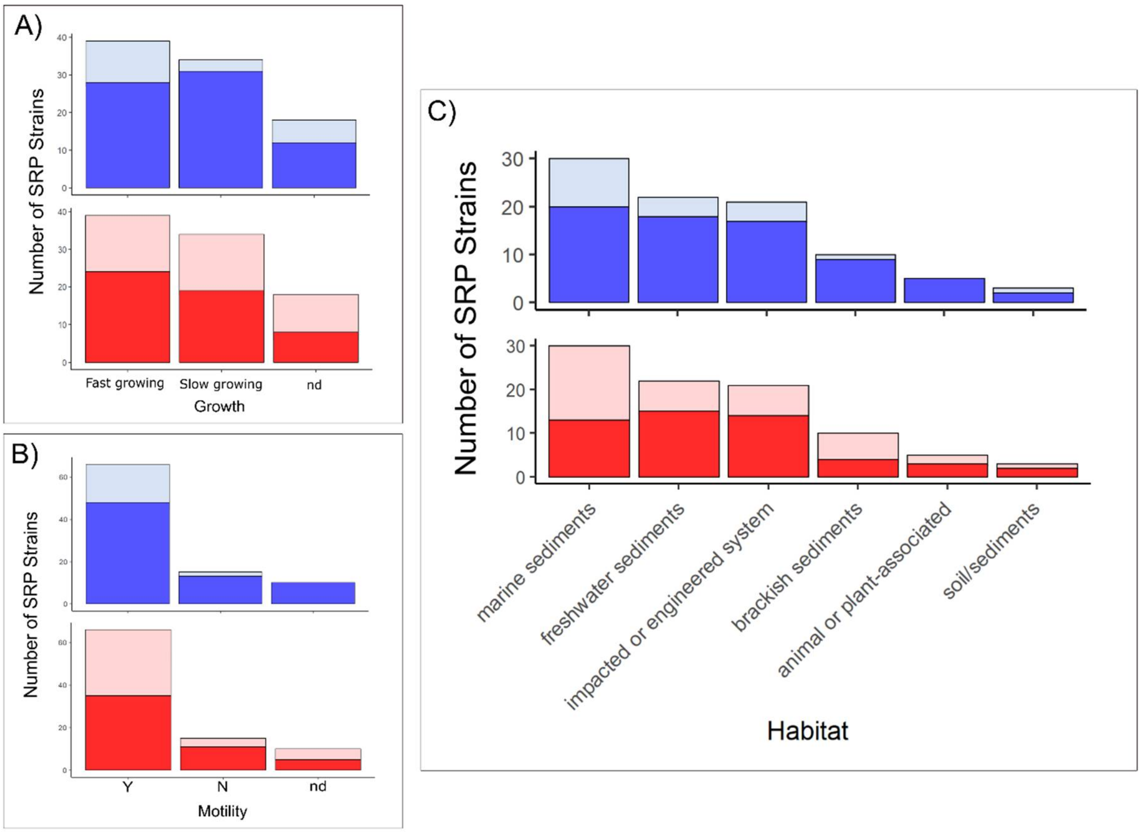

Figure S6: Distribution of prophages (red) and CRISPR (blue) according to different ecophysiological traits. In the left top (A), it is shown the distribution of prophages and CRISPR arrays according to the type of growth (doubling time ≤ 12 hours or > 12 hours, as fast-growing or slow-growing strains, respectively). In the left bottom (B), it is shown the distribution of prophages and CRISPR arrays according to the presence of flagellum, classified as strains with flagellum (Y), strains for which flagellum has not been observed (N) and strains with no information (nd). In the right (C), it is shown the distribution of prophages and CRISPR arrays according to type of environment in which strains were isolated or the source of material that allowed the reconstruction of the genomes.

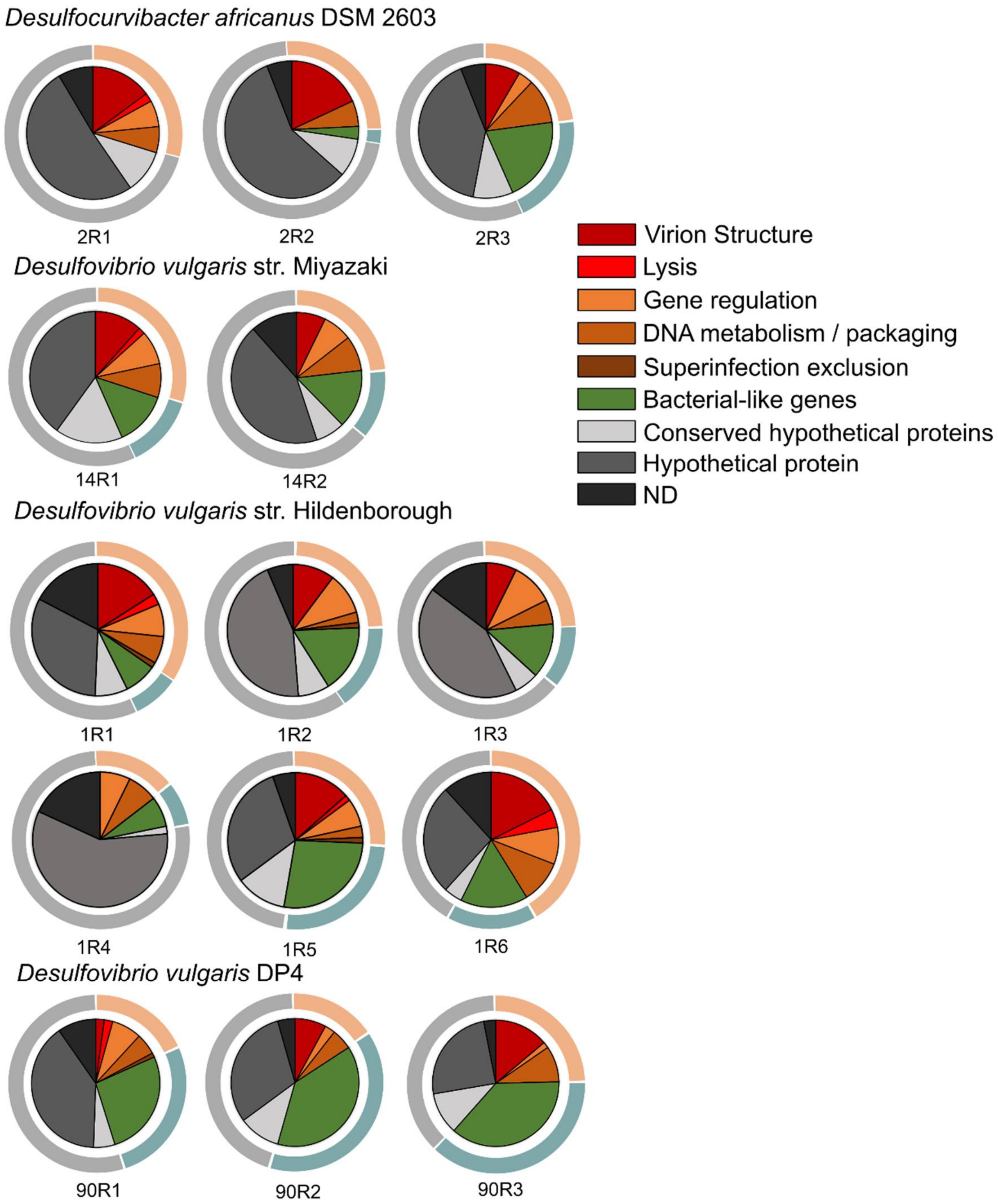

Figure S7: Comparison of genome annotations among SRP prophages. Pie charts indicate the annotation of prophages from *Desulfocurvibacter africanus* 2603, *Desulfovibrio vulgaris* str. Miyazaki, *Desulfovibrio vulgaris* str. Hildenborough and *Desulfovibrio vulgaris* DP4. The inner circle indicates functional classification (see color bar) and outer circle indicates the classification in the following categories; genes with bacterial origin (light green), genes with viral origin (light orange) and genes with unknown function (light gray).

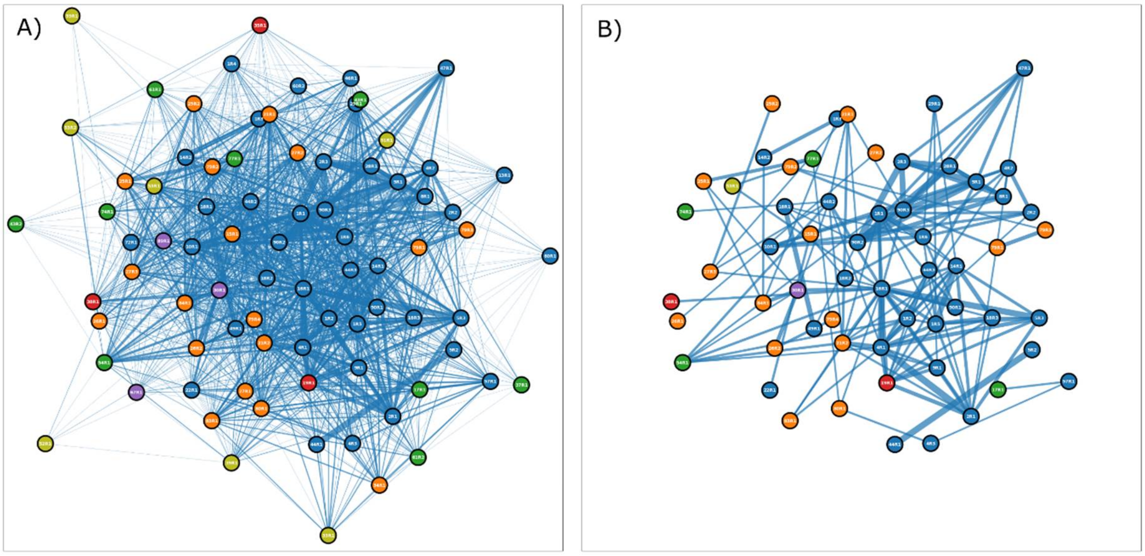

Figure S8: (A) Network shows the degree of homology among SRP prophages. Circles indicate each SRP, colored by the taxonomy of the host. Edges show the presence of homologous genes among prophages. (B) Network shows the degree of homology among SRP prophages that share more than 10 homologs. The width of edges indicates the number of homologous genes between two prophages.

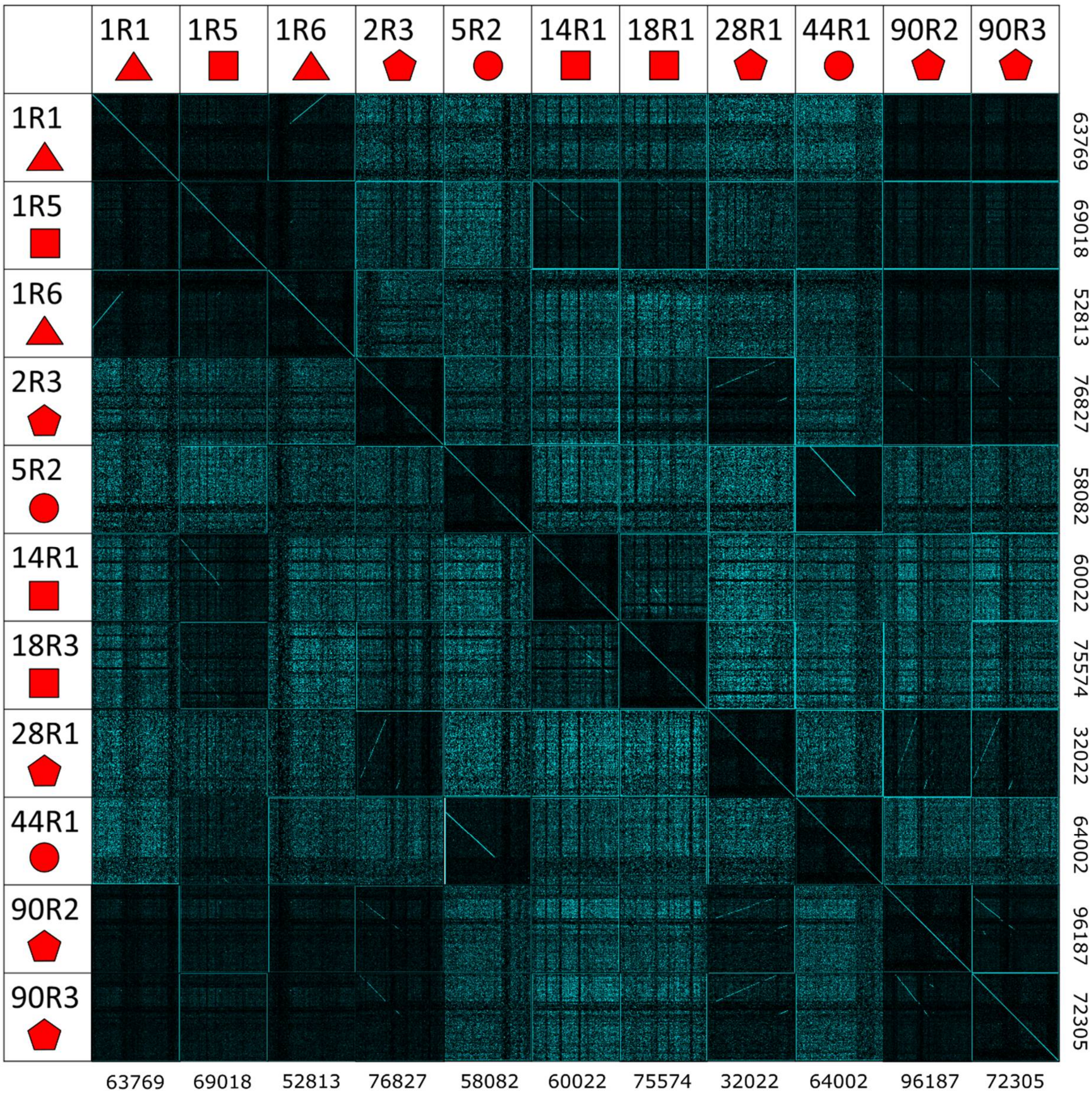

Figure S9: Whole-genome dot plot comparison of prophage nucleotides sequences of *Desulfovibrio* strains, prophages 1R1, 1R5 and 1R6 of *D. vulgaris* Hildenborough, 2R3 of *Desulfovibrio africanus* DSM 260, 5R2 of *Desulfovibrio aespoeensis* Aspo-2, 14R1 of *Desulfovibrio vulgaris* str. Miyazaki, 18R1 of *Desulfovibrio fructosivorans* JJ, 28R1 of *Desulfovibrio* sp. X2, 44R1 of *Desulfovibrio alaskensis* G20, and 90R2 and 90R3 of *Desulfovibrio vulgaris* DP4. Lack of grouping in dot plots are both reflection of the overall high degree of diversity and low level of synteny of the SRP prophages. The symbols indicate the classification of each prophage according to VIRFAM. The numbers in the right and bottom indicate the length of each prophage (in bp).

No	SRP Strain	Name	ID	CRISPR (2-4) found by CRISPRCasFinder	Putative CRISPR found by CRISPRCasFinder	Number of prophage like elements
1	*Desulfovibrio vulgaris* str. Hildenborough	plasmid pDV	NC_005863.1	1	0	0
7	*Desulfovibrio magneticus* RS-1	plasmid pDMC1	NC_012797.1	0	0	0
		plasmid pDMC2	NC_012795.1			
9	*Desulfovibrio gigas* DSM 1382	plasmid unnamed	NC_022436.1	0	0	0
24	*Desulfovibrio hydrothermalis* DSM 14728	plasmid DESAM_p	NC_019953.1	0	0	0
30	*Desulfosporosinus acidiphilus* SJ4	plasmid pDESACI.02	CP003641.1	0	0	0
		plasmid pDESACI.01	CP003640.1			
38	*Desulfohalobium retbaense*	plasmid pDRET01	NC_013224.1	0	0	0
40	*Desulfobacterium autotrophicum* HRM2	plasmid pHRM2a	NC_012109.1	0	0	0
43	*Desulfotalea psychrophila* LSv54	small plasmid	CR522872.1	0	2	0
		large plasmid	CR522871.1			
46	*Desulfovibrio* sp. FW1012B	plasmid pFW10102	NZ_CM001370.1	0	0	0
		plasmid pFW10101	NZ_CM001369_1			
67	*Desulfurella acetivorans* DSM 5264	plasmid unnamed	NZ_CP007052.1	0	0	0
76	*Desulfocapsa sulfexigens* DSM 10523	plasmid unnamed	NC_020305.1	0	0	0
86	*Archaeoglobus profundus* DSM 5631	plasmid pArcpr01	NC_013742.1	0	0	0
90	*Desulfovibrio vulgaris* DP4	plasmid pDVUL01	NC_008741.1	1	0	0

Table S1: CRISPR and putative CRISPR arrays identified in plasmids of SRP analyzed in this study.

Number	Strain name	Number of prophage	Prophage ID	Prophage Sequence ID	Start	End	length (bp)	Number of ORFs	Total prophage length (bp)	Avg prophage length (bp)	Genome Size (bp)	Prophage Density (%)	Total Prophages Density	Taxonomic classification according to VIRFAM
1	Desulfovibrio vulgaris str. Hildenborough	6	1R1	|1R1|gi|46562128|ref|NC_002937.3|Complete Genome|239651|303420	239651	303420	63769	75	360426	60071	3773160	1.69%	9.55%	Myoviridae of Type1 (Cluster 6)
			1R2	|1R2|gi|46562128|ref|NC_002937.3|Complete Genome|1205411|1277056	1205411	1277056	71645	78				1.90%		Myoviridae of Type1 (Cluster 8)
			1R3	|1R3|gi|46562128|ref|NC_002937.3|Complete Genome|1561263|1625178	1561263	1625178	63915	68				1.69%		Siphoviridae of Type1 (cluster 6)
			1R4	|1R4|gi|46562128|ref|NC_002937.3|Complete Genome|1783411|1822677	1783411	1822677	39266	55				1.04%		Podoviridae of Type3
			1R5	|1R5|gi|46562128|ref|NC_002937.3|Complete Genome|2801325|2870343	2801325	2870343	69018	74				1.83%		Myoviridae of Type1 (Cluster 8)
			1R6	|1R6|gi|46562128|ref|NC_002937.3|Complete Genome|2936361|2989174	2936361	2989174	52813	68				1.40%		Myoviridae of Type1 (Cluster 6)
2	Desulfocurvibacter africanus DSM 2603	3	2R1	|2R1|gi|523389900|gb|AULZ01000005.1|H585DRAFT_scaffold00004.4_C|86038|118974	86038	118974	32936	47	138181	46060	4400410	0.75%	3.14%	Myoviridae of Type1 (Cluster 8)
			2R2	|2R2|gi|523389844|gb|AULZ01000010.1|H585DRAFT_scaffold00008.8_C|45240|73658	45240	73658	28418	33				0.65%		Myoviridae of Type1 (Cluster 6)
			2R3	|2R3|gi|523389782|gb|AULZ01000012.1|H585DRAFT_scaffold00010.10_C|2130|78957	2130	78957	76827	83				1.75%		Myoviridae of Type1 (Cluster 9)

3	Desulfovibrio desulfuricans subsp. aestuarii ATCC 29578	0									3473760			
4	Desulfocurvibacter africanus str. Walvis Bay	3	4R1	|4R1|gi|374298386|ref|NC_016629.1|Complete Genome|727631|787504	727631	787504	59873	70	121145	40382	4200530	1.43%	2.88%	Myoviridae of Type1 (Cluster 8)
			4R2	|4R2|gi|374298386|ref|NC_016629.1|Complete Genome|2137665|2174427	2137665	2174427	36762	51				0.88%		Myoviridae of Type1 (Cluster 6)
			4R3	|4R3|gi|374298386|ref|NC_016629.1|Complete Genome|3907176|3931686	3907176	3931686	24510	26				0.58%		Myoviridae
5	Desulfovibrio aespoeensis Aspo-2	3	5R1	|5R1|gi|317151727|ref|NC_014844.1|Complete Genome|460133|530699	460133	530699	70566	102	164722	54907	3629110	1.94%	4.54%	Myoviridae of Type1 (Cluster 9)
			5R2	|5R2|gi|317151727|ref|NC_014844.1|Complete Genome|1788329|1846411	1788329	1846411	58082	55				1.60%		Myoviridae
			5R3	|5R3|gi|317151727|ref|NC_014844.1|Complete Genome|2595084|2631158	2595084	2631158	36074	52				0.99%		Myoviridae of Type1 (Cluster 8)
6	Desulfovibrio acrylicus DSM 10141	0									3401710			
7	Desulfovibrio magneticus RS-1	0									4844650			
8	Desulfovibrio desulfuricans subsp. desulfuricans str. ATCC 27774	1	8R1	|8R1|gi|220903286|ref|NC_011883.1|Complete Genome|271014|318358	271014	318358	47344	55	47344	47344	2873437	1.65%	1.65%	Myoviridae of Type1 (Cluster 9)
9	Desulfovibrio gigas DSM 1382	1	9R1	|9R1|gi|545640361|ref|NC_022444.1|Complete Genome|992550|1046937	992550	1046937	54387	69	54387	54387	3792120	1.43%	1.43%	Siphoviridae of Type1 (Cluster 8)
10	Desulfovibrio piger ATCC 29098	1	10R1	|10R1|gi|209947761|gb|ABXU01000026.1|1.0_Cont4.1|16558|54731	16558	54731	38173	51	38173	38173	2867220	1.33%	1.33%	Myoviridae of Type1 (Cluster 6)

11	Desulfovibrio cf. magneticus IFRC170	0									4844650			
12	Desulfovibrio oxyclinae DSM 11498	0									3324580			
13	Desulfovibrio cuneatus DSM 11391	1	13R1	|13R1|gi|523621182|gb|AUCY01000006.1|G497DRAFT_scaffold00003.3_C2|20537|76517	20537	76517	55980	50	55980	55980	3359130	1.67%	1.67%	Myoviridae
14	Desulfovibrio vulgaris str. Miyazaki	2	14R1	|14R1|gi|218885109|ref|NC_011769.1|Complete Genome|862570|922592	862570	922592	60022	60	121853	60927	3753760	1.60%	3.25%	Myoviridae of Type1 (Cluster 8)
			14R2	|14R2|gi|218885109|ref|NC_011769.1|Complete Genome|2016944|2078775	2016944	2078775	61831	69				1.65%		Siphoviridae of Type1 (Cluster 3)
15	Desulfobulbus elongatus DSM 2908	1	15R1	|15R1|gi|607834641|gb|JHZB01000015.1|Q362DRAFT_scaffold00014.14_C|78|51849	78	51849	51771	78	51771	51771	3961950	1.31%	1.31%	Myoviridae of Type1 (Cluster 6)
16	Desulfocurvus vexinensis DSM 17965	1	16R1	|16R1|gi|573088796|gb|JAEX01000002.1|WGS:JAEX01:G495DRAFT_scaffold00002.2_C|111939|257727	111939	257727	145788	142	145788	145788	3627180	4.02%	4.02%	Myoviridae of Type1 (Cluster 8)
17	Desulfovibrio alkalitolerans	1	17R1	|17R1|gi|523463601|gb|ATHI01000003.1|ctg12|31783|65856	31783	65856	34073	44	34073	34073	3202330	1.06%	1.06%	Myoviridae of Type1 (Cluster 8)
18	Desulfovibrio fructosivorans JJ	3	18R1	|18R1|gi|302493090|gb|AECZ01000001.1|ctg00049|320|75894	320	75894	75574	83	218120	72707	4673590	1.62%	4.67%	Siphoviridae of Type1 (No Cluster Assigned)
			18R2	|18R2|gi|302493090|gb|AECZ01000001.1|ctg00049|146136|239339	146136	239339	93203	95				1.99%		Siphoviridae of Type1 (Cluster 8)
			18R3	|18R3|gi|302492071|gb|AECZ01000006.1|ctg00055|46365|95708	46365	95708	49343	61				1.06%		Myoviridae of Type1 (Cluster 8)
19	Desulfonatronovibrio hydrogenovorans DSM 9292	1	19R1	|19R1|gi|631913846|gb|JMKT01000009.1|P771DRAFT_scaffold00002.2_C|55099|82699	55099	82699	27600	70	27600	27600	2939700	0.94%	0.94%	Myoviridae of Type1 (Cluster 8)

<table>
<tr><td>20</td><td>Desulfobulbaceae bacterium BRH_c16a</td><td>0</td><td></td><td></td><td></td><td></td><td></td><td></td><td></td><td></td><td>6515850</td><td></td><td></td><td></td></tr>
<tr><td rowspan="2">21</td><td rowspan="2">Desulfobulbus propionicus type strain 1pr3</td><td rowspan="2">2</td><td>21R1</td><td>|21R1|gi|320352146|ref|NC_014972.1|Complete Genome|179310|259153</td><td>179310</td><td>259153</td><td>79843</td><td>93</td><td rowspan="2">166855</td><td rowspan="2">83428</td><td rowspan="2">3851870</td><td>2.07%</td><td rowspan="2">4.33%</td><td>Siphoviridae of Type1 (Cluster 6)</td></tr>
<tr><td>21R2</td><td>|21R2|gi|320352146|ref|NC_014972.1|Complete Genome|2768665|2855677</td><td>2768665</td><td>2855677</td><td>87012</td><td>96</td><td>2.26%</td><td>Siphoviridae of Type1 (Cluster 8)</td></tr>
<tr><td>22</td><td>Desulfovibrio salexigens</td><td>1</td><td>22R1</td><td>|22R1|gi|242277482|ref|NC_012881.1|Complete Genome|2324343|2361506</td><td>2324343</td><td>2361506</td><td>37163</td><td>51</td><td>37163</td><td>37163</td><td>4289850</td><td>0.87%</td><td>0.87%</td><td>Myoviridae of Type1 (Cluster 6)</td></tr>
<tr><td>23</td><td>Desulfovibrio frigidus DSM 17176</td><td>0</td><td></td><td></td><td></td><td></td><td></td><td></td><td></td><td></td><td>4185040</td><td></td><td></td><td></td></tr>
<tr><td>24</td><td>Desulfovibrio hydrothermalis DSM 14728</td><td>0</td><td></td><td></td><td></td><td></td><td></td><td></td><td></td><td></td><td>3683790</td><td></td><td></td><td></td></tr>
<tr><td rowspan="2">25</td><td rowspan="2">Desulfotomaculum hydrothermale Lam5</td><td rowspan="2">2</td><td>25R1</td><td>|25R1|gi|411455801|emb|CAOS01000001.1|contig ABX_2590_1|30068|75464</td><td>30068</td><td>75464</td><td>45396</td><td>53</td><td rowspan="2">105398</td><td rowspan="2">52699</td><td rowspan="2">2695030</td><td>1.68%</td><td rowspan="2">3.91%</td><td>Siphoviridae of Type1 (Cluster 2)</td></tr>
<tr><td>25R2</td><td>|25R2|gi|411454438|emb|CAOS01000009.1|contig ABX_2590_1|77813|137815</td><td>77813</td><td>137815</td><td>60002</td><td>87</td><td>2.23%</td><td>Siphoviridae of Type1 (Cluster 2)</td></tr>
<tr><td rowspan="2">26</td><td rowspan="2">Desulfotomaculum ruminis DSM 2154</td><td rowspan="2">2</td><td>26R1</td><td>|26R1|gi|334089967|gb|CP002780.1|Complete Genome|1102652|1163694</td><td>1102652</td><td>1163694</td><td>61042</td><td>66</td><td rowspan="2">145572</td><td rowspan="2">72786</td><td rowspan="2">3969010</td><td>1.54%</td><td rowspan="2">3.67%</td><td>Podoviridae</td></tr>
<tr><td>26R2</td><td>|26R2|gi|334089967|gb|CP002780.1|Complete Genome|3519929|3604459</td><td>3519929</td><td>3604459</td><td>84530</td><td>88</td><td>2.13%</td><td>Myoviridae of Type1 (Cluster 2)</td></tr>
<tr><td rowspan="2">27</td><td rowspan="2">Desulfotomaculum reducens MI-1</td><td rowspan="2">3</td><td>27R1</td><td>|27R1|gi|134050581|gb|CP000612.1|Complete Genome|532811|592446</td><td>532811</td><td>592446</td><td>59635</td><td>64</td><td rowspan="2">175852</td><td rowspan="2">58617</td><td rowspan="2">3608100</td><td>1.65%</td><td rowspan="2">4.87%</td><td>unclassified</td></tr>
<tr><td>27R2</td><td>|27R2|gi|134050581|gb|CP000612.1|Complete</td><td>1258679</td><td>1310519</td><td>51840</td><td>69</td><td>1.44%</td><td>Myoviridae of Type1 (Cluster 6)</td></tr>
</table>

				Genome|1258679|1310519										
			27R3	|27R3|gi|134050581|gb|CP000612.1|Complete Genome|2816410|2880787	2816410	2880787	64377	79				1.78%		Siphoviridae of Type1 (Cluster 2)
28	Desulfovibrio sp. X2	1	28R1	|28R1|gi|523471277|gb|ATHV01000018.1|ctg26|48753|80775	48753	80775	32022	49	32022	32022	3966570	0.81%	0.81%	Myoviridae of Type1 (Cluster 9)
29	Desulfovibrio sp. A2	1	29R1	|29R1|gi|347519856|gb|AGFG01000018.1|contig.17|2|24810	2	24810	24808	26	24808	24808	3966570	0.63%	0.63%	Myoviridae
30	Desulfosporosinus acidiphilus SJ4	1	30R1	|30R1|gi|391353422|gb|CP003639.1|1337287|1506880	1337287	1506880	169593	207	169593	169593	4991180	3.40%	3.40%	Myoviridae of Type1 (Cluster 6)
31	Desulfovibrio aminophilus DSM 12254	0									3419060			
32	Desulfobacter curvatus DSM 3379	0									5644280			
33	Desulfobacter vibrioformis DSM 8776	1	33R1	|33R1|gi|675284508|gb|JQKJ01000011.1|Q366DRAFT_scaffold00010.10_C|137688|162790	137688	162790	25102	35	25102	25102	4466780	0.56%	0.56%	Siphoviridae of Type1 (Cluster 8)
34	Desulfobacterium anilini DSM 4660	1	34R1	|34R1|gi|523385977|gb|AULM01000002.1|H567DRAFT_scaffold00002.2_C|335738|382795	335738	382795	47057	49	47057	47057	4667340	1.01%	1.01%	Siphoviridae of Type1 (Cluster 8)
35	Desulfovermiculus halophilus DSM 18834	1	35R1	|35R1|gi|607833964|gb|JIAK01000030.1|N902DRAFT_scaffold00028.28_C|221|26613	221	26613	26392	32	26392	26392	3242090	0.81%	0.81%	unclassified
36	Desulfovibrio piezophilus C1TLV30	0									3646100			
37	Desulfomicrobium baculatum DSM 4028	1	37R1	|37R1|gi|256827818|ref|NC_013173.1|Complete Genome|1362708|1399475	1362708	1399475	36767	42	36767	36767	3942660	0.93%	0.93%	unclassified

38	Desulfohalobium retbaense	1	38R1	|38R1|gi|258404138|ref|NC_013223.1|Complete Genome|1646202|1701330	1646202	1701330	55128	61	55128	55128	2909570	1.89%	1.89%	unclassified
39	Desulfatibacillum alkenivorans AK-01	1	39R1	|39R1|gi|218777860|ref|NC_011768.1|Complete Genome|4517116|4553239	4517116	4553239	36123	30	36123	36123	6517070	0.55%	0.55%	unclassified
40	Desulfobacterium autotrophicum HRM2	0									5657780			
41	Desulfobacula sp. TS	0									2803910			
42	Desulfobacula toluolica Tol2	0									5197910			
43	Desulfotalea psychrophila LSv54	2	43R1	|43R1|gi|51243852|ref|NC_006138.1|Complete Genome|1742755|1794946	1742755	1794946	52191	55	81001	40501	3659630	1.43%	2.21%	Siphoviridae
			43R2	|43R2|gi|51243852|ref|NC_006138.1|Complete Genome|2277660|2306470	2277660	2306470	28810	33				0.79%		Podoviridae of Type3
44	Desulfovibrio alaskensis G20	3	44R1	|44R1|gi|78355047|ref|NC_007519.1|Complete Genome|940169|1004171	940169	1004171	64002	65	224308	74769	3642100	1.76%	6.16%	Myoviridae
			44R2	|44R2|gi|78355047|ref|NC_007519.1|Complete Genome|2753210|2863858	2753210	2863858	110648	98				3.04%		unclassified
			44R3	|44R3|gi|78355047|ref|NC_007519.1|Complete Genome|3322987|3372645	3322987	3372645	49658	65				1.36%		Myoviridae of Type1 (Cluster 8)
45	Desulforegula conservatrix Mb1Pa	0									4469040			
46	Desulfovibrio sp. FW1012B	1	46R1	|46R1|gi|357632130|ref|NZ_CM001368.1|Complete	3969968	4041255	71287	107	71287	71287	3966570	1.80%	1.80%	Siphoviridae of Type1 (Cluster 3)

				Genome|3969968|4041255										
47	Desulfovibrio bastinii DSM 16055	1	47R1	|47R1|gi|523620924|gb|AUCX01000031.1|G496DRAFT_scaffold00028.28_C|734|21301	734	21301	20567	30	20567	20567	3909170	0.53%	0.53%	Myoviridae
48	Desulfonatronospira thiodismutans	0									4107390			
49	Desulfovibrio alcoholivorans DSM 5433	1	49R1	|49R1|gi|651606221|gb|JNJA01000003.1|Q368DRAFT_scaffold00003.3_C|13924|135240	13924	135240	121316	125	121316	121316	5128910	2.37%	2.37%	Siphoviridae of Type1 (Cluster 5)
50	Desulfovibrio desulfuricans 642	0									3132560			
51	Desulfobacter postgatei 2ac9	1	51R1	|51R1|gi|387144466|gb|AGJR02000005.1|Desposcaffold_1_Cont5|217845|258576	217845	258576	40731	36	40731	40731	3974660	1.02%	1.02%	unclassified
52	Desulfotignum phosphitoxidans DSM 13687	1	52R2	|52R1|gi|474489389|gb|APJX01000002.1|Dpo_2c|129284|145082	129284	145082	15798	18	15798	15798	4998760	0.32%	0.32%	unclassified
53	Desulfotignum balticum DSM 7044	2	53R1	|53R1|gi|522811039|gb|ATWO01000001.1|K365DRAFT_scaffold00001.1_C|1713792|1767040	1713792	1767040	53248	55	108373	54187	5118760	1.04%	2.12%	unclassified
			53R2	|53R2|gi|522811039|gb|ATWO01000001.1|K365DRAFT_scaffold00001.1_C|3158452|3213577	3158452	3213577	55125	68				1.08%		unclassified
54	Desulfatirhabdium butyrativorans DSM 18734	1	54R1	|54R1|gi|523620761|gb|AUCU01000021.1|G492DRAFT_scaffold00016.16_C|22017|60400	22017	60400	38383	39	38383	38383	4481980	0.86%	0.86%	unclassified
55	Desulfobulbus japonicus DSM 18378	0									5800570			
56	Desulfovibrio zosterae DSM 11974	0									4092650			

57	Desulfovibrio putealis DSM 16056	1	57R1	|57R1|gi|523618705|gb|AUBQ01000004.1|G453DRAFT_scaffold00003.3_C|295731|319243	295731	319243	23512	33	23512	23512	4705740	0.50%	0.50%	Myoviridae of Type1 (Cluster 8)
58	Desulfospira joergensenii DSM 10085	0									6119470			
59	Desulfatitalea sp. BRH_c12	1	59R1	|59R1|gi|780825309|gb|LADR01000084.1|BRHa_1003299|256|13379	256	13379	13123	9	13123	13123	5997610	0.22%	0.22%	unclassified
60	Desulfovibrio inopinatus DSM 10711	2	60R1	|60R1|gi|523618871|gb|AUBP01000017.1|G451DRAFT_scaffold00012.12_C|67194|100067	67194	100067	32873	33	70945	35473	5767550	0.57%	1.23%	Podoviridae of Type3
			60R2	|60R2|gi|523618858|gb|AUBP01000024.1|G451DRAFT_scaffold00017.17_C|80515|118587	80515	118587	38072	49				0.66%		Podoviridae of Type3
61	Desulfobacca acetoxidans DSM 11109	2	61R1	|61R1|gi|328951746|ref|NC_015388.1|Complete Genome|629290|671508	629290	671508	42218	58	75188	37594	3282540	1.29%	2.29%	unclassified
			61R2	|61R2|gi|328951746|ref|NC_015388.1|Complete Genome|1673469|1706439	1673469	1706439	32970	33				1.00%		Siphoviridae of Type1 (Cluster 8)
62	Desulfobulbus mediterraneus DSM 13871	0									4796810			
63	Desulfomicrobium escambiense DSM 10707	0									3782110			
64	Desulfonatronum lacustre DSM 10312	0									3761450			
65	Desulfosarcina sp. BuS5	0									3600570			
66	Desulfovibrio longus DSM 6739	0									3701830			
67	Desulfurella acetivorans DSM 5264	1	67R1	|67R1|gi|754092674|ref|NZ_CP007051.1|Complete	1671975	1693619	21644	24	21644	21644	1819990	1.19%	1.19%	unclassified

				Genome|1671975|1693619										
68	Desulfonauticus sp. A7A	0									2014890			
69	Desulfonatronovibrio magnus	0									4809660			
70	Desulfonatronum thioautotrophicum ASO4-1	0									4634600			
71	Desulfonatronum thiodismutans	0									3923650			
72	Desulfovibrio desulfuricans ND132	1	72R1	|72R1|gi|376294792|ref|NC_016803.1|Complete Genome|1652158|1703138	1652158	1703138	50980	42	50980	50980	3858580	1.32%	1.32%	unclassified
73	Desulfococcus multivorans DSM 2059	0									4403010			
74	Desulfococcus oleovorans Hxd3	1	74R1	|74R1|gi|158520017|ref|NC_009943.1|Complete Genome|3444657|3521470	3444657	3521470	76813	75	76813	76813	3944170	1.95%	1.95%	unclassified
75	Desulfobulbus sp. Dsb1 (Tol1)	0									4211780			
76	Desulfocapsa sulfexigens DSM 10523	0									4023510			
77	Desulfurivibrio alkaliphilus AHT 2	1	77R1	|77R1|gi|297567992|ref|NC_014216.1|Complete Genome|2336043|2409612	2336043	2409612	73569	71	73569	73569	3097760	2.37%	2.37%	unclassified
78	Thermodesulfatator indicus DSM 15286	0									2322220			
79	Desulfotomaculum acetoxidans DSM 771	4	79R1	|79R1|gi|257777071|gb|CP001720.1|Complete Genome|1925642|1989115	1925642	1989115	63473	59	251019	62755	4545620	1.40%	5.52%	unclassified
			79R2	|79R2|gi|257777071|gb|CP001720.1|Complete	3847376	3910858	63482	91				1.40%		unclassified

				Genome|3847376|3910858										
			79R3	|79R3|gi|257777071|gb|CP001720.1|Complete Genome|4060951|4122610	4060951	4122610	61659	65				1.36%		unclassified
			79R4	|79R4|gi|257777071|gb|CP001720.1|Complete Genome|4396496|4458901	4396496	4458901	62405	64				1.37%		Myoviridae of Type1 (No Cluster Assigned)
80	Thermodesulfobacterium commune DSM 2178	1	80R1	|80R1|gi|668351138|gb|CP008796.1|Complete Genome|1308664|1372825	1308664	1372825	64161	73	64161	64161	1559460	4.11%	4.11%	Myoviridae of Type1 (Cluster 9)
81	Thermodesulfobacterium geofontis OPF15	0									1634380			
82	Desulfocurvibacter africanus PCS	0									3929487			
83	Syntrophobacter fumaroxidans MPOB	1	83R1	|83R1|gi|116696516|gb|CP000478.1|Complete Genome|2290794|2350190	2290794	2350190	59396	55	59396	59396	4990250	1.19%	1.19%	Siphoviridae of Type1 (Cluster 8)
84	Desulfarculus baarsii DSM 2075	1	84R1	|84R1|gi|301638057|gb|CP002085.1|Complete Genome|2838573|2921972	2838573	2921972	83399	81	83399	83399	3655730	2.28%	2.28%	unclassified
85	Archaeoglobus sulfaticallidus PM70-1	0									2076930			
86	Archaeoglobus profundus DSM 5631	0									1563420			
87	Archaeoglobus fulgidus DSM 4304	0									2107990			
88	Thermodesulfovibrio yellowstonii DSM 11347	0									2003800			
89	Candidatus Desulforudis audaxviator MP104C	1	89R1	|89R1|gi|169637063|gb|CP000860.1|Complete Genome|773247|849934	773247	849934	76687	74	76687	76687	2349480	3.26%	3.26%	unclassified

90	Desulfovibrio vulgaris DP4	3	90R1	|90R1|gi|CP000527.1|gb|NC_008751.1|Complete Genome|1273249|1354897	1273249	1354897	81648	93	250140	83380	3462887	2.36%	7.22%	Siphoviridae of Type1 (Cluster 6)
			90R2	|90R2|gi|CP000527.1|gb|NC_008751.1|Complete Genome|1731933|1828120	1731933	1828120	96187	114				2.78%		Myoviridae of Type1 (Cluster 9)
			90R3	|90R3|gi|CP000527.1|gb|NC_008751.1|Complete Genome|3196647|3268952	3196647	3268952	72305	65				2.09%		Myoviridae of Type1 (Cluster 9)
91	Desulfatibacillum alkenivorans DSM 16219	0									6464641			

Table S2. List of prophages predicted by Phaster and PhiSpy. Columns indicate the following; total number of predicted prophages per host, prophage ID, prophage sequence ID, the start and end of the prophage in the genome, the length of the prophage (bp), the number of ORF, total prophage length (bp), average of the length, genome size of the host, prophage density, total prophage density, and taxonomic classification according to Virfam. Prophage density represents the portion of the host genome that contain each prophage (bp prophage * bp^{-1}host*100).

N	Strain	Range pH	Range T (^{0}C)	Classification	Gram	Motility	Morphology	Type of habitat	Type of growth under optimum conditions	Oxidation	Size (µm^2)	Cell Volume (µm^3)	Volume category	Surface cell area (µm^2)	Reference
1	*Desulfovibrio vulgaris* str. Hildenborough	7	25 - 40	Mesophile	Neg.	Y	Rod	soil/sediments	fast-growing	I	0.8-1.2*2.2-3	2.042	middle-size	9.739	[1,2]
2	*Desulfocurvibacter africanus* DSM 2603	6.6 to 7.65	20 to 40	Mesophile	Neg.	Y	vibrio-shaped	freshwater sediments	fast-growing	I	0.5*5-10	1.472	middle-size	12.173	[3]
3	*Desulfovibrio desulfuricans* subsp. aestuarii ATCC 29578	6.5 to 8.5	10 to 40	Mesophile	Neg.	Y	vibrio-shaped	soil/sediments	fast-growing	I	0.5-0.8*1.5-4	0.885	small	6.172	[4,5]
4	*Desulfocurvibacter africanus* subsp. africanus str. Walvis Bay	7	28 to 40	Mesophile	Neg.	Y	sigmoid rods	freshwater/brackish	fast-growing[1]	I	0.5*5-10	1.475	middle-size	12.173	[3]
5	*Desulfovibrio aespoeensis*	nd	4 to 35	Mesophile	Neg.	Y	Vibrioid-shaped cells	freshwater sediments	fast-growing	I	0.5*1.7-2.5	0.412	small	3.691	[6,7]
6	*Desulfovibrio acrylicus* DSM 10141	nd	nd	Mesophile	Neg.	Y	Small rods to vibrios	marine sediments	fast-growing	I	0.8*2-4	1.508	middle-size	3.691	[8]
7	*Desulfovibrio magneticus* RS-1	nd	nd	Mesophile	Neg.	Y	Vibrio-shaped	freshwater sediments	fast-growing	I	1*3-5	3.142	big	14.137	[9]
8	*Desulfovibrio desulfuricans* subsp. desulfuricans str. ATCC 27774	nd	28 to 44	Mesophile	Neg.	N	Vibrios	animal or plant-associated	fast-growing	I	1*0,25	0.0491	small	0.883	[10-13]
9	*Desulfovibrio gigas* DSM 1382	nd	nd	Mesophile	Neg.	Y	Large curved rods	freshwater sediments	fast-growing	I	1*5-6	4.320	big	18.849	[14,15]
10	*Desulfovibrio piger* ATCC 29098	nd	nd	Mesophile	Neg.	N	rods	animal or plant-associated	fast-growing	I	0.8-1.0*2.5-10	3.976	big	18.943	[16,17]

11	*Desulfovibrio cf. magneticus* IFRC170	nd	nd	Mesophile	Neg.	Y	Vibrio-shaped	freshwater sediments	nd	nd	nd	nd	nd	nd	[9]
12	*Desulfovibrio oxyclinae* DSM 11498	7 to 7.5	nd	Mesophile	Neg.	Y	Straight or curved rods	marine sediments	slow-growing	I	0.5*2-3	0.491	small	4.3196	[18]
13	*Desulfovibrio cuneatus* DSM 11391	7.2 to 7.4	0 to 33	Mesophile	Neg.	Y	Slightly curved rods	marine sediments	slow-growing	I	0.4-0.6*1.6-2.7	0.422	small	3.770	[19]
14	*Desulfovibrio vulgaris* str. Miyazaki F	nd	nd	Mesophile	Neg.	Y	vibrios	freshwater sediments	fast-growing	I	0.8-1.2*2.2-3[2]	2.042	middle-size	9.739	[20,21]
15	*Desulfobulbus elongatus* DSM 2908	6.0 to 7.8	20 to 40	Mesophile	Neg.	Y	Straight to slightly curved rod with pointed ends	impacted or engineered system	fast-growing	I	0.5-0.7*1.5-2.5	0.565	small	4.335	[22]
16	*Desulfocurvus vexinensis* DSM 17965	5.0 to 9.0	20 to 50	Mesophile	Neg.	Y	rods or vibrios	freshwater sediments	fast-growing	I	0.5*3-5	0.785	small	6.676	[23]
17	*Desulfovibrio alkalitolerans*	6.9 to 9.9	16 to 47	Mesophile	Neg.	Y	vibrio-shaped	impacted or engineered system	fast-growing	I	0.5–0.86*1.4–1.9	0.599	small	4.251	[24]
18	*Desulfovibrio fructosivorans* JJ	6.5 - 7	nd	Mesophile	Neg.	Y	curved rods	brackish sediments	fast-growing	I	0.5-0.7*2-4	0.848	small	6.220	[25]
19	*Desulfonatronovibrio hydrogenovorans* DSM 9292	7.0 to 10.2	15 to 43	Mesophile	Neg.	Y	vibrio	brackish sediments	slow-growing	C	0.5*1.5-2	0.344	small	3.141	[26]
20	*Desulfobulbaceae bacterium* BRH_c16a	nd	nd	Mesophile	nd	nd	nd	freshwater sediments	nd	nd	nd	nd	nd	nd	[27]
21	*Desulfobulbus propionicus* type strain (1pr3)	6.0 to 8.6	10 to 43	Mesophile	Neg.	N	Ellipsoidal to lemon-shaped	freshwater/brackish	fast-growing	I	1-1.3*1.8-2	1.973	middle-size	8.941	[28,29]
22	*Desulfovibrio salexigens*	nd	nd	Mesophile	Neg.	Y	Vibrio	marine sediments	nd	I	0.7-2*1-2	2.115	middle-size	9.135	[30,31]
23	*Desulfovibrio frigidus* DSM 17176	6.9 to 7.5	22 to 25	Mesophile	Neg.	Y	rod-shaped or vibrioid	marine sediments	nd	I	2.5-5*0.7	1.443	middle-size	9.016	[32]

24	*Desulfovibrio hydrothermalis* DSM 14728	6.7 to 8.2	20 to 40	Mesophile	Neg.	Y	vibrio-shaped or sigmoid	marine sediments	fast-growing	I	0.5-1*1-2	0.663	small	4.418	[33]
25	*Desulfotomaculum hydrothermale* Lam5	5.8 to 8.2	40 to 60	Thermophiles	Pos.	Y	slightly curved rods	freshwater sediments	nd	I	1*3-6	3.534	big	15.707	[34]
26	*Desulfotomaculum ruminis* DSM 2154	6 to 8.5	30 to 48	Mesophile	Neg.	Y	Rods	animal or plant-associated	fast-growing	I	0.5*3-6	0.883	small	7.461	[35]
27	*Desulfotomaculum reducens* MI-1	7.0 to 7.2	37	Mesophile	Pos.	Y	slightly curved rods	marine sediments	slow-growing	I	0.8-1*5-10	4.771	big	22.477	[36-38]
28	*Desulfovibrio* sp. X2	nd	nd	Mesophile	Neg.	nd	nd	freshwater sediments	nd	nd	nd	nd	nd	nd	[39]
29	*Desulfovibrio* sp. A2	7.2 to 7.4	nd	Mesophile	Neg.	nd	rods	freshwater sediments	fast-growing	nd	0.5-1.5 * 0.4-0.8	0.283	small	2.450	[40,41]
30	*Desulfosporosinus acidiphilus* SJ4	3.6 to 5.5	25 to 40	Mesophile	Neg.	N	curved rods	impacted or engineered system	fast-growing	I	4-7*0.8-1	3.499	big	16.823	[42]
31	*Desulfovibrio aminophilus* DSM 12254	6.7 to 8.0	25 to 40	Mesophile	Neg.	Y	Vibrios	impacted or engineered system	nd	I	0.2*3-4	0.110	small	2.262	[43]
32	*Desulfobacter curvatus* DSM 3379	6.8 to 7.2	28-31	Mesophile	Neg.	Y	vibrio-shaped ceils	marine sediments	slow-growing	C	0.5-1*1.7-3.5	1.149	middle-size	7.009	[44]
33	*Desulfobacter vibrioformis* DSM 8776	6.8 to 7.0	5 to 38	Mesophile	Neg.	Y	Vibrio-shaped	impacted or engineered system	slow-growing	C	1.9-2.3*4.5-8	21.647	big	48.159	[45]
34	*Desulfobacterium anilini* DSM 4660	nd	nd	Mesophile	Neg.	N	rod-shaped	marine sediments	slow-growing	C	1.25*1.5-3	2.761	big	11.290	[46]
35	*Desulfovermiculus halophilus* DSM 18834	6.4 to 8.2	25 to 47	Mesophile	Neg.	Y	vibrios or spirilla	impacted or engineered system	slow-growing	I	0.5–0.6*1–15	1.901	middle-size	14.298	[47]
36	*Desulfovibrio piezophilus* C1TLV30	5.4 to 8.6	15 to 45	Mesophile	Neg.	Y	nonspore-forming vibrios	marine sediments	fast-growing	I	2-4*0.5	0.589	small	5.105	[48]

37	*Desulfomicrobium baculatum* DSM 4028	nd	2 to 41	Mesophile	Neg.	Y	rods	marine sediments	nd	I	1.4-2.9*0.7-0.9	1.081	middle-size	6.409	[49]
38	*Desulfohalobium retbaense*	5.5 to 8.0	nd	Mesophile	Neg.	Y	Straight to slightly curved rods	marine sediments	fast-growing	I	1-3*0.7-0.9	1.005	middle-size	6.032	[50]
39	*Desulfatibacillum aliphaticivorans* DSM 15576	6.6 to 7.8	15 to 40	Mesophile	Neg.	N	slightly curved rods	marine sediments	slow-growing	C	0.6*2.2-5.5	1.088	middle-size	7.822	[51]
40	*Desulfobacterium autotrophicum* HRM2	nd	nd	Mesophile	Neg.	Y	Oval	marine sediments	fast-growing	C	1.2-2.5*1	0.968	small	5.0367	[52]
41	*Desulfobacula* sp. TS	nd	nd	Mesophile	Neg.	nd	nd	impacted or engineered system	nd	nd	nd	nd	nd	nd	[53,54]
42	*Desulfobacula toluolica* Tol2	7 to 7.1	nd	Mesophile	Neg.	Y	coccoid	impacted or engineered system	slow-growing	C	nd	nd	nd	nd	[55]
43	*Desulfotalea psychrophila* LSv54	nd	1,8 to 19	Psychrotrophs	Neg.	Y	Rods	marine sediments	slow-growing	I	0.6*4.5-7.4	1.682	middle-size	11.781	[56]
44	*Desulfovibrio alaskensis* G20[3]	6.5 to 8.5	10 to 45	Mesophile	Neg.	Y	vibrio-shaped cells	impacted or engineered system	fast-growing	I	1-5*0.5-1.2	1.702	middle-size	9.146	[57]
45	*Desulforegula conservatrix* Mb1Pa	nd	25 - 30	Mesophile	Neg.	Y	rod-shaped	freshwater sediments	nd	I	1-1.3*2.6-3	2.908	big	12.193	[58]
46	*Desulfovibrio* sp. FW1012B	nd	nd	Mesophile	Neg.	Y	nd	impacted or engineered system	nd	nd	nd	nd	nd	nd	[59]
47	*Desulfovibrio bastinii* DSM 16055	5.2 to 7.4	20 to 50	Mesophile	Neg.	Y	vibrioid	impacted or engineered system	slow-growing	I	0.5*2-3	0.491	small	4.3196	[60]
48	*Desulfonatronospira thiodismutans*	8.3 to 10.5	28 to 43	Mesophile	Neg.	Y	vibrio-shaped	brackish sediments	slow-growing	I	0.6-0.8*2-3	0.962	small	6.267	[61]
49	*Desulfovibrio alcoholivorans* DSM 5433	5.5 to 8.5	20 to 42	Mesophile	Neg.	Y	curved rods	impacted or engineered system	nd	I	0.7-0.9*2.8-3.2	1.508	middle-size	8.545	[62]

50	*Desulfovibrio desulfuricans* 642[4]	nd	nd	Mesophile	Neg.	Y	Vibrios	impacted or engineered system	nd	I	1*0.25	0.049	small	0.883	[10-12,63]
51	*Desulfobacter postgatei* 2ac9	6.2 to 8.5	10 to 37	Mesophile	Neg.	Y	Rod-shaped to ellipsoidal	marine sediments	slow-growing	C	1-1.5*1.7-2.5	2.577	big	10.701	[45,64]
52	*Desulfotignum phosphitoxidans* DSM 13687	5.0 to 8.0	15 to 30	Mesophile	Neg.	N	Rod-shaped	marine sediments	slow-growing	I	0.6-0.8*2-4	1.154	middle-size	7.367	[65]
53	*Desulfotignum balticum* DSM 7044	6.5 to 8.2	10 to 32	Mesophile	Neg.	Y	short rods	marine sediments	slow-growing	C	0.5-0.7*1.5-3	0.636	small	4.806	[66,67]
54	*Desulfatirhabdium butyrativorans* DSM 18734	6.5 to 8.0	15 to 37	Mesophile	Neg.	nd	lens-shaped	impacted or engineered system	slow-growing	C	1-1.3*2.6-3.5	3.168	big	13.096	[68]
55	*Desulfobulbus japonicus* DSM 18378	6.1 to 7.5	15 to 35	Mesophile	Neg.	Y	rod-shaped	brackish/marine	fast-growing	C	0.8-1.6*1.4-2.9	2.431	middle-size	10.367	[69]
56	*Desulfovibrio zosterae* DSM 11974	nd	nd	Mesophile	Neg.	Y	Curved rods to sigmoid-shaped cells	animal or plant-associated	fast-growing	I	0.5*3	0.589	small	5.105	[70]
57	*Desulfovibrio putealis* DSM 16056	nd	nd	Mesophile	Neg.	Y	Vibrioid	freshwater sediments	fast-growing	I	2.3-4.1*0.7	1.231	middle-size	7.807	[71]
58	*Desulfospira joergensenii* DSM 10085	nd	nd	Mesophile	Neg.	N	curved	marine sediments	nd	C	0.7-0.8*1-2	0.663	small	4.418	[72]
59	*Desulfatitalea* sp. BRH_c12	nd	nd	Mesophile	nd	nd	nd	freshwater sediments	nd	nd	nd	nd	nd	nd	[27]
60	*Desulfovibrio inopinatus* DSM 10711	5.0 to 8.0	15 to 42	Mesophile	Neg.	Y	Vibrioid	marine sediments	slow-growing	I	1-1.5*4-12	9.817	big	33.869	[73]
61	*Desulfobacca acetoxidans* DSM 11109	6.5 to 8.3	27 to 47	Mesophile	Neg.	N	oval to rod-shaped	impacted or engineered system	slow-growing	C	1.3*1.9-2.2	2.721	big	11.027	[74]
62	*Desulfobulbus mediterraneus* DSM 13871	6.3 to 8.0	10 to 35	Mesophile	Neg.	Y	Ovoid	marine sediments	slow-growing	I	1.2-1.7*1.4-3.2	2.496	middle-size	9.211	[75]

63	*Desulfomicrobium escambiense* DSM 10707	nd	nd	Mesophile	Neg.	Y	short rod	freshwater sediments	fast-growing	I	1.7-2.2*0.5	0.382	small	3.456	[76]
64	*Desulfonatronum lacustre* DSM 10312	8.0 to 10.0	22 to 45	Mesophile	Neg.	Y	Vibrio	freshwater sediments	slow-growing	C	0.7-0.9*2.5-3	1.382	middle-size	7.916	[77,78]
65	*Desulfosarcina* sp. BuS5	6.0 to 7.4	10 to 33	Mesophile	Neg.	nd	Rod shape	marine sediments	slow-growing	nd	1-2*1-1.5	1.870	middle-size	8.431	[79]
66	*Desulfovibrio longus* DSM 6739	6.5 to 8.5	10 to 40	Mesophile	Neg.	Y	Long, thin, straight, rod-shaped	impacted or engineered system	fast-growing	I	0.4-0.5*5-10	1.192	middle-size	10.921	[80]
67	*Desulfurella acetivorans* DSM 5264	4.3 to 7.5	44 to 70	Thermophiles	Neg.	Y	short rods with oval ends	freshwater sediments	fast-growing	C	0.5-0.7*1-2	0.424	small	3.393	[81]
68	*Desulfonauticus* sp. A7A	6.2 to 8.6	40 to 64	Thermophiles	Neg.	Y	straight to slightly curved rods	impacted or engineered system	fast-growing	I	0.6*1.2-4	0.735	small	5.466	[82]
69	*Desulfonatronovibrio magnus*	8.5 to 10.5	max. 41	Mesophile	Neg.	Y	vibrio-shaped	brackish sediments	slow-growing	C	0.8-1*2-3	1.590	middle-size	8.341	[77]
70	*Desulfonatronum thioautotrophicum* ASO4-1	8.3 to 10.5	max. 40 – 41	Mesophile	Neg.	Y	vibrio-shaped	brackish sediments	slow-growing	I	0.5-0.6*2-4	0.687	small	5.547	[77]
71	*Desulfonatronum thiodismutans*	8.0 to 10.0	15 to 48	Mesophile	Neg.	Y	vibrio-shaped	brackish sediments	slow-growing	nd	0.6-0.7*1.2-2.7	0.647	small	4.645	[78]
72	*Desulfovibrio desulfuricans* ND132	6.8 to 8.2	30 to 37	Mesophile	Neg.	Y	vibrio-shaped	freshwater sediments	fast-growing	I	0.5-0.7*2-3	0.707	small	5.278	[83]
73	*Desulfococcus multivorans* DSM 2059			Mesophile	Neg.	Y	Coccus	marine sediments	slow-growing	C	1.3-2.5	3.590	big	11.341	[84]
74	*Desulfococcus oleovorans* Hxd3	nd	nd	Mesophile	Neg.	nd	Rod	impacted or engineered system	slow-growing	C	0.4-0.5*0.8-2	0.721	small	4.593	[85]
75	*Desulfobulbus* sp. Dsb1 (Tol1)	nd	nd	Mesophile	nd	nd	nd	animal or plant-associated	nd	nd	nd	nd	nd	nd	[86]

76	*Desulfocapsa sulfexigens* DSM 10523	6.0 to 8.2	4 to 35	Mesophile	Neg.	Y	elongated with slightly pointed ends	marine sediments	slow-growing	nd	0.5*2-4	0.589	small	5.105	[87]
77	*Desulfurivibrio alkaliphilus* AHT 2	8.5 to 10.5	nd	Mesophile	Neg.	N	rod-shaped	brackish sediments	slow-growing	nd	0.4*2.5	0.314	small	3.393	[88]
78	*Thermodesulfatator indicus* DSM 15286	6.0 to 6.7	55 to 80	Thermophiles	Neg.	Y	rods	marine sediments	fast-growing	nd	0.8-1*0.4-0.5	0.143	small	1.590	[89]
79	*Desulfotomaculum acetoxidans* DSM 771	6.6 to 7.6	20 to 40	Mesophile	Neg.	Y	Straight or slightly curved rods	brackish/marine / animal or plant-associated	fast-growing	C	1-1.5*3.5-9	7.670	big	26.997	[90]
80	*Thermodesulfobacterium commune* DSM 2178	6.0 to 8.0	45 to 85	Thermophiles	Neg.	N	Tiny rods	freshwater sediments	fast-growing	I	0.3*0.9	0.064	small	0.989	[91]
81	*Thermodesulfobacterium geofontis* OPF15	5.5 to 8.5	60 to 90	Thermophiles	Neg.	N	short, straight rods	freshwater sediments	fast-growing	nd	2*0.3	0.141	small	2.026	[92]
82	*Desulfocurvibacter africanus* PCS	5.5 to 8	25 to 45	Mesophile	Neg.	Y	curved rods	marine sediments	slow-growing	I	0.3*5-10	0.530	small	7.210	[93]
83	*Syntrophobacter fumaroxidans* MPOB	6.0 to 8.0	20 to 40	Mesophile	Neg.	N	rod- to eye-shaped	impacted or engineered system	slow-growing	I	1.1-1.6*1.8-2.5	3.077	big	11.981	[94]
84	*Desulfarculus baarsii* DSM 2075	6.5 to 8.2	20 to 39	Mesophile	Neg.	Y	vibrio-shaped	freshwater sediments	slow-growing	C	0.5-0.7*1.5-4	0.777	small	5.749	[95,96]
85	*Archaeoglobus sulfaticallidus* PM70-1	6.3 to 7.6	60 to 80	Thermophiles	nd	N	irregular lobes or triangles	marine sediments	nd	I	0.4*2.2	0.184	small	2.188	[97]
86	*Archaeoglobus profundus* DSM 5631	4.5 to 7.5	65 to 90	Thermophiles	Neg.	N	triangular-shaped	marine sediments	fast-growing	nd	1.3*0.75	0.574	small	3.946	[98]
87	*Archaeoglobus fulgidus* DSM 4304	5.5 to 7.5	60 to 95	Thermophiles	nd	Y	Regular to irregular coccoid	marine sediments	fast-growing	I	0.4-1	0.179	small	1.539	[99,100]
88	*Thermodesulfovibrio yellowstonii* DSM 11347	6.5 to 7.7	40 to 70	Thermophiles	Neg.	Y	Curved rods or vibrios	brackish sediments	slow-growing	I	0.3*1.5	0.106	small	1.555	[101]

89	*Candidatus Desulforudis audaxviator* MP104C	nd	nd	Mesophile	Pos.	Y	nd	impacted or engineered system	nd	nd	nd	nd	nd	nd	[102]
90	*Desulfovibrio vulgaris* DP4[5]	nd	nd	Mesophile	Neg.	Y	rods	soil/sediments	fast-growing	nd	0.8-1.5*2-4	3.116	big	12.915	[103]
91	*Desulfatibacillum alkenivorans* DSM 16219	6.2 to 8.0	22 to 38	Mesophile	Neg.	Y	slightly curved rods	impacted or engineered system	slow-growing	C	0.68*1.2-4.5	1.035	middle-size	6.814	[104]

[1]Similar value as *Desulfocurvibacter africanus* DSM 2603. [2]Size was assumed to be the same as *Desulfovibrio vulgaris* str. Hildenborough. [3]Based on info from *Desulfovibrio alaskensis* DSM 16109. [4]Some aspects were assumed to be same as *Desulfocurvibacter desulfuricans* ATCC 27774. [5]Some physiological aspects were assumed to be the same as *Desulfovibrio vulgaris* str. Miyazaki F.

Table S3: Ecophysiological traits of SRP. The columns show the different SRP in this study, pH range, temperature range, classification with respect to temperature, cell wall type (gram), motility (denoted with Y for those for which the flagellum has been observed and N for which it has not been observed), habitat type, growth type under optimal conditions, oxidation (complete or incomplete), size (um^2), cell volume (μm^3) and cell surface area.

No	Strain	n	CRISPR Sequence ID	CRISPR Id	CRISPR Start	CRISPR End	Consensus Repeat	Repeat Length	Spacers	Evidence Level	Total CRISPR Size
7	Desulfovibrio magneticus RS-1	2	gi|239904639|ref|NC_012796.1|	NC_012796_1	1589785	1591828	gtcgccccctgcgcgggggcgtggattgaaac	32	30	4	3272
			gi|239904639|ref|NC_012796.1|	NC_012796_3	4725356	4726585	ttttctgagctgcctatgcggcagtgaac	29	20	4	
8	Desulfovibrio desulfuricans subsp. desulfuricans str. ATCC 27774	1	gi|220903286|ref|NC_011883.1|	NC_011883_1	1083779	1085579	gtgttccccacgggcgtggggatgaaccg	29	29	4	1800
9	Desulfovibrio gigas DSM 1382	1	gi|545640361|ref|NC_022444.1|	NC_022444_7	2555284	2555600	cacccgactattgaagtcgggcctcattgaag	32	4	3	316
10	Desulfovibrio piger ATCC 29098	2	gi|209947786|gb|ABXU01000001.1|	ABXU01000001_1	28213	29888	cggttcatccccacgcgtgtggggaatac	29	27	4	2375
			gi|209947778|gb|ABXU01000009.1|	ABXU01000009_1	17863	18563	gtattccccacgcacgtggggatgaaccg	29	11	4	
11	Desulfovibrio cf. magneticus IFRC170	2	gi|573972682|gb|JAGC01000009.1|	JAGC01000009_1	628799	633388	gtcgccccctgcgcgggggcgtggattgaagc	32	69	4	6203
			gi|573972682|gb|JAGC01000009.1|	JAGC01000009_5	1931181	1932795	gtgttccccgtaggcgcggggatgaaccg	29	26	4	
12	Desulfovibrio oxyclinae DSM 11498	2	gi|481039505|gb|AQXE01000003.1|	AQXE01000003_1	8	878	gttcactgccgcgtaggcagctcagaaa	28	14	4	1496
			gi|481039489|gb|AQXE01000010.1|	AQXE01000010_1	166675	167301	tttctgagctgcctacgcggcagtgaac	28	10	4	
14	Desulfovibrio vulgaris str. Miyazaki F	1	gi|218885109|ref|NC_011769.1|	NC_011769_4	2444693	2448088	gtcgcccctcacgcgggggcgtggatagaaac	32	51	4	3395
15	Desulfobulbus elongatus DSM 2908	1	gi|607834666|gb|JHZB01000008.1|	JHZB01000008_1	130150	130441	gtcgccccttcacgggggcgtggattgaaac	31	4	3	291
16	Desulfocurvus vexinensis DSM 17965	2	gi|573088800|gb|JAEX01000001.1|	JAEX01000001_3	26779	27260	gccccggcgccccgggccaagggc	24	8	2	752
			gi|573088793|gb|JAEX01000003.1|	JAEX01000003_1	211016	211287	cccgcgtgctcgcgcgcccggagataagg	29	4	2	
17	Desulfovibrio alkalitolerans	4	gi|523462140|gb|ATHI01000015.1|	ATHI01000015_1	52	3897	gtttcaatccacgcccccgtgcggggggcgac	32	58	4	9023
			gi|523462085|gb|ATHI01000018.1|	ATHI01000018_1	51	1870	gtcgcccccgcacgggggcgtggattgaaac	32	27	4	

			gi|523462062|gb|ATHI01000019.1|	ATHI01000019_1	52	1862	gtcgccccccgcacgggggcgtggattgaaac	32	27	4	
			gi|523461280|gb|ATHI01000024.1|	ATHI01000024_1	12	1561	gtcgccccccgcacgggggcgtggattgaaac	32	23	4	
18	Desulfovibrio fructosivorans JJ	2	gi|302493090|gb|AECZ01000001.1|	AECZ01000001_1	41517	42405	gtcgcaactcgcgcgtgtgcgggggatgaagg	32	13	4	2563
			gi|302490808|gb|AECZ01000016.1|	AECZ01000016_1	1246	2921	gggactacccccgcgcgagcggggaagac	29	27	4	
19	Desulfonatronovibrio hydrogenovorans DSM 9292	1	gi|631913834|gb|JMKT01000012.1|	JMKT01000012_1	4	391	gtgaaccgccgcataggcggcttagaaa	28	6	4	387
20	Desulfobulbaceae bacterium BRH_c16a	2	gi|780797249|gb|LADS01000002.1|	LADS01000002_1	651	2597	gtcactccccgcgcgggagtgtggattgaaac	32	29	4	14917
			gi|780792556|gb|LADS01000049.1|	LADS01000049_1	319036	332007	gtcactccccgcgcgggagtgtggattgaaac	32	196	4	
21	Desulfobulbus propionicus type strain (1pr3)	1	gi|320352146|ref|NC_014972.1|	NC_014972_1	1701541	1707620	gtcgccccccacgcgggggcgtggattgaaac	32	91	4	6079
24	Desulfovibrio hydrothermalis DSM 14728	2	gi|436839709|ref|NC_020055.1|	NC_020055_4	118127	118736	gtcaaaacccataccgatatggatacctcttttgag	36	8	4	1716
			gi|436839709|ref|NC_020055.1|	NC_020055_6	2574826	2575933	gttcactgccggataggcagcttagaaa	28	18	4	
25	Desulfotomaculum hydrothermale Lam5	4	gi|411455086|emb|CAOS01000004.1|	CAOS01000004_1	71022	75642	gttacaaccattgcccgataaacaaggggactgaaat	37	62	4	9527
			gi|411455050|emb|CAOS01000006.1|	CAOS01000006_1	1348	5087	gtttcaattccttataggtaggctaataac	30	56	4	
			gi|411455050|emb|CAOS01000006.1|	CAOS01000006_2	6735	7552	gtttcaattccttataggtaggctaataac	30	12	4	
			gi|411454671|emb|CAOS01000008.1|	CAOS01000008_1	43280	43631	gtttcaattccttataggtaggctaataac	30	5	4	
26	Desulfotomaculum ruminis DSM 2154	10	gi|334089967|gb|CP002780.1|	CP002780_1	1100345	1102451	gtttcaaatccttataggtaggctagaaac	30	31	4	22298
			gi|334089967|gb|CP002780.1|	CP002780_2	1143003	1143369	gtttcaaatccttataggtaggctagaaac	30	5	4	
			gi|334089967|gb|CP002780.1|	CP002780_4	1421835	1423727	gtcgctctctaggtgagagcgtggattgaaat	32	28	4	
			gi|334089967|gb|CP002780.1|	CP002780_5	1424987	1427147	gtcgctctctaggtgagagcgtggattgaaat	32	32	4	

			gi|334089967|gb|CP002780.1|	CP002780_6	1435556	1439454	gtcgctctctgtacgagagcgtggatttaaac	32	58	4	
			gi|334089967|gb|CP002780.1|	CP002780_7	1800074	1803706	gttactagcctacctataaggatttgaaac	30	54	4	
			gi|334089967|gb|CP002780.1|	CP002780_8	2490237	2491527	gtttttagcttacctataaggatttgaaac	30	19	4	
			gi|334089967|gb|CP002780.1|	CP002780_10	3057773	3061057	gtttcaaatccttataggtagactagtaac	30	49	4	
			gi|334089967|gb|CP002780.1|	CP002780_11	3091332	3093024	gtttttagcctacctataagggattgaaac	30	25	4	
			gi|334089967|gb|CP002780.1|	CP002780_12	3099787	3101765	gtttctagcttacctataaggatttgaaac	30	29	4	
27	Desulfotomaculum reducens MI-1	4	gi|134050581|gb|CP000612.1|	CP000612_1	586379	587743	gttatcagcctacctataaggaattgaaac	30	20	4	6608
			gi|134050581|gb|CP000612.1|	CP000612_2	1079904	1083390	gtcgcccctcacgcaggggcgtggattgaaac	32	52	4	
			gi|134050581|gb|CP000612.1|	CP000612_4	1764821	1766001	gtcaaaacacaaaataattccctttgggaattgaaac	37	15	4	
			gi|134050581|gb|CP000612.1|	CP000612_5	1768139	1768717	gtcaaaacacaaaataattccctttgggaattgaaac	37	7	4	
28	Desulfovibrio sp. X2	1	gi|523471633|gb|ATHV01000015.1|	ATHV01000015_1	54654	58637	gtcgcccctcgcacaggggcgtggattgaaac	32	60	4	3983
29	Desulfovibrio sp. A2	1	gi|347517814|gb|AGFG01000055.1|	AGFG01000055_1	37	554	ggcctatccccgcaggcgcgggggaac	28	8	4	517
30	Desulfosporosinus acidiphilus SJ4	1	gi|391353422|gb|CP003639.1|	CP003639_1	1538047	1540340	gtcgctccctacgtgggagcgtggattgaaat	32	34	4	2293
32	Desulfobacter curvatus DSM 3379	2	gi|480985097|gb|AREY01000003.1|	AREY01000003_1	129598	131150	gtttcaatccacgctcccgcatggggagcgac	32	23	4	2173
			gi|480985033|gb|AREY01000024.1|	AREY01000024_1	7348	7969	gtttcaattccacgttggtctgattaaaag	30	9	4	
33	Desulfobacter vibrioformis DSM 8776	1	gi|675284502|gb|JQKJ01000013.1|	JQKJ01000013_1	70505	72652	ttcacaatccctcatggggatagataac	28	33	4	2147
34	Desulfobacterium anilini DSM 4660	1	gi|523385901|gb|AULM01000007.1|	AULM01000007_1	158972	159548	gtttcccccgcgcatgcggggatcgacc	28	9	4	576
38	Desulfohalobium retbaense	1	gi|258404138|ref|NC_013223.1|	NC_013223_1	1876848	1877552	gcttcaatggggccacactttgcagtgtggttgagac	37	9	4	704
39	Desulfatibacillum aliphaticivorans DSM 15576	1	gi|218777860|ref|NC_011768.1|	NC_011768_4	6093236	6097299	gtcttccccacgcccgtgggggtgtttct	29	66	4	4063

40	Desulfobacterium autotrophicum HRM2	1	gi|224367124|ref|NC_012108.1|	NC_012108_1	629857	636222	cctggaatgcctgatccacgacaacaaggattgaaac	37	87	4	6365
41	Desulfobacula sp. TS	2	gi|667676329|gb|JIBV01000103.1|	JIBV01000103_1	2052	3985	gtctgaacaccatatccagaagaacaaggattgaaac	37	26	4	2624
			gi|667676328|gb|JIBV01000104.1|	JIBV01000104_1	67	758	gtctgaacaccatatccagaagaacaaggattgaaac	37	9	4	
42	Desulfobacula toluolica Tol2	3	gi|408417460|ref|NC_018645.1|	NC_018645_1	1120959	1125463	cttttaatcagaccagtgtggaattgaaac	30	68	4	5418
			gi|408417460|ref|NC_018645.1|	NC_018645_2	1127872	1128164	cttttaatcagaccagtgtggaattgaaac	30	4	4	
			gi|408417460|ref|NC_018645.1|	NC_018645_4	3489798	3490420	gtctgaacggcatatccagaagaacaaggattgaaac	37	8	4	
43	Desulfotalea psychrophila LSv54	1	gi|51243852|ref|NC_006138.1|	NC_006138_2	198142	199100	gcttcaatgtagtcacccctttcgaggtgattgatac	37	13	4	958
44	Desulfovibrio alaskensis DSM 16109	1	gi|78355047|ref|NC_007519.1|	NC_007519_1	885036	886223	cggttcatccccgcgggtgcggggaacac	29	19	4	1187
45	Desulforegula conservatrix Mb1Pa	1	gi|523394517|gb|AUEY01000013.1|	AUEY01000013_1	58842	62107	gtttcaatccacgctcccgcgcggggagcgac	32	49	4	3265
46	Desulfovibrio sp. FW1012B	1	gi|357632130|ref|NZ_CM001368.1|	NZ_CM001368_4	1487613	1491182	gtgttccccgcaggcgcggggatgatccg	29	58	4	3569
47	Desulfovibrio bastinii DSM 16055	5	gi|523620996|gb|AUCX01000005.1|	AUCX01000005_1	70064	70392	tttctaagctgcctatccggcagtgaac	28	5	4	1793
			gi|523620975|gb|AUCX01000012.1|	AUCX01000012_1	2	270	tttctaagctgcctatccggcagtgaac	28	4	3	
			gi|523620975|gb|AUCX01000012.1|	AUCX01000012_2	7887	8205	ctcaaaagaggtttccgatctggatcgggttttgaca	37	4	3	
			gi|523620975|gb|AUCX01000012.1|	AUCX01000012_3	11471	11788	ctcaaaagaggtttccgatctggatcgggttttgaca	37	4	4	
			gi|523620944|gb|AUCX01000024.1|	AUCX01000024_1	562	1124	tttctaagctgcctatccggcagtgaac	28	9	4	
48	Desulfonatronospira thiodismutans	5	gi|298510264|gb|ACJN02000002.1|	ACJN02000002_1	277891	280378	gtgatccaccgcataggtggctaagaaa	28	41	4	8502
			gi|298510264|gb|ACJN02000002.1|	ACJN02000002_2	729341	730304	gtccgaaggactggcagggtttagaaccattgcgac	36	13	4	
			gi|298510264|gb|ACJN02000002.1|	ACJN02000002_3	736657	737407	gtccgaaggactggctgggtttagaaccattgcggc	36	10	4	
			gi|298510264|gb|ACJN02000002.1|	ACJN02000002_4	1140480	1143097	gtcgccccctgcgcgggggcgtggattgaaac	32	39	4	

			gi|298509225|gb|ACJN02000003.1|	ACJN02000003_5	1042604	1044289	gcttcaatggagccacatttttaatgtggatgagac	37	23	4	
49	Desulfovibrio alcoholivorans DSM 5433	1	gi|651606202|gb|JNJA01000008.1|	JNJA01000008_1	61423	62524	gtgctcaacgcttttcagcatcgaagatggaatcac	36	15	4	1101
51	Desulfobacter postgatei 2ac9	3	gi|387144458|gb|AGJR02000013.1|	AGJR02000013_1	325928	326468	gtctcaatcctctctaatcgaggcaatctttcggac	36	7	4	8661
			gi|387144458|gb|AGJR02000013.1|	AGJR02000013_2	336571	337833	gtctcaatcctctctaatcgaggcaatctttcggac	36	12	4	
			gi|387144457|gb|AGJR02000014.1|	AGJR02000014_1	27231	34090	cggatcatccccacgggggtggggaaaag	29	112	4	
52	Desulfotignum phosphitoxidans DSM 13687	2	gi|474488791|gb|APJX01000003.1|	APJX01000003_1	373490	374536	gtctcaatcctctctaatcgaggcggtgattcggac	36	14	4	2008
			gi|474487091|gb|APJX01000007.1|	APJX01000007_1	11184	12146	gtcgctccccatgcgggagcgtggattgaaac	32	14	4	
53	Desulfotignum balticum DSM 7044	2	gi|522811039|gb|ATWO01000001.1|	ATWO01000001_1	2611973	2614198	gcttcaatggggccggagctgatatgctccggaaaac	37	30	4	4692
			gi|522811039|gb|ATWO01000001.1|	ATWO01000001_4	3498968	3501435	ggaacacccccacaggcgtggggaagat	28	40	4	
54	Desulfatirhabdium butyrativorans DSM 18734	1	gi|523620767|gb|AUCU01000018.1|	AUCU01000018_1	21190	21949	ggtcgatccccgcgtgtgcgggggagcc	28	12	4	759
56	Desulfovibrio zosterae DSM 11974	1	gi|523389953|gb|AUDC01000019.1|	AUDC01000019_1	85377	85757	gttgaatgattctgcgtaatgacagcagattgaaac	36	5	4	380
59	Desulfatitalea sp. BRH_c12	1	gi|780826867|gb|LADR01000053.1|	LADR01000053_1	38009	47735	cggttcatccccgcgtctgcggggaacac	29	159	4	9726
60	Desulfovibrio inopinatus DSM 10711	2	gi|523618915|gb|AUBP01000007.1|	AUBP01000007_1	172429	173377	gtttcaatccacgcacccgcgtggggtgcgac	32	14	4	1289
			gi|523618538|gb|AUBP01000078.1|	AUBP01000078_1	64	405	tcgtctccttcgccggtattgat	23	5	2	
61	Desulfobacca acetoxidans DSM 11109	4	gi|328951746|ref|NC_015388.1|	NC_015388_1	87673	94203	gtttcaatccttgttttagtggatgtatcgccaaaag	37	89	4	9397
			gi|328951746|ref|NC_015388.1|	NC_015388_9	1488840	1490146	cttacaacgataagaagagtcttcggacgattgcgac	37	17	4	
			gi|328951746|ref|NC_015388.1|	NC_015388_11	1934754	1935816	ctcagaaacattgacctgattagaagggattgcgac	36	14	4	
			gi|328951746|ref|NC_015388.1|	NC_015388_14	3066547	3067046	gtcgcaatcccttcgtaatcaggtcaatgaagcagag	37	6	4	

62	Desulfobulbus mediterraneus DSM 13871	1	gi|523620997|gb|AUCW01000012.1|	AUCW01000012_1	164045	164508	gcttcaatgtagccacctcctccgaggtggttggtac	37	6	4	463
64	Desulfonatronum lacustre DSM 10312	2	gi|573089367|gb|JAFE01000001.1|	JAFE01000001_1	387972	389525	cggttcatccccacgtgtgtggggaacgc	29	25	4	2859
			gi|573089362|gb|JAFE01000002.1|	JAFE01000002_1	313754	315060	gtttcaatccacgcccccgcgcggggggcgac	33	19	4	
65	Desulfosarcina sp. BuS5	2	gi|548693802|gb|AXAM01000095.1|	AXAM01000095_1	3835	4858	cttttaatcagaccagaatggaattgaaac	30	15	4	1913
			gi|548693795|gb|AXAM01000096.1|	AXAM01000096_1	4	894	cttttaatcagaccagaatggaattgaaac	30	13	4	
66	Desulfovibrio longus DSM 6739	2	gi|522808638|gb|ATVA01000003.1|	ATVA01000003_2	75640	75909	ttcatccccgcgcacgcggggaacac	26	4	3	1455
			gi|522808608|gb|ATVA01000015.1|	ATVA01000015_1	364054	365240	attacactgcatgacccgctagaaaggggattgcgac	37	15	4	
67	Desulfurella acetivorans DSM 5264	3	gi|754092674|ref|NZ_CP007051.1|	NZ_CP007051_1	545525	549853	gttttagcccccaattgtgggatttaaag	29	66	4	5839
			gi|754092674|ref|NZ_CP007051.1|	NZ_CP007051_2	659173	659971	gtcgtaattcccttcaaaacgggaaagaatgcaac	35	10	4	
			gi|754092674|ref|NZ_CP007051.1|	NZ_CP007051_3	1684621	1685334	gttgcattctttcccgttttgaagggaattacgac	35	9	4	
69	Desulfonatronovibrio magnus	2	gi|761631872|gb|JYNP01000039.1|	JYNP01000039_1	57578	57903	gtctcaatcccctcaagtcggggcaatgtttctgac	36	4	4	576
			gi|761631790|gb|JYNP01000074.1|	JYNP01000074_1	287	538	cagtttggctcctgaaaggttggctcc	27	5	2	
70	Desulfonatronum thioautotrophicum ASO4-1	1	gi|761631699|gb|JYNO01000016.1|	JYNO01000016_1	18293	18690	agtccgaaagcgggttgcgatttgggggctttgagatc	38	5	2	397
71	Desulfonatronum thiodismutans	3	gi|665768126|gb|JPIK01000006.1|	JPIK01000006_1	89016	89346	gtctcggcaagcttggtcagtgttgggtgattggcac	37	4	4	3477
			gi|665768112|gb|JPIK01000020.1|	JPIK01000020_1	72797	73705	gtcgccccccgcgcgggggggcgtggattgaaac	33	13	4	
			gi|665768104|gb|JPIK01000028.1|	JPIK01000028_1	10844	13083	gttttccggggcattcctgccccggcctcattgaagc	37	30	4	
72	Desulfovibrio desulfuricans ND132	1	gi|376294792|ref|NC_016803.1|	NC_016803_5	3491653	3498191	gtcgccccccacgcgggggcgtggattgaaac	32	98	4	6538
73	Desulfococcus multivorans DSM 2059	6	gi|523473342|gb|ATHJ01000001.1|	ATHJ01000001_1	56	533	gtctgaaccactgccccgattcgaggggattgagac	36	6	4	11090

			gi|523473333|gb|ATHJ01000003.1|	ATHJ01000003_2	5694	7043	gtctgaaccactgccccgattcgagggattgagac	36	18	4	
			gi|523473330|gb|ATHJ01000004.1|	ATHJ01000004_1	52	1813	gtctcaatcccctcgaatcggggcagtggttcagac	36	23	4	
			gi|523468025|gb|ATHJ01000087.1|	ATHJ01000087_1	51699	57633	ccgcaccgatctccgatgccgaaaggcgttgagcac	36	83	4	
			gi|523461284|gb|ATHJ01000127.1|	ATHJ01000127_1	46953	47499	gtctgaaccactgccccgattcgagggattgagac	36	7	4	
			gi|523459347|gb|ATHJ01000145.1|	ATHJ01000145_1	3273	4296	gtctgaaccattgccccgattcgagggattgagac	36	13	4	
74	Desulfococcus oleovorans Hxd3	2	gi|158520017|ref|NC_009943.1|	NC_009943_1	860153	861365	gtcgcaatcccctctaacagcgggtctcttccttc	35	16	4	3680
			gi|158520017|ref|NC_009943.1|	NC_009943_8	3590108	3592576	atgttccccacacgtgtggggatgaaccg	29	40	4	
75	Desulfobulbus sp. Dsb1 (Tol1)	4	gi|700403941|gb|JROS01000049.1|	JROS01000049_1	7	2520	cccggaacgaatgccccgatcagaggggattgagac	36	34	4	10165
			gi|700403923|gb|JROS01000050.1|	JROS01000050_1	25099	26732	cccggaacgaatgccccgatcagaggggattgagac	36	22	4	
			gi|700403522|gb|JROS01000061.1|	JROS01000061_1	51	1039	cttcactgccgcacaggcagctgagaaa	28	16	4	
			gi|700403118|gb|JROS01000084.1|	JROS01000084_1	10259	15290	ccgttccccgcacccgcggggatgaaccg	29	82	4	
76	Desulfocapsa sulfexigens DSM 10523	1	gi|451945650|ref|NC_020304.1|	NC_020304_1	3932262	3932826	gtttcaatccacgcccccgcatggagggcgac	32	8	4	564
77	Desulfurivibrio alkaliphilus AHT 2	2	gi|297567992|ref|NC_014216.1|	NC_014216_2	1780099	1782570	cggttcatccccgcgagtgcggggaacat	29	40	4	9413
			gi|297567992|ref|NC_014216.1|	NC_014216_3	1785014	1791956	tttctgagctgcctgtgcggcagtgaac	28	115	4	
78	Thermodesulfatator indicus DSM 15286	3	gi|335358205|gb|CP002683.1|	CP002683_1	238340	242182	gttcacagcctaactaaaaggaatggaaac	30	57	4	9249
			gi|335358205|gb|CP002683.1|	CP002683_4	918034	919769	gtgagaaaaccttgcctgattaagaaggcattacgac	37	22	4	
			gi|335358205|gb|CP002683.1|	CP002683_5	1410352	1414024	gtgagaaaaccttgcctgattaagaaggcattacgac	37	47	4	
79	Desulfotomaculum acetoxidans DSM 771	11	gi|257777071|gb|CP001720.1|	CP001720_1	933526	937981	gatctaatttagccatagtggaatgtaaat	30	67	4	28109
			gi|257777071|gb|CP001720.1|	CP001720_2	940048	943825	gatctaatttagccatagtggaatgtaaat	30	57	4	

			gi|257777071|gb|CP001720.1|	CP001720_3	2198816	2202288	gtttttagcctacctataaggaattgaaat	30	52	4	
			gi|257777071|gb|CP001720.1|	CP001720_4	2203726	2204020	gtttttagcctacctataaggaattgaaat	30	4	3	
			gi|257777071|gb|CP001720.1|	CP001720_5	2217925	2219152	gtttttagcctacctatgagggattgaaat	30	18	4	
			gi|257777071|gb|CP001720.1|	CP001720_6	3161588	3162074	agtttcaattccctatgggaattattttgttttcagac	38	6	4	
			gi|257777071|gb|CP001720.1|	CP001720_7	3171662	3171997	gtttcaattccctacgggaattattttgttttcagat	37	4	3	
			gi|257777071|gb|CP001720.1|	CP001720_8	3189932	3199658	atttcaatacatcaaatgtgacggtttccc	30	147	4	
			gi|257777071|gb|CP001720.1|	CP001720_9	3748348	3749510	gtttttagtctacctatgaggaattgaaac	30	17	4	
			gi|257777071|gb|CP001720.1|	CP001720_10	3834582	3836462	atttcaatccacgcccccgtgtagggagcgac	32	28	4	
			gi|257777071|gb|CP001720.1|	CP001720_13	4451140	4452435	atttcaattcctcatagataggctaaaaac	30	19	4	
80	Thermodesulfobacterium commune DSM 2178	2	gi|668351138|gb|CP008796.1|	CP008796_1	1356859	1357792	gtagaaacctgccctacttcaaaagggattgcgac	35	12	4	2365
			gi|668351138|gb|CP008796.1|	CP008796_2	1461789	1463221	gttttgagcctacctacaaggaattgaaac	30	21	4	
81	Thermodesulfobacterium geofontis OPF15	2	gi|334901311|gb|CP002829.1|	CP002829_1	1115343	1118449	gttgaaacctgccctggattaaaagggattgcgaa	35	41	4	6662
			gi|334901311|gb|CP002829.1|	CP002829_2	1221572	1225128	gtttcaatccaccaaagaggaatttaaac	29	54	4	
83	Syntrophobacter fumaroxidans MPOB	2	gi|116696516|gb|CP000478.1|	CP000478_10	1684126	1689168	gacggagacagagccccgatttgaggggattgagac	36	69	4	9893
			gi|116696516|gb|CP000478.1|	CP000478_17	3489022	3493873	gtgttccccacgcgggtggggatgaaccg	29	79	4	
84	Desulfarculus baarsii DSM 2075	4	gi|301638057|gb|CP002085.1|	CP002085_2	1844781	1865928	gtcgcccctcacgcaggggcgtggattgaaac	32	318	4	23168
			gi|301638057|gb|CP002085.1|	CP002085_3	1894474	1895008	gtttcaatccgcttcctctagcggcatccctcaatag	37	7	4	
			gi|301638057|gb|CP002085.1|	CP002085_4	1902297	1902763	gtttcaatccgcttcctctagcggcatccctcaatag	37	6	4	
			gi|301638057|gb|CP002085.1|	CP002085_10	3332441	3333462	ggcgcgggctccagcggcggcggcaacatcctctacaactacggctcggt	50	10	2	

85	Archaeoglobus sulfaticallidus PM70-1	1	gi|484591393|gb|CP005290.1|	CP005290_2	1137912	1142191	ctttcaatcctacaatagtctgattttaac	30	62	4	4279
87	Archaeoglobus fulgidus DSM 4304	3	gi|6626247|gb|AE000782.1|	AE000782_1	148	4213	gttgaaatcagaccaaaatgggattgaaag	30	59	4	10537
			gi|6626247|gb|AE000782.1|	AE000782_2	398369	401590	ctttcaatcccattttggtctgatttcaac	30	47	4	
			gi|6626247|gb|AE000782.1|	AE000782_3	1690930	1694181	gtaagaaagggaggctcctgaaaatggagattgaaag	37	43	4	
88	Thermodesulfovibrio yellowstonii DSM 11347	3	gi|206741110|gb|CP001147.1|	CP001147_2	632030	633918	gtaggaaacaagacctcatttaaaagggattgcgac	36	26	4	11897
			gi|206741110|gb|CP001147.1|	CP001147_3	1074817	1076909	gttttgagcctacctatgaggaattgaaac	30	31	4	
			gi|206741110|gb|CP001147.1|	CP001147_5	1929305	1937222	ctttatatcccacatggttcagatgaaac	29	120	4	
89	Candidatus Desulforudis audaxviator MP104C	4	gi|169637063|gb|CP000860.1|	CP000860_2	1355523	1359324	ctttcagtccccttttcgtcgggtcggtcgctgaaac	37	51	4	14257
			gi|169637063|gb|CP000860.1|	CP000860_4	1898301	1900891	gtttcaatccctcgtaggtaggctggaaac	30	38	4	
			gi|169637063|gb|CP000860.1|	CP000860_5	1902525	1904408	gtttcaatccctcgtaggtaggctggaaac	30	28	4	
			gi|169637063|gb|CP000860.1|	CP000860_6	1906089	1912072	gtttcaatccctcgtaggtaggctggaaac	30	90	4	
91	Desulfatibacillum alkenivorans DSM 16219	1	NZ_FQZU01000026.1	NZ_FQZU01000026_1	46146	48681	ctcataacccctcatggggatggataac	28	39	4	2535

Table S4: List of CRISPR elements found by CRISPRCas-Finder. The columns indicate the following: The number assigned to each strain in this study, strain name, CRISPR sequence ID, CRISPR ID (named by CRISPRCas-Finder), beginning and end of each CRISPR array, consensus repeat, repeat length, number of spacers, level of evidence and the total size of each CRISPR found.

Strain name	Prophage Sequence ID	Start	End	CRISPR Sequence ID	Start	End
Desulfovibrio fructosivorans JJ	|18R1|gi|302493090|gb|AECZ01000001.1|ctg00049|320|75894	320	75894	gi|302493090|gb|AECZ01000001.1|	41517	42405
Desulfotomaculum ruminis DSM 2154	|26R1|gi|334089967|gb|CP002780.1|Complete Genome|1102652|1163694	1102652	1163694	gi|334089967|gb|CP002780.1|	1143003	1143369
Desulfotomaculum reducens MI-1	|27R1|gi|134050581|gb|CP000612.1|Complete Genome|532811|592446	532811	592446	gi|134050581|gb|CP000612.1|	586379	587743
Desulfurella acetivorans DSM 5264	|67R1|gi|754092674|ref|NZ_CP007051.1|Complete Genome|1671975|1693619	1671975	1693619	gi|754092674|ref|NZ_CP007051.1|	545525	549853
Desulfotomaculum acetoxidans DSM 771	|79R4|gi|257777071|gb|CP001720.1|Complete Genome|4396496|4458901	4396496	4458901	gi|257777071|gb|CP001720.1|	4451140	4452435

Table S5: List of CRISPR elements found inside prophages. The columns indicate the strain name, the prophage sequence ID, the beginning and end of the prophage in the genome, CRISPR sequence ID and the beginning and end of CRISPR in the nucleotide sequence.

Prophage ID	Classification by Head-neck-tail module genes	bp	Strain name
1R1	Myoviridae of Type1 (Cluster 6)	63769	Desulfovibrio vulgaris str. Hildenborough
1R2	Myoviridae of Type1 (Cluster 8)	71645	
1R3	Siphoviridae of Type1 (cluster 6)	63915	
1R4	Podoviridae of Type3	39266	
1R5	Myoviridae of Type1 (Cluster 8)	69018	
1R6	Myoviridae of Type1 (Cluster 6)	52813	
2R1	Myoviridae of Type1 (Cluster 8)	32936	Desulfocurvibacter africanus DSM 2603
2R2	Myoviridae of Type1 (Cluster 6)	28418	
2R3	Myoviridae of Type1 (Cluster 9)	38770	
4R1	Myoviridae of Type1 (Cluster 8)	59873	Desulfocurvibacter africanus str. Walvis Bay
4R2	Myoviridae of Type1 (Cluster 6)	36762	
4R3	Myoviridae	24510	
5R1	Myoviridae of Type1 (Cluster 9)	70566	Desulfovibrio aespoeensis Aspo-2
5R2	Myoviridae	58082	
5R3	Myoviridae of Type1 (Cluster 8)	36074	
8R1	Myoviridae of Type1 (Cluster 9)	47344	Desulfovibrio desulfuricans subsp. desulfuricans str. ATCC 27774
9R1	Siphoviridae of Type1 (Cluster 8)	54387	Desulfovibrio gigas DSM 1382
10R1	Myoviridae of Type1 (Cluster 6)	38173	Desulfovibrio piger ATCC 29098
13R1	Myoviridae	55980	Desulfovibrio cuneatus DSM 11391
14R1	Myoviridae of Type1 (Cluster 8)	60022	Desulfovibrio vulgaris str. Miyazaki
14R2	Siphoviridae of Type1 (Cluster 3)	61831	
15R1	Myoviridae of Type1 (Cluster 6)	51771	Desulfobulbus elongatus DSM 2908
16R1	Myoviridae of Type1 (Cluster 8)	145788	Desulfocurvus vexinensis DSM 17965
17R1	Myoviridae of Type1 (Cluster 8)	34073	Desulfovibrio alkalitolerans

18R1	Siphoviridae of Type1 (No Cluster Assigned)	75574	Desulfovibrio fructosivorans JJ
18R2	Siphoviridae of Type1 (Cluster 8)	93203	
18R3	Myoviridae of Type1 (Cluster 8)	49343	
19R1	Myoviridae of Type1 (Cluster 8)	27600	Desulfonatronovibrio hydrogenovorans DSM 9292
21R1	Siphoviridae of Type1 (Cluster 6)	79843	Desulfobulbus propionicus type strain 1pr3
21R2	Siphoviridae of Type1 (Cluster 8)	87012	
22R1	Myoviridae of Type1 (Cluster 6)	37163	Desulfovibrio salexigens
25R1	Siphoviridae of Type1 (Cluster 2)	45396	Desulfotomaculum hydrothermale Lam5
25R2	Siphoviridae of Type1 (Cluster 2)	60002	
26R1	Podoviridae	61042	Desulfotomaculum ruminis DSM 2154
26R2	Myoviridae of Type1 (Cluster 2)	84530	
27R1	unclassified	59635	Desulfotomaculum reducens MI-1
27R2	Myoviridae of Type1 (Cluster 6)	51840	
27R3	Siphoviridae of Type1 (Cluster 2)	64377	
28R1	Myoviridae of Type1 (Cluster 9)	32022	Desulfovibrio sp. X2
29R1	Myoviridae	24808	Desulfovibrio sp. A2
30R1	Myoviridae of Type1 (Cluster 6)	169593	Desulfosporosinus acidiphilus SJ4
33R1	Siphoviridae of Type1 (Cluster 8)	25102	Desulfobacter vibrioformis DSM 8776
34R1	Siphoviridae of Type1 (Cluster 8)	47057	Desulfobacterium anilini DSM 4660
35R1	unclassified	26392	Desulfovermiculus halophilus DSM 18834
37R1	unclassified	36767	Desulfomicrobium baculatum DSM 4028
38R1	unclassified	55128	Desulfohalobium retbaense
39R1	unclassified	36123	Desulfatibacillum alkenivorans AK-01
43R1	Siphoviridae	52191	Desulfotalea psychrophila LSv54
43R2	Podoviridae of Type3	28810	
44R1	Myoviridae	64002	Desulfovibrio alaskensis G20
44R2	unclassified	110648	
44R3	Myoviridae of Type1 (Cluster 8)	49658	

46R1	Siphoviridae of Type1 (Cluster 3)	71287	Desulfovibrio sp. FW1012B
47R1	Myoviridae	20567	Desulfovibrio bastinii DSM 16055
49R1	Siphoviridae of Type1 (Cluster 5)	121316	Desulfovibrio alcoholivorans DSM 5433
51R1	unclassified	40731	Desulfobacter postgatei 2ac9
52R1	unclassified	15798	Desulfotignum phosphitoxidans DSM 13687
53R1	unclassified	53248	Desulfotignum balticum DSM 7044
53R2	unclassified	55125	
54R1	unclassified	38383	Desulfatirhabdium butyrativorans DSM 18734
57R1	Myoviridae of Type1 (Cluster 8)	23512	Desulfovibrio putealis DSM 16056
59R1	unclassified	13123	Desulfatitalea sp. BRH_c12
60R1	Podoviridae of Type3	32873	Desulfovibrio inopinatus DSM 10711
60R2	Podoviridae of Type3	38072	
61R1	unclassified	42218	Desulfobacca acetoxidans DSM 11109
61R2	Siphoviridae of Type1 (Cluster 8)	32970	
67R1	unclassified	21644	Desulfurella acetivorans DSM 5264
72R1	unclassified	50980	Desulfovibrio desulfuricans ND132
74R1	unclassified	76813	Desulfococcus oleovorans Hxd3
77R1	unclassified	73569	Desulfurivibrio alkaliphilus AHT 2
79R1	unclassified	63473	Desulfotomaculum acetoxidans DSM 771
79R2	unclassified	63482	
79R3	unclassified	61659	
79R4	Myoviridae of Type1 (No Cluster Assigned)	62405	
80R1	Myoviridae of Type1 (Cluster 9)	64161	Thermodesulfobacterium commune DSM 2178
83R1	Siphoviridae of Type1 (Cluster 8)	59396	Syntrophobacter fumaroxidans MPOB
84R1	unclassified	83399	Desulfarculus baarsii DSM 2075
89R1	unclassified	76687	Candidatus Desulforudis audaxviator MP104C

90R1	Siphoviridae of Type1 (Cluster 6)	81648	Desulfovibrio vulgaris DP4
90R2	Myoviridae of Type1 (Cluster 9)	96187	
90R3	Myoviridae of Type1 (Cluster 9)	72305	

Table S6: Classification of prophages according to Virfam. Columns indicate the following: Prophage ID, classification assigned by Virfam using head-neck-tail module genes, the length of the prophage (bp) and the name of the host.

This table is available at https://github.com/robertoorellanar/Prophages_in_SRP.git

Table S7: Pairwise amino acid comparison of all predicted open reading frames (ORFs) from the SRP prophages. The abbreviation used is the following: p1 and p2, correspond to orfs for each prophage, pident, percentage of identical matches, length, alignment length, mismatch, number of mismatches, gaopen, number of gap openings, qstart, start of alignment in query, qend, end of alignment in query, sstart, start of alignment in subject, send, end of alignment in subject, and evalue, expected value.
